# EphB4 and ephrinB2 act in opposition in the head and neck tumor microenvironment

**DOI:** 10.1038/s41467-022-31124-7

**Published:** 2022-06-20

**Authors:** Shilpa Bhatia, Diemmy Nguyen, Laurel B. Darragh, Benjamin Van Court, Jaspreet Sharma, Michael W. Knitz, Miles Piper, Sanjana Bukkapatnam, Jacob Gadwa, Thomas E. Bickett, Shiv Bhuvane, Sophia Corbo, Brian Wu, Yichien Lee, Mayumi Fujita, Molishree Joshi, Lynn E. Heasley, Robert L. Ferris, Olga Rodriguez, Christopher Albanese, Mohit Kapoor, Elena B. Pasquale, Sana D. Karam

**Affiliations:** 1 Department of Radiation Oncology, University of Colorado Denver, Anschutz Medical Campus, Aurora, CO USA; 2 Krembil Research Institute, University Health Network and University of Toronto, Toronto, ON Canada; 3 Department of Oncology, Lombardi Comprehensive Cancer Center, Georgetown University Medical Center, Washington, DC USA; 4 Department of Dermatology, University of Colorado Denver, Anschutz Medical Campus, Aurora, CO USA; 5 Department of Pharmacology, University of Colorado Denver, Anschutz Medical Campus, Aurora, CO USA; 6 Department of Craniofacial Biology, School of Dental Medicine, University of Colorado Denver, Anschutz Medical Campus, Aurora, CO USA; 7 Department of Otolaryngology, University of Pittsburgh, Pittsburgh, PA USA; 8 Department of Immunology, University of Pittsburgh School of Medicine, Pittsburgh, PA USA; 9 UPMC Hillman Cancer Center, Pittsburgh, PA USA; 10 Cancer Center, Sanford Burnham Prebys Medical Discovery Institute, La Jolla, CA USA

**Keywords:** Cancer, Head and neck cancer, Head and neck cancer

## Abstract

Differential outcomes of EphB4-ephrinB2 signaling offers formidable challenge for the development of cancer therapeutics. Here, we interrogate the effects of targeting EphB4 and ephrinB2 in head and neck squamous cell carcinoma (HNSCC) and within its microenvironment using genetically engineered mice, recombinant constructs, pharmacologic agonists and antagonists. We observe that manipulating the EphB4 intracellular domain on cancer cells accelerates tumor growth and angiogenesis. EphB4 cancer cell loss also triggers compensatory upregulation of EphA4 and T regulatory cells (Tregs) influx and their targeting results in reversal of accelerated tumor growth mediated by EphB4 knockdown. EphrinB2 knockout on cancer cells and vasculature, on the other hand, results in maximal tumor reduction and vascular normalization. We report that EphB4 agonism provides no additional anti-tumoral benefit in the absence of ephrinB2. These results identify ephrinB2 as a tumor promoter and its receptor, EphB4, as a tumor suppressor in HNSCC, presenting opportunities for rational drug design.

## Abstract (web summary)

EphrinB2 and its receptor EphB4 are highly expressed in head and neck squamous cell carcinoma (HNSCC) and disrupting EphB4-ephrinB2 interaction generates sub-optimal outcomes. Here, compartmental targeting of EphB4 and ephrinB2 in HNSCC cancer cell and endothelial compartments suggests that ephrinB2 acts as a tumor promoter and EphB4 as a tumor suppressor.

## Introduction

The Eph receptors, which constitute the largest family of receptor tyrosine kinases (RTKs) along with their membrane-anchored ligands, the ephrins, were discovered more than three decades ago. Initial studies have largely focused on the role of Eph-ephrin system in regulating morphogenesis and organogenesis during embryonic development^1–3^. Emerging reports have now established their importance in tissue regeneration/remodeling, inflammation, neurological diseases, cancer progression, and angiogenesis during adult life^4–6^. However, targeting the Eph-ephrin proteins in cancer remains a therapeutic challenge. Our research group and others have demonstrated that inhibiting EphB4-ephrinB2 interaction with a soluble protein or a plasmid-based derivative of peptide yields tumor growth retardation, but the magnitude of response is minimal and transient, at best^7–9^. Several key factors can be considered^1^. The EphB4 receptor and its ephrinB2 ligand are present at varying levels in the cancer cells and in the tumor microenvironment (TME), including the vasculature^2^. Systemic targeting of this receptor-ligand pair using an antagonist can have a multitude of effects in these cellular and non-cellular compartments^3^. Both EphB4 and ephrinB2 can signal and the complex EphB4-ephrinB2 signaling can manifest into either pro-tumorigenic or anti-tumorigenic effects as reported in other tumor models^10,11^. However, in the context of head and neck squamous cell carcinoma (HNSCC), no evidence is available of independently dissecting receptor-mediated forward or ligand-mediated reverse signaling. Therefore, carefully examining the directionality of EphB4-ephrinB2 signaling on cancer cells and on vascular endothelial cells is of paramount importance to differentiate between drivers of tumorigenic effects in the cancer cells and/or the TME.

In this work, we find differential compartmental expression of the EphB4 receptor and its ligand, ephrinB2, in the cancer cells and within the TME of HNSCC. We further explore the functional relevance and signaling interactions of this EphB4-ephrinB2 compartmental expression using a combination of CRISPR-Cas9 gene knockouts and dominant-negative constructs in vitro, ex ovo, and in conditional knockout murine models. We observe that EphB4 knockdown on the cancer cells accelerates tumor growth and promotes angiogenesis. Loss of EphB4 on the cancer cells elicits a robust compensatory effect mediated by EphA4 and results in an influx of immunosuppressive regulatory T cells (Tregs). An increase in tumor growth mediated by EphB4 downregulation on cancer cells, however, can be overcome either by genetic ablation of Tregs or by pharmacologic inhibitors of tyrosine kinase receptors. Of note, the intracellular signaling domain of EphB4 (or forward signaling) plays a central role in mediating the tumor-promoting effects. Targeting ephrinB2, on the other hand, shows maximal tumor growth inhibition when lost on both the cancer cell and the vasculature. Transcriptional analysis and functional assays demonstrate that the observed phenotype appears to be mediated by changes in vascular dynamics and immune remodeling within the tumor milieu. Patient data from a clinical trial and from the TCGA show that high EPHB4-low EFNB2 corresponds to better response rates and survival outcomes in HNSCC patients. Overall, our findings underscore the signaling complexity mediated by EphB4 and ephrinB2 in the HNSCC cells and its TME. Our dissection of the mechanisms by which their loss of the cancer cells and on the vascular compartment differentially impact tumor growth may have implications for future drug design.

## Results

### Expression of EphB4 and ephrinB2 varies within the HNSCC TME and across different tumor subtypes

Data on the implications of EphB4 and ephrinB2 expression and cancer progression have been conflicting depending on the disease site. Given their known role in vascular development, several studies have shown that inhibition of EphB4-ephrinB2 signaling arrests endothelial cell migration and vessel formation and branching, suggesting it could be used as a part of anti-angiogenic therapy^12–16^. In other tumor models, however, disruption of EphB4-ephrinB2 signaling caused no significant changes in tumor vasculature but instead demonstrated a strong mitogenic effect and accelerated tumor growth^17–19^. The outcome of EphB4-ephrinB2 interactions appears to be cell type-dependent and microenvironment-dependent.

**Fig. 1:**
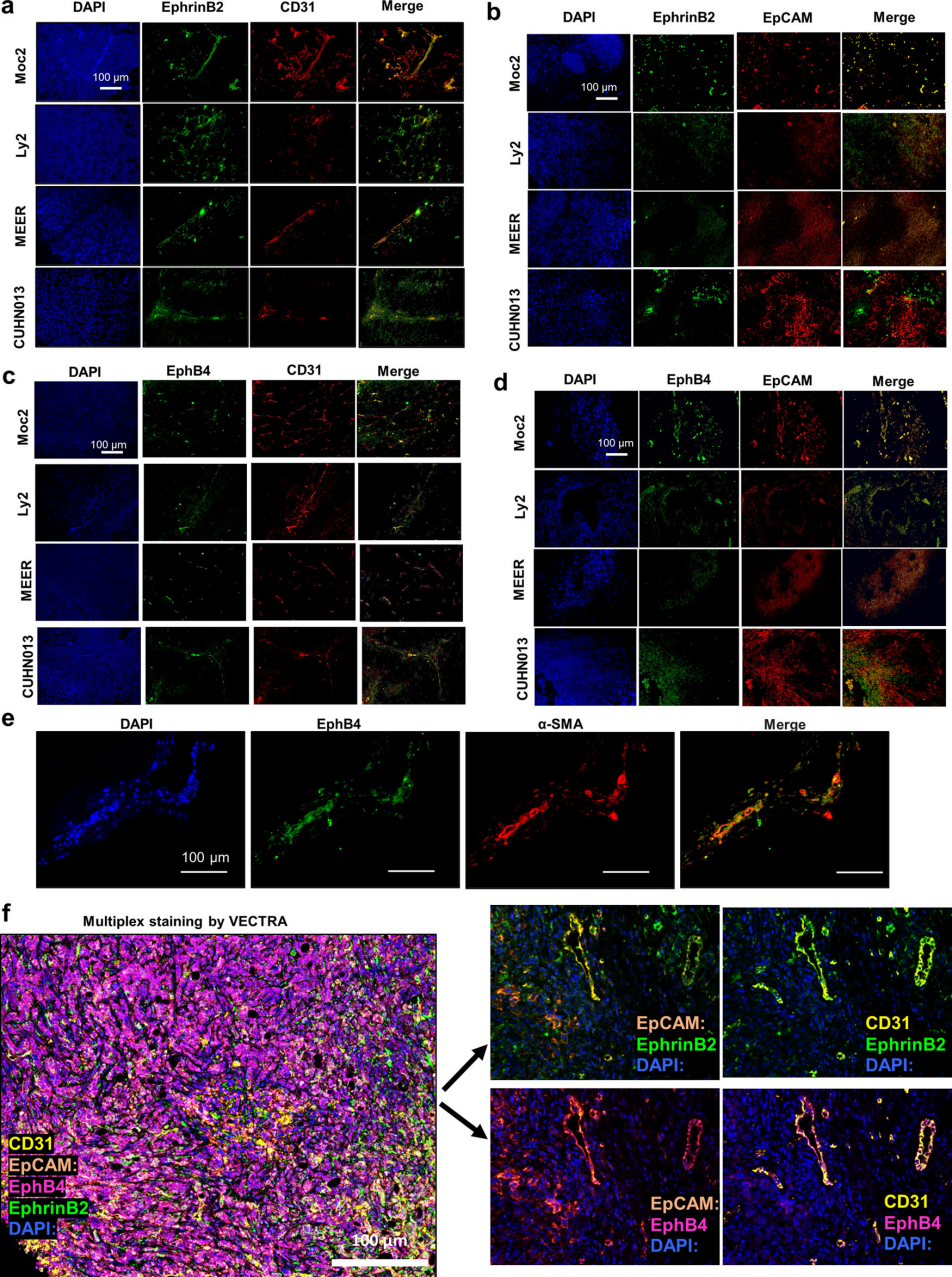
Variable expression of EphB4 and ephrinB2 is found on HNSCC tumor cells and within the TME across different tumor models. Representative HNSCC tumor sections stained with anti-ephrinB2 and CD31 antibodies (**a**) or anti-ephrinB2 and EpCAM antibodies (**b**) confirm the variable expression of ephrinB2 in both epithelial and CD31-expressing vascular endothelial cells. **c** EphB4 is also evident in CD31+ endothelial cells and on **d** EpCAM+ epithelial cells in different tumor models. **e** Dual immunofluorescence staining performed on Moc2 tumors show co-localization of EphB4 on alpha-SMA expressing fibroblasts. **f** Multiplex staining by VECTRA analysis validates the immunofluorescence data in Moc2 tumor. A representative composite image along with dual-color staining is shown. Total magnification: x200.

In HNSCC, response to EphB4-ephrinB2 inhibitors has been modest at best, and tumor growth delay is only noted when combined with radiation therapy^7,8^. We hypothesized that similar to other disease models, this could be due to the dichotomous and opposing net effect of EphB4-ephrinB2 interactions driven by cell type-dependent interactions. To test this hypothesis, we first examined the expression of ephrinB2 and EphB4 in a compartment-specific manner in different murine (Moc2, Ly2, and MEER) and patient-derived xenograft (CUHN013) HNSCC models, with a particular focus on cancer cells and vascular endothelial cells. Our immunofluorescence staining data showed that across all models examined, ephrinB2 expression was present on CD31-positive endothelial cells (Fig. 1a). Variable levels of cancer cell expression, however, were also detected for ephrinB2, with the highest level in the Moc2 tumor model (Fig. 1b). The lowest levels were noted in the Ly2, where ephrinB2 expression within the TME appeared to be mostly vascular (Fig. 1a, b). Similarly, in the CUHN013 tumor model, the expression of ephrinB2 appeared to be predominantly vascular, but moderate levels were also present on EpCam positive tumor epithelial cells (Fig. 1a, b).

In contrast to ephrinB2, EphB4 was ubiquitously expressed in the cancer cells across the different tumor models (Fig. 1d). EphB4 expression was also present on endothelial blood vessels (Fig. 1c) and fibroblasts (Fig. 1e). These data were further validated in Moc2 tumors using multiplex VECTRA imaging (Fig. 1f and Supplementary Fig. 1). To confirm the relevance of these data in human samples, we examined single-cell RNA-seq analysis of a public database of 6000 cells from 18 oral cavities HNSCC patients^20^. EphrinB2 was present on endothelial cells and for most patients in the tumor epithelial cells, whereas EphB4 was present, albeit with variable frequency, on tumor epithelial cells of all the patients, on endothelial cells, and on fibroblasts (Supplementary Fig. 2). These data were further validated by immunohistochemical analysis in the head and neck cancer patients that have undergone chemoRT (Supplementary Fig. 3). Overall, we find ephrinB2 expression to be predominantly vascular, except for the Moc2 model where high levels were also noted on the cancer cells. EphB4, on the other hand, was predominantly expressed in the cancer cells.

### Loss of ephrinB2 on both tumor cells and vascular endothelial cells results in a significant decline in tumor growth in vivo while EphB4 loss on cancer cells accelerates tumor growth

**Fig. 2:**
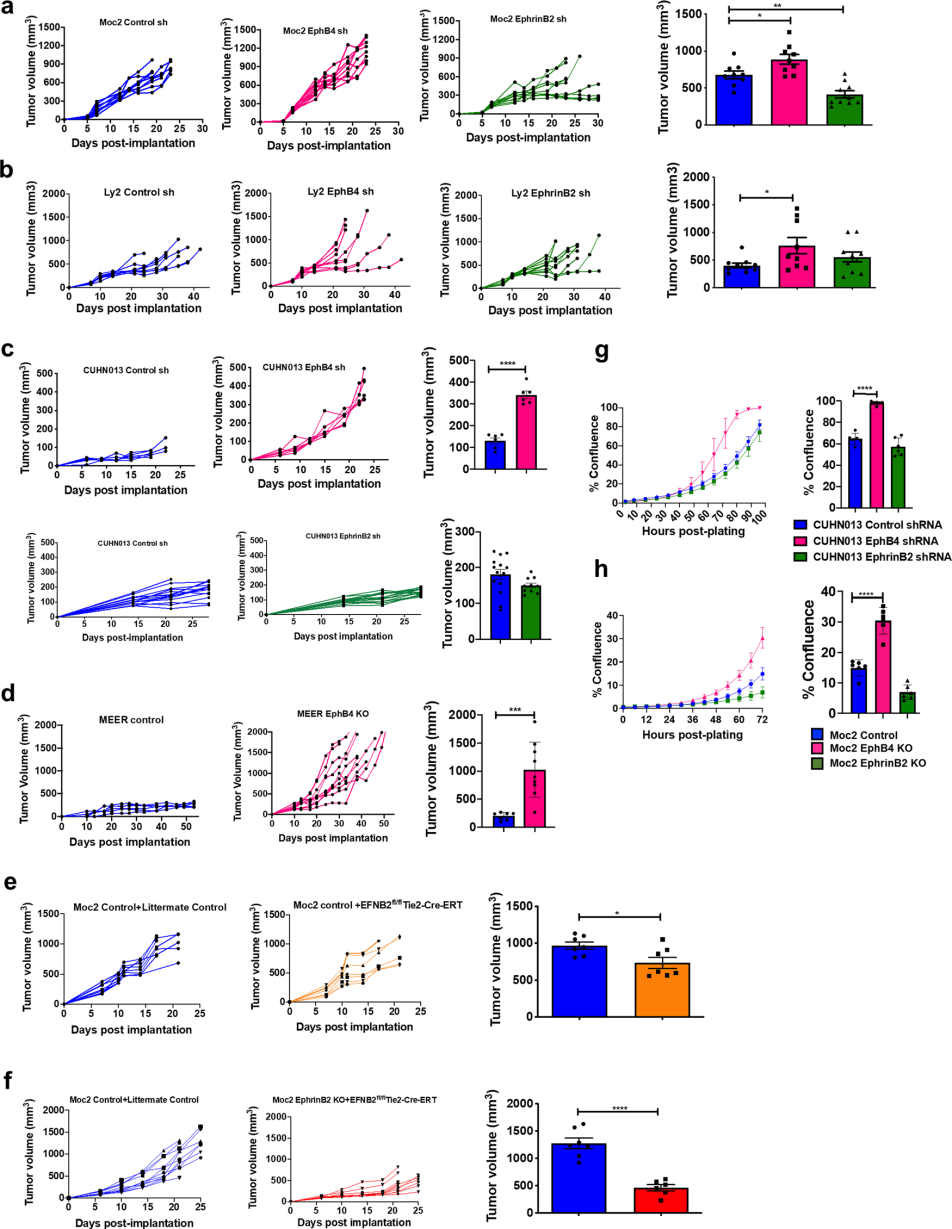
Loss of ephrinB2 in both the cancer cells and the vasculature inhibits tumor growth while knockdown of EphB4 promotes tumor growth progression in orthotopic and xenograft models of HNSCC. Accelerated tumor growth is observed following knockdown of EphB4 on cancer cells in (**a**) Moc2 [*n* = 9 (control sh); *n* = 10 (EphrinB2 sh, EphB4 sh], **b** Ly2 (*n* = 10/group), **c** CUHN013 [upper panel: *n* = 6 (control sh); *n* = 6 (EphB4 sh), lower panel: *n* = 16/group] and **d** MEER [*n* = 7 (control); *n* = 10 (EphB4 KO)] models. Tumor volume data are shown in the form of spaghetti plots to present tumor growth of individual mice for the respective groups in a time-dependent manner. The groups in **a**–**d** are annotated based on the tumor cells implanted in the C57BL/6 mice. Dot plots are also shown to present tumor volumes on day 19 (Moc2), day 24 (Ly2), day 22 (CUHN013), and day 33 (MEER) post tumor implantation. **a**–**c** EphrinB2 knockdown in the Moc2 tumors reduced tumor growth, whereas, in Ly2 or CUHN013 tumors, ephrinB2 manipulation on cancer cells failed to show a similar effect. **e** Conditional deletion of ephrinB2 on the vasculature results in a modest decrease in Moc2 tumor growth in EFNB2^fl/fl^Tie2-Cre-ERT (*n* = 7) mice compared to the controls (*n* = 8) as shown by temporal growth curves and by dot plots at day 17 post-tumor implantation. The group annotation refers to the Moc2 control tumors implanted in either littermate controls (left) or EFNB2^fl/fl^Tie2-Cre-ERT mice (right). **f** Knockout of ephrinB2 in both the tumor cells and the vasculature [Moc2 ephrinB2 KO + EFNB2^fl/fl^Tie2-Cre-ERT mice (*n* = 10)] results in a maximal decline in tumor growth compared to the control counterparts (*n* = 11) in vivo in a time-dependent manner. Dot plots are shown on day 25 post-implantation. The groups correspond to the Moc2 control tumors implanted in littermate controls (left) or Moc2 ephrinB2 KO tumors implanted in EFNB2^fl/fl^Tie2-Cre-ERT mice (right). Data are shown as mean ± SEM. The color key for groups shown in histogram plots (**a**–**f**) is the same as depicted in the respective spaghetti plots. (**g**, **h**) IncuCyte in vitro assay shows an effect on tumor cell growth following downregulation of EphB4 and ephrinB2 in CUHN013 (**g**) and Moc2 (**h**) cell lines (*n* = 6/group). The experiments were replicated two times. Statistical significance was analyzed by performing two-sided Student’s*t*-test or ANOVA. The Dunnett post-hoc test was used after ANOVA where multiple experimental groups were involved. *p*-values are indicated for figures **a** *p = 0.029, **b** *p = 0.04, **c** ****p ≤ 0.0001, **d** ***p = 0.0006, **e** *p = 0.023, **f**–**h** ****p ≤ 0.0001.

Based on the immunofluorescence data, we dissected the functional significance of EphB4 and ephrinB2 expression by generating cancer cell-specific knockdowns of EphB4 or ephrinB2 using shRNA or CRISPR approach in both HPV-unrelated (Moc2, Ly2, and CUHN013) and E6-E7-driven HPV-like (MEER) HNSCC lines (Supplementary Fig. 4). The schematic of the EphB4 and ephrinB2 shRNA/CRISPR KO constructs is represented in Supplementary Fig. 5a. When ephrinB2 was knocked down on tumor cells and implanted orthotopically, a modest decrease (680.5 mm^3^ in control to 415.9 mm^3^ in EphrinB2 sh) in tumor growth was noted only in the Moc2 tumor model, where we have established higher levels of expression on the cancer cells (Fig. 2a and Supplementary Fig. 6a). In contrast, consistent with the expression data, no significant difference in tumor growth was observed with ephrinB2 knockdown on the cancer cell in either the Ly2 or the CUHN013 models (Fig. 2b, cand Supplementary Fig. 6b, c).

To investigate the role of vascular ephrinB2 and its contribution to HNSCC tumor progression, we used a genetically engineered EFNB2^fl/fl^Tie2-Cre-ERT mouse model in which Efnb2 is conditionally knocked out from the endothelial vasculature (Supplementary Fig. 5b). The knockout of ephrinB2 on Tie2-expressing cells was confirmed by dual immunofluorescence staining using anti-ephrinB2 and anti-Tie2 antibodies (Supplementary Fig. 7). Our data showed that loss of vascular ephrinB2 resulted in a modest, but significant (*p* < 0.05), decrease in mean tumor volume compared to the control group at day 17 (Fig. 2e and Supplementary Fig. 6e). This suggested that while vascular ephrinB2 is playing a potential regulatory role in promoting tumor growth, cancer cell ephrinB2 expression might be compensating for the vascular knockdown. We, therefore, adopted a more robust approach where cancer cells with ephrinB2 knockout were implanted in the EFNB2^fl/fl^Tie2-Cre-ERT mice (herein referred to as the ephrinB2 double knockout group). We observed that such a double knockout resulted in a significant decline in tumor growth (Fig. 2f and Supplementary Fig. 6f). The mean tumor volume in the experimental mice was 2.77-fold lower (*p* < 0.0001) compared to the control mice at day 18 post-implantation (Fig. 2f), suggesting that the loss of ephrinB2 on both tumor cells and vascular endothelial cells is necessary to achieve a maximal decline in tumor growth.

As EphB4 is a key binding partner to ephrinB2 and given its high levels of expression on the cancer cells, we next investigated the effect of EphB4 loss-of-function in different orthotopic syngeneic models of HNSCC. Regardless of the tumor model, a significant increase in tumor volume was observed when EphB4 was either knocked down or completely knocked out on cancer cells (Fig. 2a–d and Supplementary Fig. 6a–d). Specifically, shRNA knockdown of EphB4 resulted in a significant increase in median tumor volume in Moc2 (680.5 mm^3^ in control to 888.7 mm^3^ in EphB4 sh) and Ly2 tumor-bearing mice (402.5 mm^3^ in control to 762 mm^3^ in EphB4 sh), as shown in Fig. 2a, b. In the MEER tumor model, complete knockout of EphB4 on cancer cells similarly resulted in a significant enhancement (*p* = 0.0006) in mean tumor volume compared to the control cohort (198.8 mm^3^ in control to 1024 mm^3^ in EphB4 KO) (Fig. 2d and Supplementary Fig. 6d). Similar to the syngeneic models, CUHN013 tumor-implanted mice showed a significant increase in tumor growth following shRNA-mediated silencing of EphB4 receptor on cancer cells (Fig. 2c and Supplementary Fig. 6c).

The knockdown of EphB4 and ephrinB2 on the cancer cell was confirmed by co-immunofluorescence staining between EpCAM and EphB4 or ephirnB2 (Supplementary Fig. 8). We also analyzed the effects of EphB4 and ephrinB2 knockdown in an in vitro real-time IncuCyte assay to determine if the differences in growth are due to a direct effect on the tumor cell’s ability to grow or perhaps due to the signaling triggered within the TME. EphB4 knockdown on CUHN013 cells resulted in a significant increase in cell growth compared to both the control and ephrinB2 knockdown tumor cells at varying time points (Fig. 2g). Similar to CUHN013 cells, our data showed a significant increase in cancer cell growth in the Moc2 EphB4 KO group compared to the control group (Fig. 2h). The in vitro data correlated well with the in vivo immunofluorescence analysis showing a significant increase in the percentage of PCNA+ cells in the Moc2 EphB4 KD group vs control (Supplementary Fig. 9a). Interestingly, this was a common trend observed in all the other tumor models (Ly2, MEER, and CUHN013) analyzed in our study (Supplementary Fig. 9b–d) suggesting a tumor cell-intrinsic increase in cell growth due to the loss of EphB4 on cancer cell.

### Combined loss of ephrinB2 on HNSCC cells and on vascular endothelial cells leads to tumor growth retardation by normalization of tumor vasculature

**Fig. 3:**
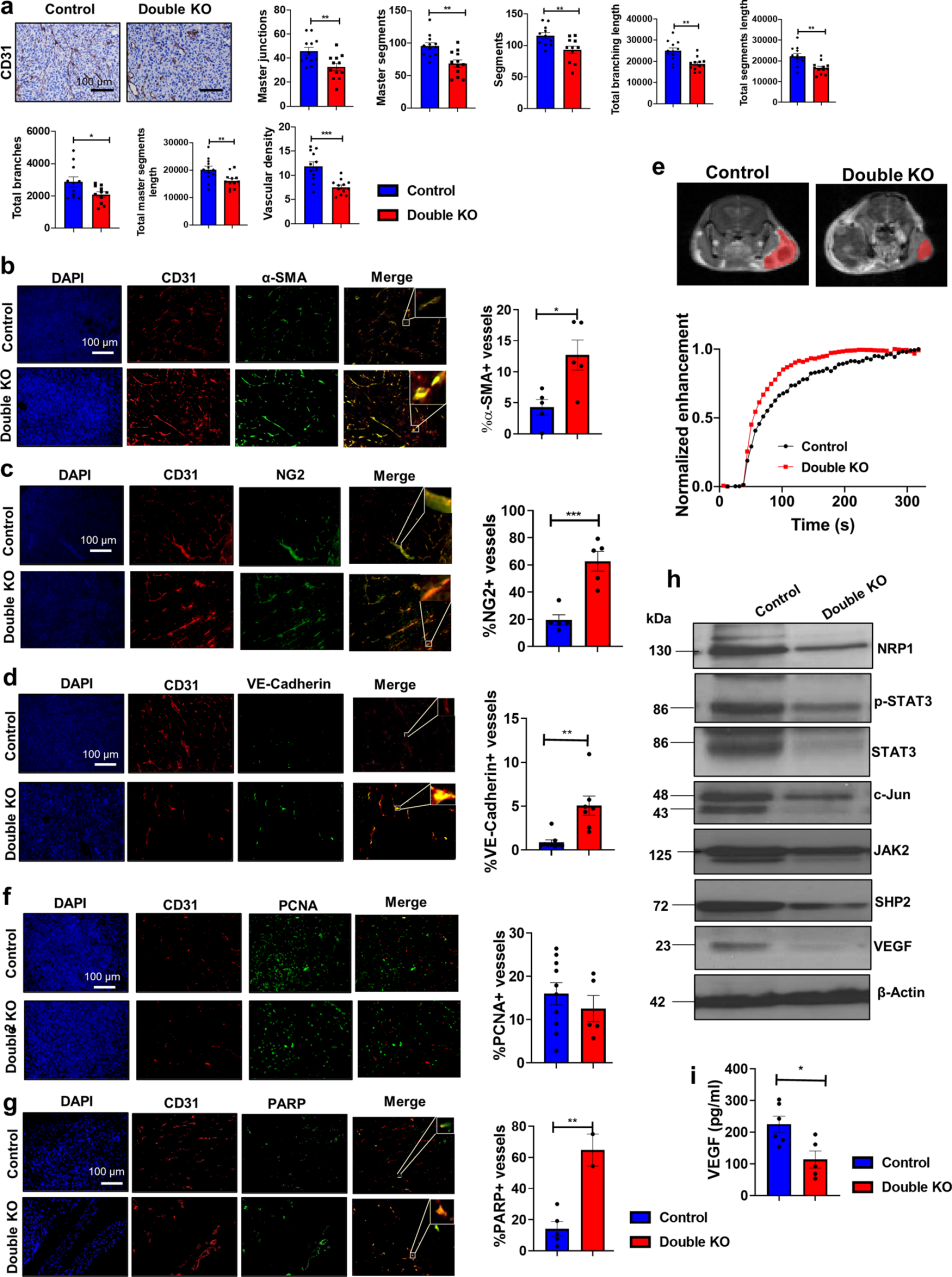
EphrinB2 knockout HNSCC cells implanted in EFNB2^fl/fl^Tie2-Cre-ERT mice result in tumor growth retardation by normalization of tumor vasculature. **a** CD31 immunostaining is performed on EFNB2^fl/fl^Tie2-Cre-ERT (Double KO) tumors and their respective controls followed by quantitative analysis of vascular parameters using ImageJ software. Dual immunofluorescence staining is performed on tumors harvested from these mice to analyze the co-expression of CD31 with alpha-SMA (**b**), NG2 (**c**), VE-cadherin (**d**), PCNA (**f**), and PARP (**g**), showing improved vascular function in tumor-bearing mice following the loss of ephrinB2 on cancer cells and vasculature. The immunostaining in Figures **a**–**d**, **f**, **g** is performed in two sets with following number of captured images: **a** *n* = 12; **b** *n* = 5; **c** *n* = 5; **d** *n* = 7; **f** *n* = 5; **g** *n* = 5 control. Total magnification: x200. **e** DCE-MRI show increased enhancement of contrast agent in a time-dependent manner in representative ephrinB2 KO tumor-bearing EFNB2^fl/fl^Tie2-Cre-ERT mice (Double KO). **h** Decrease in the protein levels of key markers responsible for different aspects of angiogenesis is evident in ephrinB2 double knockout tumor tissues. **i** VEGF ELISA shows decreased levels of circulating VEGF in ephrinB2 double knockout mice (*n* = 5) compared to the controls (*n* = 6). The experiments were performed in two except in (**e**). Color key for histogram plots in Figures **b**–**d**, **f**, **g**, **i**: Blue: Control; Red: Double KO. Data are shown as mean ± SEM. Control mice refers to the littermate controls implanted with Moc2 control KO tumors. Comparison between the control and experimental groups was done using two-sided Student’s *t*-test. *p*-values are indicated for the figures: **a** master junctions **p = 0.005; master segments, total segments length **p = 0.001; segments **p = 0.008; total branches *p = 0.03; total master segments length **p = 0.005; vascular density ***p = 0.0002, **b** *p = 0.01, **c** ***p = 0.0007, **d** **p = 0.001, **g** **p = 0.003, **i** *p = 0.015.

The structural and functional anomalies in tumor vasculature support the development of pro-tumorigenic, immunosuppressive, and therapy-resistant TME^21^. Therefore, using the strategies that can revert the grossly aberrant structure and function of tumor vasculature towards a more normal state (vessel normalization) can have implications on tumor growth^21^. To determine the mechanisms by which the combined knockout of ephrinB2 on the cancer cell and on the vascular endothelial cells inhibits tumor growth, we performed a morphological analysis of tumor vasculature by performing CD31 staining for endothelial cells^22^. We observed that the vessels in the ephrinB2 KO tumor-bearing EFNB2^fl/fl^Tie2-Cre-ERT mice (referred as Double KO, Supplementary Fig. 5c) displayed significantly fewer branches, segments, and master junctions compared to the littermate control group (Fig. 3a). The vessel density in the EFNB2^fl/fl^Tie2-Cre-ERT mice implanted with ephrinB2 knockout tumors was also significantly reduced compared to the control group where ephrinB2 was expressed on vascular endothelial cells suggestive of a decrease in angiogenic phenotype (Fig. 3a).

Next, we analyzed the effect of ephrinB2 loss on both tumor cells and vascular endothelial cells on vessel maturation. The vascular lumen in normal tissue has extensive coverage by vascular smooth muscle cells (VSMCs). Therefore, to detect lumen morphology, we stained the tumors with α-smooth muscle actin (SMA), a VSMC marker. Whereas, vessels in the tumors harvested from the control mice were almost devoid of α-SMA expression, representing an erratic lumen morphological feature, knocking out ephrinB2 from both the cancer cells and vascular endothelial cells restored the α-SMA expression on the vessels as determined by the immunofluorescence analysis (Fig. 3b). Since α-SMA is also expressed on cancer-associated fibroblasts, we further validated our results by using pericyte coverage by neuron-glial antigen 2 (NG2) staining as an indication of vessel maturity. Consistent with α-SMA staining, greater NG2 staining was found on the vessels from the ephrinB2 double knockout mice compared to the control group (Fig. 3c), further suggesting that the vessels in the control group with intact ephrinB2 expression are immature in structure and that loss of ephrinB2 reverses this phenotype. Interestingly, loss of ephrinB2 also reversed the discontinuous VE-Cadherin+ vascular basement membrane observed in the control tumors towards a more continuous phenotype while enhancing VE-Cadherin expression (Fig. 3d). These data suggest that ephrinB2 plays a role in enhancing angiogenesis by disrupting vascular integrity and structural morphology and that its loss on cancer cells and vascular endothelial cells induces vascular normalization in an HNSCC model.

### Vessel normalization following combined loss of ephrinB2 on cancer cells and on vascular endothelial cells affects vascular function by inducing endothelial cell death and by improving vessel perfusion

Structural maturity of the tumor vascular network is known to affect vascular function^23,24^. Based on the observed vessel normalization in the ephrinB2 double knockout mice (double KO, Supplementary Fig. 5c), we assessed the effect of vascular normalization on the functional integrity of tumor vasculature. Dynamic contrast-enhanced magnetic resonance imaging (DCE-MRI) was performed on the control and the ephrinB2 double knockout mice. Representative DCE-MR images with normalized signal intensity curves in Fig. 3e show the dynamic time course of uptake and enhancement of contrast agent, suggesting improved perfusion in the double KO versus the control group.

We also performed dual immunofluorescence staining by using anti-CD31 and anti-PCNA antibodies to determine vascular endothelial cell proliferation. While vessel proliferation between the control tumors and the ephrinB2 double KO tumors remained unchanged (Fig. 3f), we observed a significant increase in apoptosis in the ephrinB2 double KO tumors compared to the controls as shown by the co-localization of anti-CD31 and PARP (Fig. 3g).

Key molecular players implicated in different aspects of angiogenesis were also analyzed in both the control and ephrinB2 double knockout tumors (Fig. 3h). These include: NRP1, a key receptor for the VEGF165 isoform;^25^ Stat3, a multifunctional transcriptional mediator that regulates different facets of angiogenesis including cell proliferation and survival;^26^ c-Jun, a component of activator protein-1 complex that controls endothelial cell survival in different human cancers;^27^ SHP2, a tyrosine phosphatase that mediates endothelial cell proliferation and vessel growth by the PI3K-AKT and ERK1/2 pathways;^28^ and VEGF, a known pro-angiogenic factor^29^. Compared to the control group, our western blot analysis showed that levels of NRP1, STAT3 (both activated and total), c-Jun, SHP2, and VEGF are reduced in the ephrinB2 double knockout tumors (Fig. 3h). A VEGF ELISA assay was also performed on the serum samples in vivo, which confirmed that the circulating VEGF was significantly lower in the ephrinB2 double knockout mice compared to the control group (Fig. 3i). These data suggest that the absence of ephrinB2 signaling on cancer cells and vascular endothelial cells tips the balance towards an anti-angiogenic phenotype, ultimately resulting in tumor growth inhibition.

### EphB4 cancer cell-intrinsic forward signaling acts to suppress tumor growth, independent of stromal EphB4

**Fig. 4:**
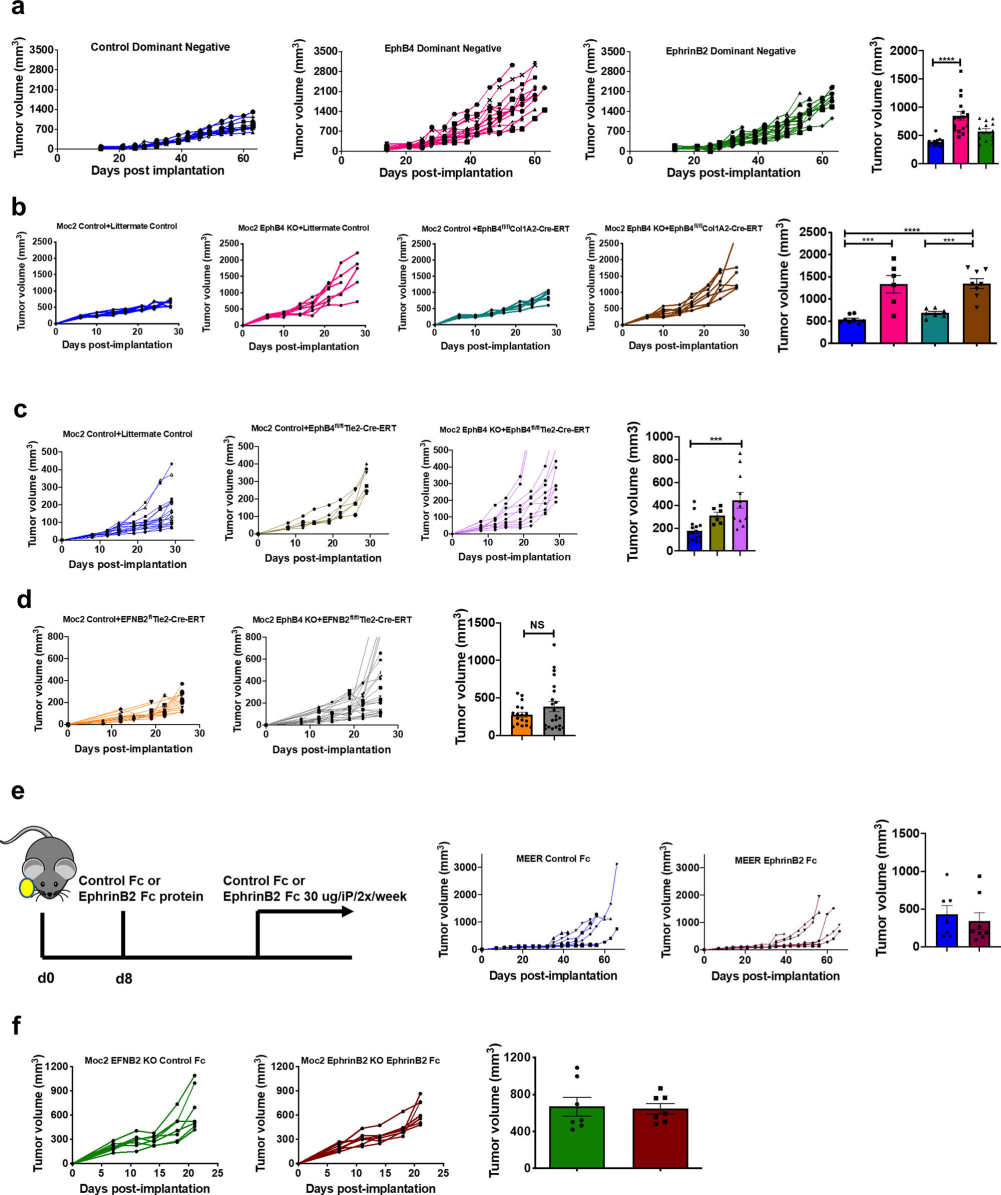
Activating EphB4 on cancer cells in the absence of vascular ephrinB2 fails to reduce tumor growth in different models of HNSCC. **a** EphB4 intracellular signaling in EphB4 dominant-negative constructs significantly enhances tumor growth in a patient-derived xenograft model. CUHN013 HNSCC cells transfected with either control or dominant-negative plasmids of EphB4 and ephrinB2 were implanted in the flank region of nude mice [*n* = 6 (control); *n* = 9 (EphB4 dominant negative); *n* = 10 (ephrinB2 dominant negative), and tumor growth was monitored and shown for individual mice in spaghetti plots in a time-dependent manner. The groups are annotated based on the tumor cells implanted in the respective mice. Dot plots are shown at day 42 post-implantation. Loss of EphB4 in collagen I-expressing cells such as fibroblasts in EphB4^fl/fl^Col1A2-Cre-ERT mice (*n* = 6) (**b**) or in adult vasculature in EphB4^fl/fl^Tie2-Cre-ERT mice (*n* = 8) (**c**) did not significantly impact the tumor growth as compared to the littermate controls (*n* = 8). **d** Implantation of EphB4 KO tumor cells in EFNB2^fl/fl^Tie2-Cre-ERT mice with conditional deletion of ephrinB2 on the vascular endothelial cells failed to achieve tumor growth suppression. The groups in figures **b**–**d** are annotated in the format: “tumor name+mouse strain”. Tumor growth data is also shown in MEER control (*n* = 8) (**e**) and Moc2 ephrinB2 KO (*n* = 7) (**f**) tumor models where systemic administration of ephrinB2-Fc to activate EphB4 receptor failed to achieve tumor growth reduction in EFNB2^fl/fl^Tie2-Cre-ERT mice (*n* = 7). For figures **e**–**f**, groups are annotated based on the tumor cells implanted followed by Fc treatment. With the exception of **b**–**d**, other experiments were replicated two times. Color key for groups shown in histogram plots is the same as depicted in the respective spaghetti plots. Data are shown as mean ± SEM. Statistical significance was analyzed by performing a two-sided Student’s *t*-test or ANOVA. The Tukey’s test was used after ANOVA where multiple experimental groups were involved. *p*-values for the figures are indicated: **a** ****p < 0.0001, **b** blue vs brown bar ****p < 0.0001; blue vs pink bar ***p = 0.0001; teal vs brown bar ***p = 0.0007, **c** ***p = 0.0003.

To better understand the contribution of signaling triggered by EphB4 and ephrinB2 on tumor growth, we generated HNSCC cells expressing dominant-negative constructs of EphB4 and ephrinB2. The schematic of the dominant-negative constructs is presented in Supplementary Fig. 10. This was done by replacing the kinase/cytoplasmic domain of EphB4/ephrinB2 with the EGFP protein instead of eliminating the receptor/ligand in its entirety^15^. The EphB4 dominant-negative cells lack the EphB4 kinase domain, but the receptor can still bind to the ephrinB2 ligand to initiate reverse signaling. The ephrinB2 dominant-negative cells, on the other hand, lack the ephrinB2 cytoplasmic domain but can bind to the EphB4 receptor to initiate forward signaling (Supplementary Fig. 10). When ephrinB2 dominant-negative HNSCC cells were implanted in vivo, we observed no significant change in tumor growth (Fig. 4a and Supplementary Fig. 11a). The EphB4 dominant-negative tumors, on the other hand, showed a significant increase in tumor growth in a time-dependent manner (Fig. 4a and Supplementary Fig. 11a). The mean tumor volume in the EphB4 dominant-negative group was 2.19-fold higher than the control group at day 42 post-implantation. These data suggest that the intracellular domain of EphB4 may promote a tumor-suppressive function. Further substantiating a cancer cell-intrinsic tumor-suppressive effect of the EphB4 intracellular domain, an IncuCyte cell growth assay established that EphB4 dominant-negative cells increase cell growth compared to the control group (Supplementary Fig. 12).

Given that EphB4 is also expressed on stromal cells, such as fibroblasts and vessels (Fig. 1), we next sought to determine whether the accelerated tumor growth observed with inhibition of EphB4 forward signaling on cancer cells induces a feedback loop through increased stromal EphB4 expression as a compensatory mechanism. The Col1A2 expression has been shown to be present on fibroblasts in scRNA seq analysis of public humans (Supplementary Fig. 13) and murine datasets (Supplementary Fig. 14). To test whether stromal EphB4 plays a role as a tumor promoter, we used the genetically engineered mouse model, EphB4^fl/fl^Col1A2-Cre-ERT, with conditional deletion of EphB4 in collagen I-expressing cells such as fibroblasts. Confirmation of EphB4 knockout on Col1A2 expressing cells in EphB4^fl/fl^Col1A2-Cre-ERT mice is shown in Supplementary Fig. 15. We implanted Moc2 control or EphB4 knockout tumors in either littermate controls or EphB4^fl/fl^Col1A2-Cre-ERT mice. Our data showed that EphB4 knockout in the mouse collagen I-expressing cells such as fibroblasts did not mitigate the accelerated tumor growth (Fig. 4b and Supplementary Fig. 11b). Similarly, EphB4 conditional deletion on the vasculature using EphB4^fl/fl^Tie2-Cre-ERT mice failed to retard accelerated tumor growth observed with EphB4 cancer cell knockdown (Fig. 4c and Supplementary Fig. 11c), suggesting that stromal EphB4, particularly on the collagen I-expressing cells and vascular endothelial cells, is not responsible for driving the tumor growth acceleration in the absence of EphB4 on the cancer cells.

### Knockout of vascular endothelial cell ephrinB2 fails to counteract EphB4-mediated increased tumor growth

To further support a cancer cell-intrinsic tumor-suppressive effect of EphB4 that is independent of ephrinB2, we studied whether knockout of endothelial cell ephrinB2 expression can overcome the accelerated tumor growth observed in vivo following the knockout of EphB4 on cancer cells. Our data did not show any significant reduction in tumor growth in the EFNB2^fl/fl^Tie2-Cre-ERT mice implanted with Moc2 EphB4 KO tumors compared to the control tumors in EFNB2^fl/fl^Tie2-Cre-ERT mice (Fig. 4d and Supplementary Fig. 11d). Our findings establish that, following the loss of EphB4 on the cancer cell, targeted deletion of its binding partner, ephrinB2 on vascular endothelial cells alone does not result in any meaningful tumor growth reduction.

Since we observed accelerated tumor growth in both CUHN013 EphB4 knockdown and EphB4 dominant-negative tumors (Figs. 2c, 4a), we investigated whether forward signaling via EphB4’s intracellular domain exerts a tumor-suppressive effect on tumor cell growth. If so, we reasoned, that forced activation of EphB4 should reduce tumor growth. To test this hypothesis, we used a recombinant ephrinB2-Fc protein to activate EphB4 (schematically shown in Supplementary Fig. 16) on the control cells in the MEER tumor model (Fig. 4e) or in the ephrinB2 knock out cancer cells in the Moc2 tumor model (Fig. 4f). Both these tumor types were implanted in the EFNB2^fl/fl^Tie2-Cre-ERT ephrinB2 vascular endothelial knockout mice. Although EphB4 activation was evident with the administration of ephrinB2-Fc (Supplementary Fig. 17), no additional growth inhibitory effects were observed when ephrinB2 is knocked out either on the vasculature alone (Fig. 4e and Supplementary Fig. 11e) or on both the cancer cell and the vasculature (Fig. 4f and Supplementary Fig. 11f). These findings support the concept that, unless ephrinB2 is knocked out, tumor growth retardation will not be observed in the presence of EphB4 activation.

### Inhibition of EphB4 cancer cell-intrinsic forward signaling increases vascular network formation and circulating VEGF, whereas inhibition of ephrinB2 cancer cell signaling has minimal effect

**Fig. 5:**
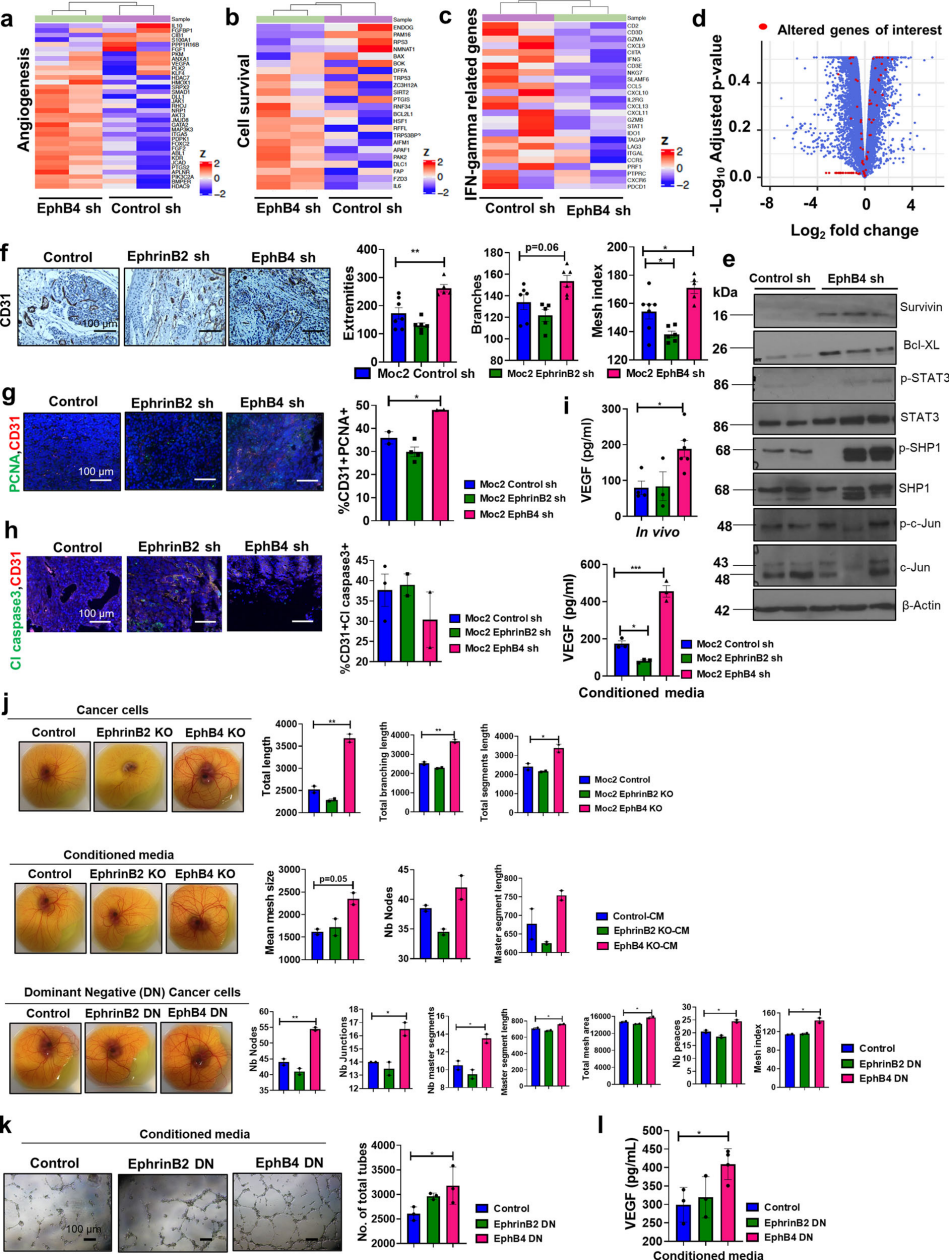
Perturbation of EphB4 signaling on cancer cells enhances vascular network formation and circulating VEGF. EphB4 downregulation in Moc2 tumors induces global changes in the angiogenesis, apoptotic, and IFN-gamma immune gene signatures. Representative heatmaps are shown for the indicated samples. The expression of angiogenesis regulatory genes (**a**) and those that promote cell survival (**b**) is increased in the Moc2 EphB4 shRNA (sample ids# C1M2, C2M2) tumors compared to the control shRNA (sample ids# C2M1, C2M3) tumors, whereas IFN-gamma related Ayers inflammatory gene signature (**c**) is decreased in the Moc2 tumors with EphB4 knockdown (sample ids# C2M2, C1M1) on cancer cells compared to the control shRNA (sample ids# C2M1, C2M3) groups. Key genes in these pathways are represented in the volcano plot as red dots (**d**), with further validation by western blot analysis (**e**). **f** CD31 immunostaining in Moc2 tumors confirms an increased vascular effect following downregulation of EphB4 in Moc2 tumors in duplicate sets. Knockdown of EphB4 on cancer cells enhances endothelial cell proliferation (**g**) without an effect on endothelial cell survival (**h**) in Moc2 tumors in duplicate sets. Total magnification: x200. **i** Plasma samples of conditioned media collected from Moc2 EphB4 sh or KO cells [*n* = 3 (lower panel); *n* = 6 (upper panel)] was subjected to ELISA to detect the circulating levels of VEGF. **j** Chick chorioallantoic membrane (CAM) assay is performed in duplicate sets on Moc2 cancer cells, conditioned media, and CUHN013 dominant-negative (DN) cells to validate the vascular effects of modulating EphB4/ephrinB2 on cancer cells. **k** EphB4 dominant-negative tumor cells mediate a strong pro-angiogenic effect in cultured endothelial cells compared to the control cells (*n* = 3). Tubule formation ability of HUVECs in the presence of conditioned media collected from CUHN013 dominant-negative cells is determined at 6 h. Total magnification x40. (**l**) Conditioned media from CUHN013 EphB4 dominant-negative cancer cells (*n* = 4) show an upregulation in the VEGF ELISA as determined by an ELISA assay. With the exception of a-d (RNA-Seq), other experiments were replicated in two sets. Data are shown as mean ± SEM or mean ± SD. Statistical significance was analyzed by performing ANOVA. The Dunnett post-hoc test was used after ANOVA where multiple experimental groups were involved. *p*-values are indicated for figures: **f** **p = 0.002; *p = 0.02, **g** *p = 0.04, **i** VEGF in vivo *p = 0.021; VEGF conditioned media *p = 0.03, ***p = 0.0001, **j** cancer cells (total length) **p = 0.002; (total branching length) **p = 0.0022; total segments length *p = 0.03, DN cancer cells (nodes) **p = 0.005; (junctions, master segments) *p = 0.03; (master segment lengths, total mesh area) *p = 0.02; (Nb peaces and mesh index) *p = 0.01; **k** *p = 0.035, **l** *p = 0.044.

Given that the accelerated tumor growth observed with cancer cell knockdown of EphB4 was independent of stromal EphB4, we performed RNA-sequencing on Moc2 EphB4 knockdown tumors and control tumors to define the underlying cancer cell-intrinsic mechanisms (Fig. 5a–d). Our RNA-sequencing data revealed global changes in gene signatures affecting angiogenesis (Fig. 5a and Supplementary Data 1) and cell survival pathways (Fig. 5b and Supplementary Data 2). The genes found to be altered are involved in vascular sprouting and density (DLL1), vascular development and permeability (ITGA5, KDR/VEGFR2) (Fig. 5a), cell survival (BCL2L1, RNF34, and FAP), and cell death (ENDOG, BAX) (Fig. 5b). Key targets were validated by western blot analysis in whole tumor lysates. We observed an increase in pro-survival proteins such as survivin, Bcl-XL, and p-STAT3 (Fig. 5e), as well as the pro-angiogenic proteins c-Jun (Fig. 5e), and VEGF (Supplementary Fig. 18a)^30–32^ in the EphB4 knockdown tumors. In addition to tumor tissues, we also subjected Moc2 EphB4 KO and control cell lines to RNA-seq analysis and observed similar changes in the genes involved in the VEGF signaling pathway including VEGFA, DLL1, NRP2 (Supplementary Fig. 18b).

Since the RNA-sequencing data suggested that EphB4 knockdown on cancer cells also affected angiogenic markers (Fig. 5a), we determined the effects of EphB4 cancer cell knockdown on the vascular TME by CD31 immunohistochemical (IHC) staining. In Moc2 tumors with EphB4 knockdown, we observed a significant enhancement in vascular network formation as indicated by an increased number of branches, extremities, and the mesh index compared to the control group (Fig. 5f). Significant increase in endothelial cell proliferation, as suggested by dual CD31^+^/PCNA^+^ staining, was also observed (Fig. 5g), without any notable differences in endothelial cell death, as evaluated by co-immunostaining with cleaved caspase 3 and CD31 (Fig. 5h). ELISA analysis of serum/plasma VEGF levels also showed a significant increase in circulating VEGF in EphB4 knockdown mice compared to the control group (Fig. 5i), suggesting a paracrine signaling mechanism mediating the observed increase in vascular network formation.

To distinguish between direct juxtacrine cell-cell contact-mediated mechanisms and secreted chemokines acting in a paracrine fashion to affect the vascular formation, we utilized a chick chorioallantoic membrane (CAM) model ex ovo as well as an in vitro tubule formation assay. For juxtracrine-mediated signaling in CAM assays, we utilized EphB4 knockdown or dominant-negative EphB4 and their corresponding control cells. For paracrine signaling, conditioned media from the respective groups was added either in an ex ovo CAM assay or in vitro tubule formation assay. Loss of EphB4 forward signaling, either via complete knockdown or dominant-negative EphB4, led to increased vascular network formation compared to the control group as evident by images captured on embryonic day 11 (ED11) (Fig. 5j). Similarly, the addition of conditioned media from either EphB4 knock out cancer cells or dominant-negative EphB4 transfected cells increased the vascular network formation (Fig. 5j, k). Addition of both cancer cells (with complete gene knockout or dominant negatives) or conditioned media resulted in changes in different aspects of vascular growth, such as total vessel length, total branching length, mean mesh size, and the number of nodes compared to the control counterparts, albeit the magnitude of the effect was different for each of these parameters. A significant increase in VEGF levels was detected in the conditioned media of both EphB4 knockout cells (Fig. 5i) and EphB4 dominant-negative cancer cells (Fig. 5l) compared to their respective controls.

In contrast to EphB4 knockdown, knockdown of ephrinB2 reverse signaling on the cancer cell alone demonstrated a small, non-significant decrease in vascular formation (Fig. 5f) and had no effect on endothelial cell proliferation or cell death (Fig. 5g, h) or VEGF levels in vivo (Fig. 5i). When cancer cells with ephrinB2 KO were incubated in the ex ovo CAM assay, small changes in the vascular network in ephrinB2 KO group were observed (Fig. 5j) along with decreased VEGF levels (Fig. 5i). These data suggested that in vivo, the presence of ephrinB2 on vascular endothelial cells compensate for the loss of ephrinB2 on cancer cells. Therefore, when ephrinB2 expression is lost on these two compartments, it manifests in the form of decreased vascular dynamics and reduced VEGF levels as evident in the ephrinB2 double knockdown group (Fig. 3a, i). The ephrinB2 dominant-negative cells also failed to show any significant difference in vascular formation and VEGF levels (Fig. 5k, l). Overall, these data suggest that EphB4 suppresses VEGF production within the TME, an effect mediated via its intracellular domain.

### EphA4 signaling is hyperactivated following loss of EphB4 on the cancer cells and treatment with tyrosine kinase inhibitors reverses EphB4-mediated tumor promoting effect in vivo

**Fig. 6:**
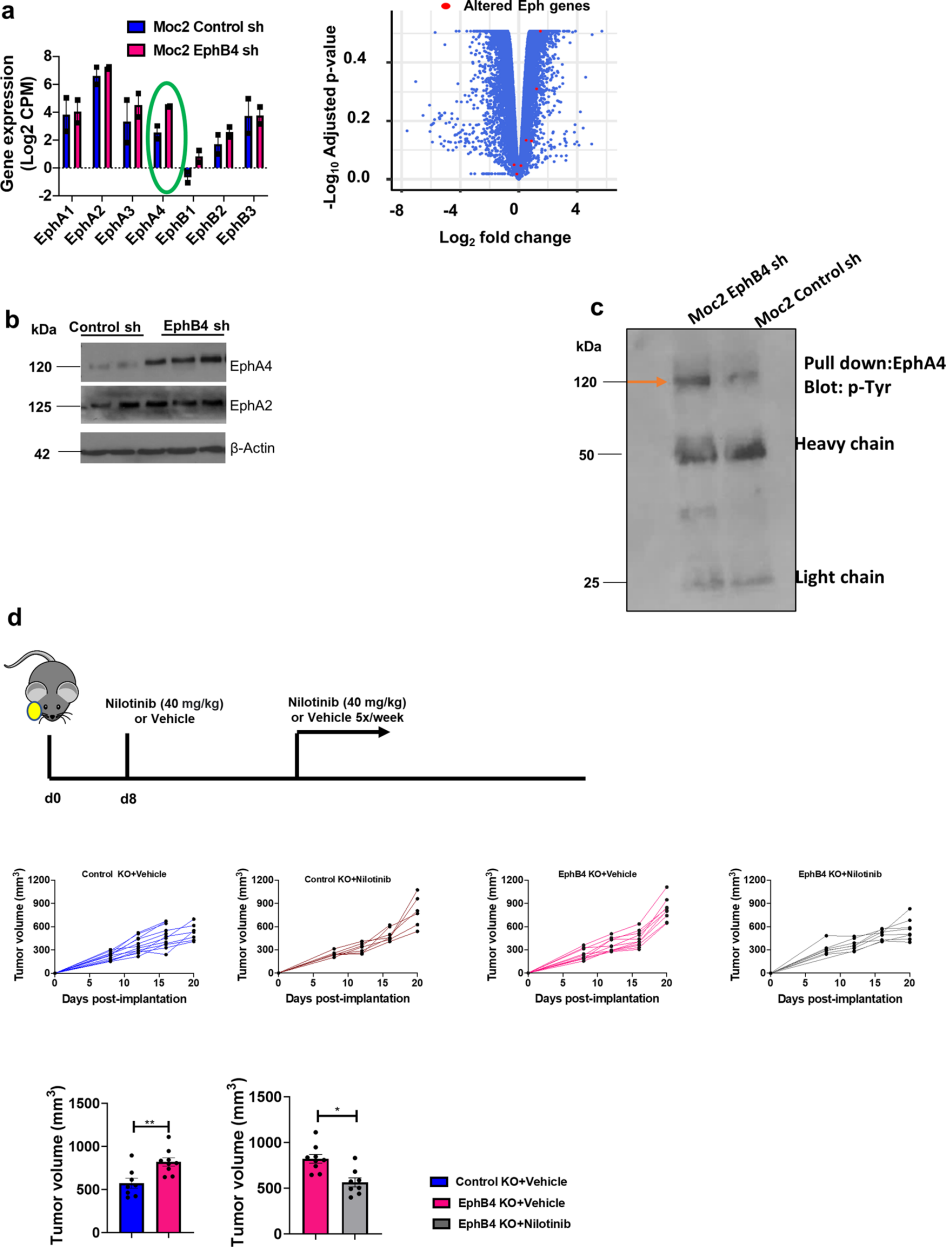
Total and phospho-EphA4 levels are elevated following the loss of EphB4 on cancer cells and targeting EphA4 by broad-activity tyrosine kinase inhibitors reverses the accelerated tumor growth in EphB4 KO tumor-bearing mice. **a** mRNA seq analysis show alterations in the gene expression of different Eph receptors following EphB4 knockdown in Moc2 tumors (*n* = 2). Alteration in Eph genes following EphB4 loss on tumor cells is also represented in the form of volcano plot. **b** Western blot analysis shows an increase in EphA4 levels following knockdown of EphB4 in Moc2 cancer cells, while EphA2 levels remain unchanged. **c** Immunoprecipitation analysis was performed to detect the phosphorylated levels of EphA4 in control vs EphB4 shRNA tumors. EphB4 knockout tumor cells were implanted in the buccal region of mice [*n* = 12 (blue); *n* = 7 (red); *n* = 11 (pink); *n* = 7 (blue)] followed by treatment with Nilotinib (**d**) tyrosine kinase inhibitor once the tumors reached a volume of ~150 mm^3^. The groups are annotated in the format: “tumor name+treatment”. Histogram plot shows significant decrease in inhibitor-treated versus control group at day 20 post-tumor implantation. The experiments were performed once with their own biological replicates. Data are shown as mean ± SEM. Statistical significance was analyzed by performing two-sided Student’s *t*-test. *p*-value: **p = 0.004, *p = 0.025.

We delved into the RNA-sequencing analysis to further explore the overall Eph profile and determine what compensatory mechanisms might play a role upon the loss of EphB4 in HNSCC tumors. We observed an upregulation of both *EPHA2* and *EPHA4* transcripts following the loss of EphB4 on HNSCC cells (Fig. 6a), which was further validated by western blot analysis (Fig. 6b). We also found increased levels of phosphorylated EphA4 in the EphB4 knockdown tumors versus the controls in an immunoprecipitation assay (Fig. 6c).

Given the parallel increase in EphA4 protein levels (Fig. 6b) and its phosphorylation status (Fig. 6c), we questioned whether hyperactivation of EphA4 plays a dominant role in stimulating tumor growth in the absence of cancer cell EphB4 and whether we can reverse it by using the pharmacological intervention. To reverse the tumor growth mediated by EphA4, we used a multi-target tyrosine kinase inhibitor, Dasatinib, in the EphB4 knockout tumor-bearing mice. We observed that Moc2 tumor cells lacking EphB4 treated with Dasatinib had half the mean tumor volume of the vehicle-treated group (Supplementary Fig. 19). These results were further confirmed using an alternate EphA4 kinase inhibitor, Nilotinib (Fig. 6d and Supplementary Fig. 20). In the control group where EphB4 is intact on the cancer cells, treatment with Nilotinib did not result in tumor growth reduction (Fig. 6d and Supplementary Fig. 20). However, when EphB4 knock out tumors in mice were treated with EphA4 inhibitor, a significant tumor growth reduction was observed (Fig. 6d and Supplementary Fig. 20), suggesting that EphA4 plays a compensatory role to enhance tumor growth in vivo following the loss of cancer cell EphB4.

### EphB4 knockdown on tumor cells increases infiltration of immunosuppressive population of Tregs, and enhances apoptosis of CD8 T cells

Another finding that emerged from the RNA sequencing analysis of EphB4 knockdown tumors was a notable decrease in the IFN-gamma related Ayer’s inflammatory gene signature^33^ (Fig. 5c and Supplementary Data 3). This 28-gene set expanded immune gene signature comprised of genes associated with T cell markers involved in T cell development and signal transduction (CD3D, CD3E, CD2, IL2RG), antigen presentation (CIITA), NK cell activity (NKG7), cytotoxic activity (e.g., granzyme A/B, PRF1), cytokines or chemokines for initiation of inflammation (CXCR6, CXCL9, CCL5, and CCR5), additional immunomodulatory or immunosuppressive factors (LAG3, IDO1), and immune cell activation (SLAMF6) (Fig. 5c). We also observed increased p-SHP1 levels on western blotting following cancer cell EphB4 knockdown compared to the control tumors (Fig. 5e). p-SHP1 has been shown to be involved in T cell activation and signaling^34^.

**Fig. 7:**
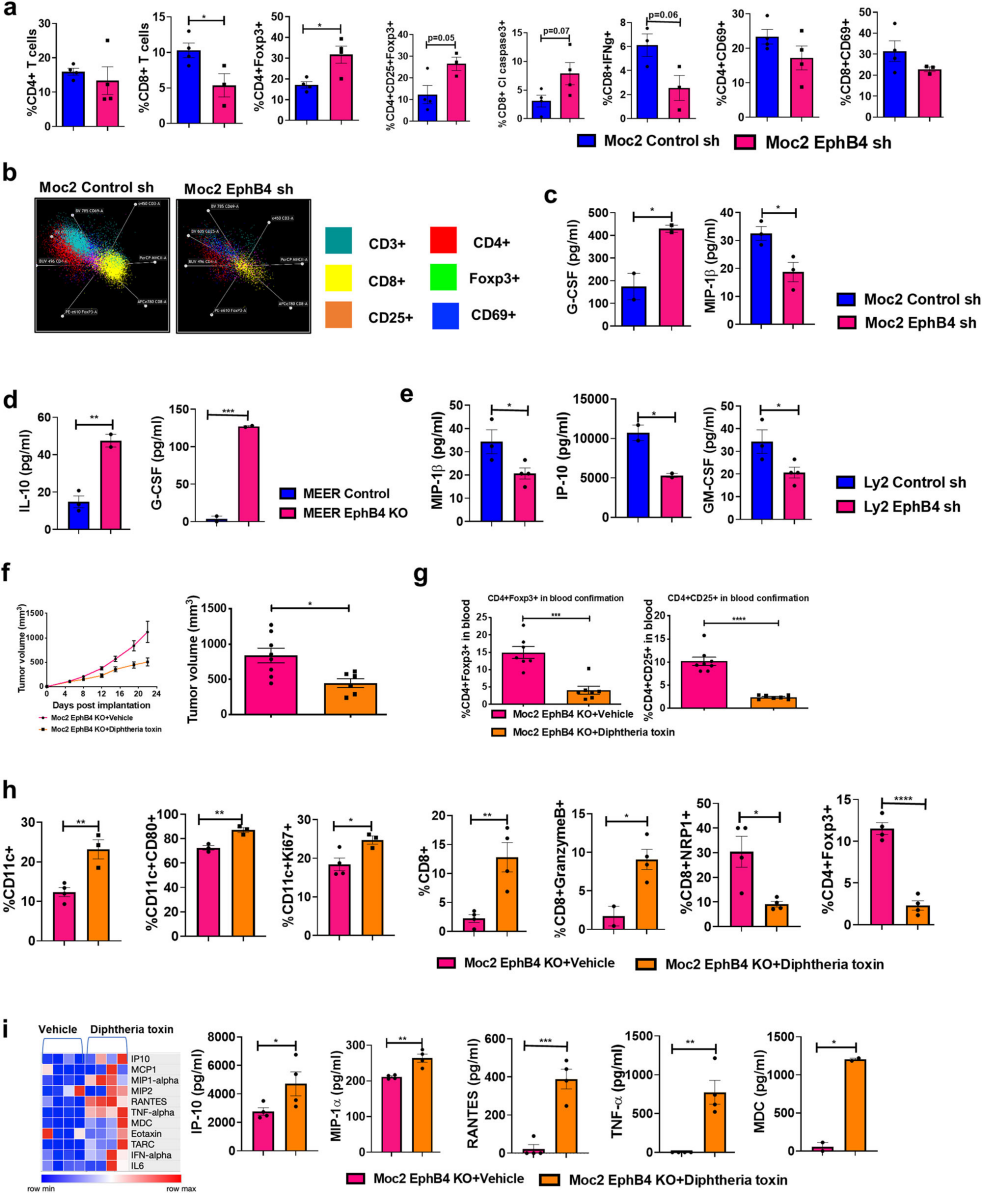
Genetic loss of Tregulatory cells (Tregs) reverses tumor growth enhancement associated with EphB4 downregulation on cancer cell. **a** Enhancement in Tregs, and increased apoptosis of CD8 T cells contribute to tumor immune remodeling because of EphB4 knockdown in Moc2 tumors in an orthotopic model. *n* = 4/group [Control sh and EphB4 sh] for CD4+, CD4+ Foxp3+, CD4 + CD69 + , CD8 + Cl caspase3+ cells; *n* = 4 [Control sh], *n* = 3 [EphB4 sh] for CD25 + Foxp3 + , CD8 + , CD8 + CD69+ cells; *n* = 3/group [Control sh and EphB4 sh] for CD8 + IFNg+ cells. **b** Representative radar plots illustrating differences in immune infiltrating populations in the control shRNA and EphB4 shRNA tumors. Loss of cancer cell-EphB4 altered the levels of inflammatory cytokines/chemokines in Moc2 *n* = 3/group [Control sh and EphB4 sh] for MIP-1β; *n* = 2/group [Control sh and EphB4 sh] for G-CSF (**c**), MEER *n* = 3 [Control sh], *n* = 2 [EphB4 sh] for IL-10; *n* = 2/group [Control sh and EphB4 sh] for G-CSF (**d**), and Ly2 *n* = 3 [Control sh], *n* = 4 [EphB4 sh] for MIP-1β and GM-CSF; *n* = 2/group [Control sh and EphB4 sh] for IP-10 (**e**). **f** The accelerated tumor growth because of EphB4 loss on cancer cells is reversed by depleting the Tregs in an inducible mouse model (*n* = 8). **g** Confirmation of Tregs is shown by flow cytometric analysis *n* = 7/group for CD4 + Foxp3 + ; *n* = 8 [Vehicle], *n* = 7 [Diphtheria toxin] for CD4 + CD25 + . Immune cell profiling in tumors *n* = 4/group for CD8 + ,CD8 + NRP1,CD4 + Foxp3 + ; *n* = 2 [Vehicle], *n* = 4 [Diphtheria toxin] for CD8 + GranzymeB + ; *n* = 4 [Vehicle], *n* = 3 [Diphtheria toxin] for CD11c + , CD11c + Ki67 + ; *n* = 3/group for CD11c + CD80 + is performed by flow cytometry (**h**). Changes in the cytokine/chemokines is observed in serum samples *n* = 4 for IP10, MIP1α, TNFα, RANTES; *n* = 2 for MDC obtained from EphB4 KO tumor implanted mice following Treg depletion as determined by mouse ProCartaPlex Immunoassay. The experiments were performed once with their own biological replicates. Data are shown as mean ± SEM. Comparison between the control and experimental groups was done by using two-sided Student’s *t*-test. *p*-values are indicated for the figures **a** CD8 *p = 0.04; CD4+ Foxp3+ *p = 0.016; **c** G-CSF *p = 0.03; MIP1-β *p = 0.031, **d** IL-10 **p = 0.0065; G-CSF ***p = 0.0009, **e** MIP-1β *p = 0.045; IP-10 *p = 0.033; GM-CSF *p = 0.045, **f** *p = 0.012 **g** ***p = 0.0002; ****p < 0.0001, **h** CD11c+ **p = 0.007; CD11c + CD80 + **p = 0.005; CD11c + Ki67+ *p = 0.032; CD8 + **p = 0.006; GranzymeB+ *p = 0.024; NRP1+ *p = 0.015; Foxp3 + ****p < 0.0001, **i** IP-10 *p = 0.03; MIP-1α *p = 0.004; RANTES ***p = 0.0006; TNFα **p = 0.002; MDC *p = 0.002.

To further correlate these changes in a cellular context, we performed immune profiling of EphB4 knockdown Moc2 tumors by flow cytometry. Our data showed a significant increase in the proportion of immunosuppressive T-regulatory cells (Tregs) without affecting the total T cell populations (Fig. 7a, b). The mean increase in Tregs was 1.84-fold greater in the EphB4 shRNA tumors compared to the controls (Fig. 7a) and consistent with the notion that Tregs are involved in immune evasion, inhibiting an effective anti-tumor immune response. Another observation evident after downregulation of EphB4 on cancer cells was the significant decrease in the influx of CD8 + T cells (Fig. 7a). We observed a trend towards reduction in their cytotoxicity and activation status marked by CD8 + IFNg+ T cells and CD8 + CD69 + T cells (Fig. 7a). Enhanced apoptosis (2.5-fold) was also found in the CD8 + T cell population as represented by CD8 + Cleaved caspase 3+ cells in the EphB4 knockdown tumors, restricting their survival in the tumor milieu (Fig. 7a).

Concordant with the increased immunosuppressive TME observed by flow cytometry were data from in vivo plasma samples from different tumor models examining changes in the circulating cytokine/chemokine profiles. A significant increase in G-CSF and IL-10 levels (Fig. 7c, d) was observed in the EphB4 knockdown group compared to the respective control mice. IL-10 is a pleiotropic cytokine that acts on antigen-presenting cells by blocking the expression of surface molecules implicated in T cell activation^35^, whereas G-CSF promotes Treg differentiation and immune tolerance^36^. In contrast, we noticed a significant decrease in the levels of pro-inflammatory chemokines/cytokines such as MIP-1β, IP-10/CXCL10, and GM-CSF (Fig. 7c, e) known to shape the TME by enhancing Teff cell chemotaxis, and activation^37,38^, suggesting that EphB4 loss on cancer cells induces an immunosuppressive phenotype in HNSCC.

### Genetic depletion of Tregs retards tumor growth acceleration mediated by EphB4 loss on cancer cells

Given the observed increase in Tregs with EphB4 knockdown on cancer cells and their established role in immunosuppression, we hypothesized that Tregs are mediating the accelerated tumor growth at least in part. We used a transgenic DEREG mouse model that have simian diphtheria toxin receptor-enhanced green fluorescent protein (DTR-eGFP) expression in functional CD4 + Foxp3+ Tregs, where administration of diphtheria toxin (DT) results in deletion of CD4 + Foxp3+ Tregs. Our data showed that implantation of Moc2 EphB4 KO tumors in DEREG mice resulted in a significant 2-fold reduction in tumor volume compared to the tumors implanted in control mice (Fig. 7f). Loss of Treg cells was confirmed by flow cytometry (Fig. 7g).

Flow cytometric analysis showed that implanting EphB4 KO cells in the DEREG mice significantly enhanced the infiltration of dendritic cells (DCs) and also increased DC proliferation and activation status as demonstrated by an increase in the CD11c + Ki67+ cells and CD11c + CD80+ cells in the tumors (Fig. 7h). We also noted a significant increase in the overall proportion of CD8+ T cells and CD8+ T cell cytotoxic activity as represented by CD8 + GranzymeB+ T cells in the DEREG mice implanted with Moc2 EphB4 KO tumors compared to the control group (Fig. 7h). This was accompanied by a significant decline in the CD8+ T cells expressing NRP1, a marker known to negatively regulate CD8+ T cell immune response^39^, in the Moc2 EphB4 KO tumors in the DEREG group (Fig. 7h). Cytokine/chemokine profiling by mesoscale U-plex assay further showed that, in the Moc2 EphB4 KO tumor-bearing DEREG mice, there was an increase in the secretory levels of IP-10/CXCL10, MIP-1α, RANTES, TNF-α, and MDC/CCL22 (Fig. 7i), all of which have been shown to play an integral role in DC recruitment to the tumor enhancing responsiveness towards DC-based immunotherapy agents, resulting in maximal control of tumor growth^40–44^. Together, these data suggest that Tregs partly contribute to the non-tumor cell-intrinsic mechanisms underlying the pro-tumor effects of EphB4 loss on tumor cells in vivo.

### The combined loss of ephrinB2 on tumor cells and vasculature reduces Tregs and enhances DC proliferation and Teff activation

**Fig. 8:**
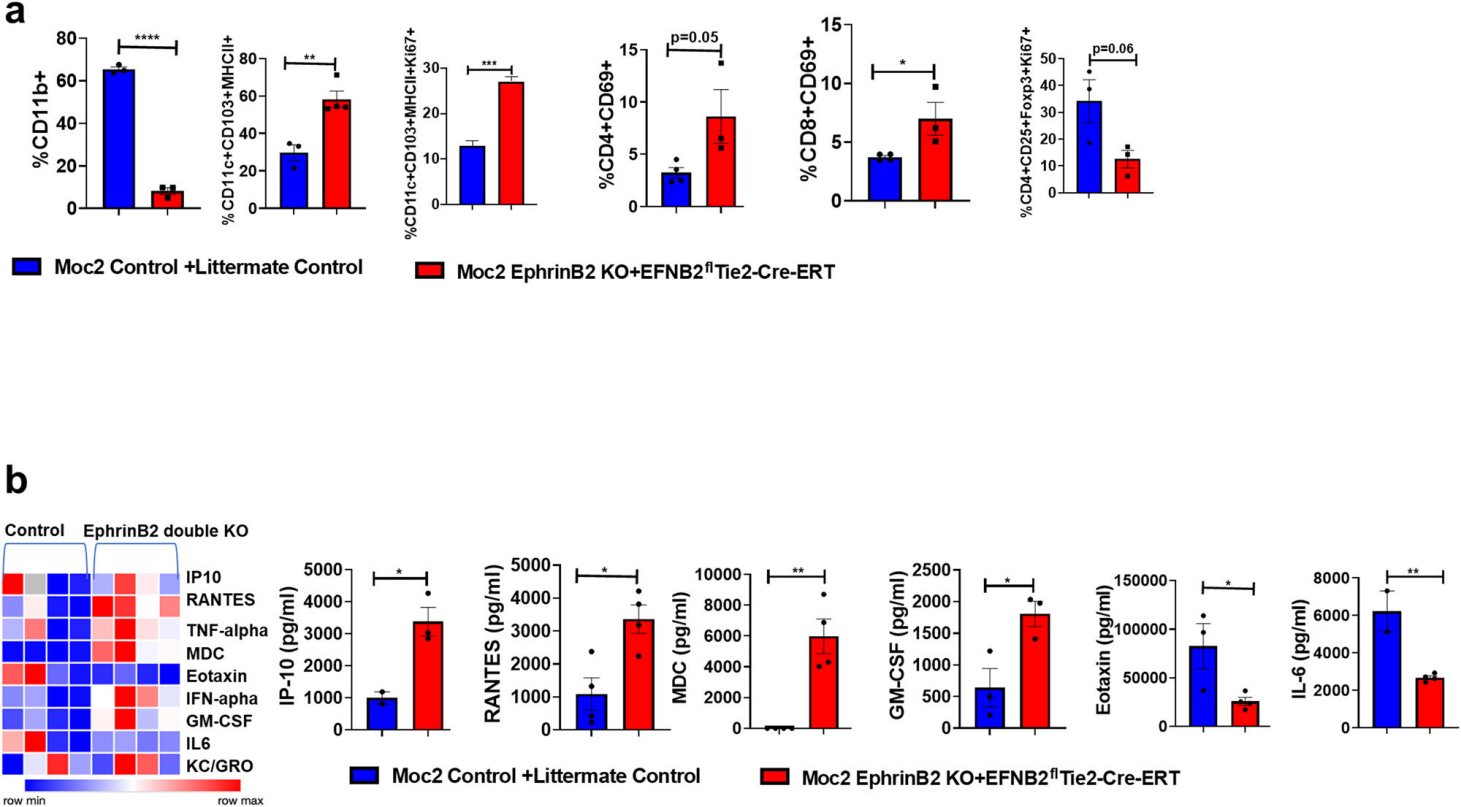
Absence of ephrinB2 on tumor cells and vasculature reconditions the immune TME in a genetically engineered Moc2 tumor model. **a** Tumors from the control and ephrinB2 double knockout (EphrinB2 KO + EFNB2^fl/fl^Tie2-Cre-ERT) groups were subjected to flow cytometry to determine changes in the immune profiles of T lymphocytes and dendritic cells. (*n* = 3/group) for CD11b+; *n* = 3 [control], *n* = 4 [ephrinB2 double knockout] for CD11c + CD103 + MHCII + and CD11c + CD103 + MHCII + Ki67 + ; *n* = 3/group for CD4 + CD25 + Foxp3 + Ki67+; *n* = 4 [control], *n* = 3 [ephrinB2 double knockout] for CD8 + CD69 + ; CD4 + CD69+ cells. **b** Serum samples from these mice *n* = 4 for MDC and RANTES; *n* = 2 [control], *n* = 3 [ephrinB2 double knockout] for IP-10; *n* = 3 [control], *n* = 4 [ephrinB2 double knockout] for Eotaxin; *n* = 2 [control], *n* = 4 [ephrinB2 double knockout] for IL6; *n* = 3 for GM-CSF was analyzed in a mesoscale cytokine assay to determine levels of circulating chemokines/cytokines affected by the loss of ephrinB2 in cancer cell and vascular compartments. For flow cytometric immune profiling, CD45 cells were used as a parent gate. Data are shown as mean ± SEM. Control mice refers to the littermate controls implanted with Moc2 control KO tumors. The experiments were performed once with their own biological replicates. Comparison between the control and experimental groups was done by using two-sided Student’s *t*-test. *p*-values are indicated for the figures: **a** ****p < 0.0001; **p = 0.006; ***p = 0.0002; *p = 0.039, **b** IP10 *p = 0.02; RANTES *p = 0.013; MDC **p = 0.0017; GM-CSF *p = 0.031; Eotaxin *p = 0.036; IL-6 **p = 0.006.

It is known that aberrant tumor vasculature fosters the development of an immunosuppressive TME^21^. Therefore, we investigated whether the double knockout of ephrinB2 on the vasculature and the cancer cells shown to induce vascular normalization (Fig. 3) inhibits tumor growth by relieving immunosuppression in HNSCC. Compared to the control group, flow cytometric analysis of tumors harvested from the ephrinB2 double knockout group showed significant changes in both myeloid cell and T cell populations. In particular, the CD11c + MHCII + CD103 + DCs increased by approximately 18% as did the proliferation (measured by Ki67) of these cells intratumorally (Fig. 8a). We also observed a significant enhancement in the percentage of activated CD8 + CD69 + T cells in the ephrinB2 double knockout cohort (Fig. 8a). This was in contrast to the immunosuppressive Tregs, which showed a trend towards decreased influx and proliferation in the ephrinB2 double knockout mice compared to the controls (Fig. 8a).

A multi-cytokine/chemokine mesoscale assay further supported these findings, with a significant increase in the circulating levels of chemokines such as IP-10/CXCL10, a key factor responsible for enhancing recruitment of T effector cells in the tumor, in the ephrinB2 double knockout mice compared to the control mice (Fig. 8b). We also noticed a significant enhancement in the secretion of RANTES, GM-CSF, and MDC in tumor-bearing mice with loss of ephrinB2 on cancer cells and vasculature. These chemokines/cytokines are important for the recruitment of antigen-presenting cells such as DCs that in turn facilitate the effector functions of T cell populations^38,45,46^, and contribute towards maximal inhibition of tumor progression (Fig. 8b). In contrast, levels of eotaxin, and IL-6 were significantly lower in the ephrinB2 double knockout mice (Fig. 8b). These factors have been shown to induce immunosuppression by either promoting the proportion of immunosuppressive Tregs or by activating alternate immunosuppressive pathways such as STAT5^47–49^. Collectively, these data support a pro-immunogenic effect associated with the dual ephrinB2 knockout as manifested by the increase in the activation and proliferation of dendritic cells, Teff cell activation, reduction in Tregs, and a chemokine profile that supports these functions.

### High EPHB4-low EFNB2 correlates with better overall survival and progression-free interval in HNSCC patients

**Fig. 9:**
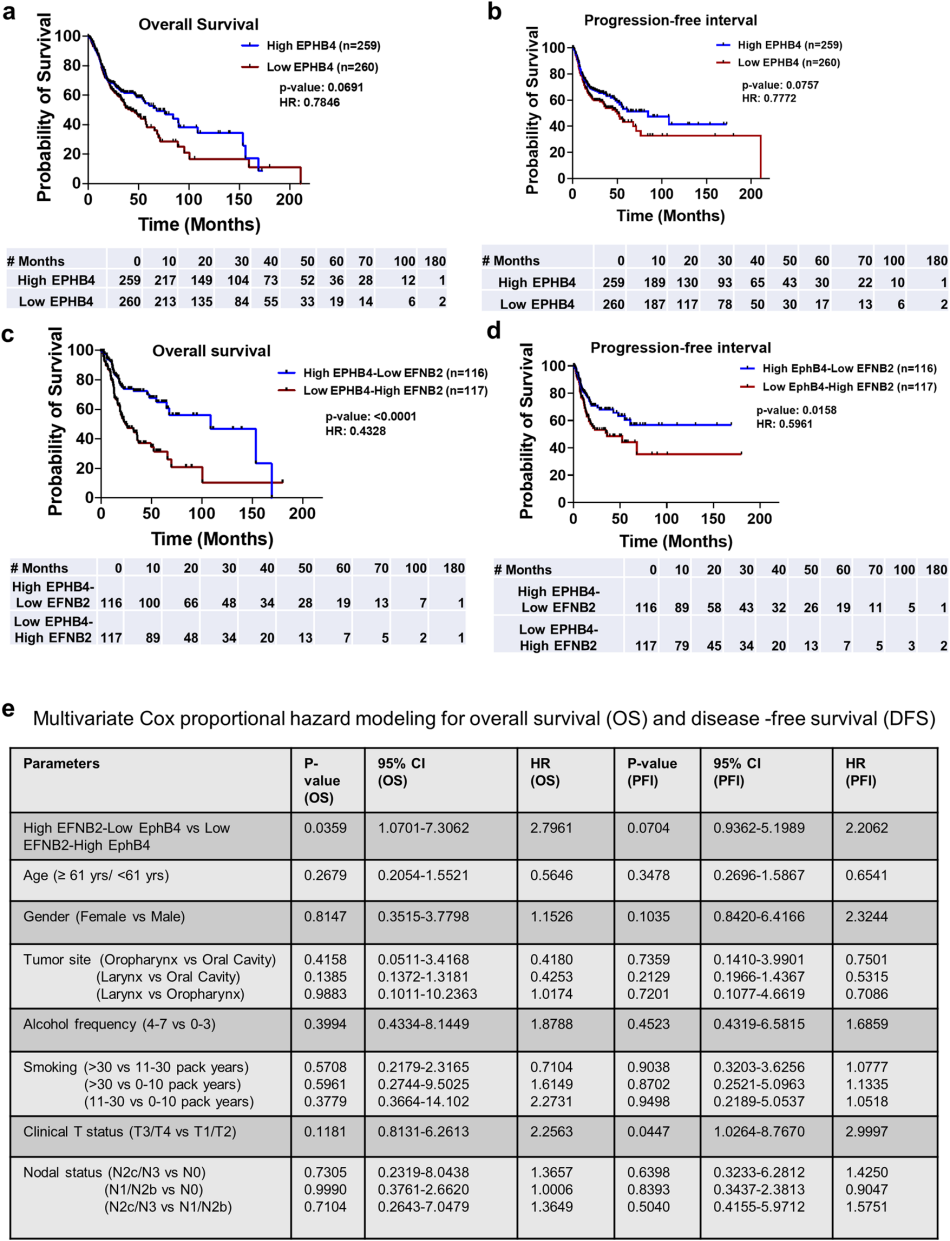
High EPHB4-low EFNB2 correlates with better overall and disease-free survival in HNSCC patients. TCGA database was interrogated and **a** overall survival (OS) and **b** progression-free interval (PFI) was calculated in HNSCC patients expressing high and low levels of EPHB4 by Kaplan–Meir method using log-rank test for comparison. The number of patients analyzed is mentioned in the respective figures. Data represented in **c** and **d** corresponds to better overall survival and progression-free interval (PFI) in patients with high EPHB4-low EFNB2 compared to low EPHB4-high EFNB2 cohort. **e** Multivariate cox analysis for OS and DFS was performed on TCGA HNSCC patients with high EPHB4-low EFNB2 controlling for age, gender, disease site, alcohol consumption, smoking, and tumor size (T), and lymph node status. HR, hazard ratio, CI, confidence interval.

We interrogated the TCGA database to determine the mRNA expression of EPHB4 across HNSCC patients. Patients were stratified into high and low expressors of EPHB4 based on the median expression of EPHB4. We observed that the cohort with high EphB4 expression showed a trend towards significance and correlated with improved survival compared to the low EphB4 group (Hazard ratio: 0.7846; CI: 0.6024–1.022; *p*-value: 0.0691) (Fig. 9a). Similarly, the progression-free interval for the high EPHB4 group was also greater compared to the low EPHB4 group (Hazard ratio: 0.7772; CI: 0.5877–1.028; *p*-value: 0.0757) (Fig. 9b).

We performed further analysis in the TCGA database to investigate the correlation of both EphB4 and EFNB2 with survival in concordance with our hypothesis. The HNSCC patient population with high EPHB4-low EFNB2 showed a significant increase in the overall survival compared to the low EPHB4-high EFNB2 cohort (Hazard ratio: 0.4328; CI: 0.2921–0.6411; *p*-value: <0.0001) (Fig. 9c). The progression-free interval showed a similar pattern in the high EPHB4-low EFNB2 vs. low EPHB4-high EFNB2 cohort (Hazard ratio: 0.5961; CI: 0.3910–0.9089; *p*-value: 0.0158) (Fig. 9d) suggesting that high EPHB4 and low EFNB2 can be used as a correlate for survival outcomes in HNSCC patients. The significant correlation of high EphB4-low EFNB2 TCGA HNSCC cohort with improved with significant overall survival persisted on multivariate analysis (Fig. 9e).

Further interrogation of HNSCC TCGA dataset revealed differential set of genes that are upregulated or downregulated in the low EPHB4-high EFNB2 vs high EPHB4-low EFNB2 cohort (Supplementary Fig. 21a). Since RNA-seq data in our preclinical model displayed transcriptomic changes following EphB4 loss on the cancer cell particularly in pathways regulating angiogenesis, cell survival, and IFN-gamma related gene signatures, we expanded our analysis to the HNSCC TCGA datasets of interest. We observed significant induction of GO_ Epithelial cell proliferation, WikiPathway_VEGFAVEGFR2 signaling, and Hallmark Interferon gamma response pathway as shown in Supplementary Fig. 21b–d. However, TCGA analysis did not show any significant correlation between EPHA4 vs EPHB4 in HNSCC patient (Supplementary Fig. 22). The discordance between the TCGA data and our in vivo findings can be attributed to the fact that in our study, EphA4 was upregulated only when EphB4 was knocked down on the tumor cells.

### HNSCC patients with clinical response to cetuximab therapy demonstrated low levels of ephrinB2 and high levels of EphB4 in the tumor specimens

The clinical success of targeted tyrosine kinase inhibitors is hampered by poor outcomes and development of therapeutic resistance. We sought to understand whether EphB4 and ephrinB2 levels are involved in determining the clinical response toward anti-EGFR monoclonal antibody, cetuximab. Patient tumor specimens (non-responders=13; responders=9) obtained from a clinical trial NCT01218048 were subjected to multispectral VECTRA staining for epithelial cell marker cytokeratin 7 (CK7), EphB4, and ephrinB2. Supplementary Fig. 23a shows representative sections from a responder (patient 27) and a non-responder (patient 6) stained with CK7, EphB4, and ephrinB2 markers and Supplementary Fig. 23b, c represent heat-maps and tables generated following mean quantitative analysis of the total ephrinB2+ cell counts, %CK7 + ephrinB2, EphB4+ cell counts, and %CK7 + EphB4 in responders and non-responders pre- and post-cetuximab treatment. The data demonstrated that a greater percentage of responders (77.77%) showed a decrease in the levels of ephrinB2 on CK7+ cells post-treatment compared to the non-responders (46.15%) (Supplementary Fig. 23b). Similarly, a greater number of responders (55.55%) have high total EphB4 cell counts post-cetuximab therapy compared to the non-responders (38.46%) (Supplementary Fig. 23c). These data, although correlational, indicate that high EphB4 and low ephrinB2 may be helpful in determining with the clinical response post-cetuximab therapy in HNSCC patients.

## Discussion

Multiple reports have unraveled the aberrant nature of Eph-ephrin signaling in human malignancies^11,50,51^. This has led to the development of therapeutic strategies that are currently being evaluated in a spectrum of pre-clinical and clinical studies. This includes agents that interfere with the Eph-ephrin signaling, such as small-molecule kinase inhibitors, peptides, short hairpin RNAs, recombinant fusion proteins, and monoclonal antibodies^52^. They work either by antagonizing the Eph-ephrin interaction or by targeting the Eph ectodomain or ephrin ectodomain, thus modulating the Eph or ephrin activity^52^. However, the promiscuous and dichotomous nature of the Eph-ephrin interactions, lack of unique intracellular targets, and disparate biological outcomes have posed unique challenges in the path of targeted drug development. It also raises the question of whether inhibition of either the receptor or the ligand is negating the beneficial effects of its counterpart^15^. Therefore, to discern the underlying mechanism of action and to reliably predict the biological response following Eph-ephrin intervention, it is necessary to understand the intricate complexity associated with receptor and ligand signaling on different cellular compartments including the tumor and its microenvironment. This becomes even more critical since the majority of clinical trials in the oncology space including HNSCC fail due to lack of basic understanding of complex pro-tumor mechanisms^53,54^. This study investigates the effects of targeting EphB4-ephrinB2 interactions separately in the HNSCC tumor and its microenvironment. Within the TME, we find that EphB4 acts as a tumor suppressor in both paracrine and autocrine fashion. However, stimulation of EphB4 alone, in the absence of pro-tumor ligand signaling, fails to impart any anti-tumoral benefit. The reverse is not true, as targeting ephrinB2 will result in an anti-oncogenic benefit as long as EphB4 receptor signaling is not inhibited. There are reports in the literature suggesting that activation of ephrinB2-EphB4 pair can lead to metastatic spread. However, we did not observe any evident changes in regional or distant metastasis in the models utilized in the current study. The lack of such gross metastatic effects can be attributed to the fact that local disease progression dictated the morbidity and mortality rates in our rodent models. This presents one of the limitations of the current study. The question is currently being addressed in the models where metastasis drives the pattern of treatment failure in a separate investigation. In addition to the vascular endothelium, ephrinB2 is also expressed on the lymphatic cells and its selective deletion in lymphatic endothelium using inducible Prox1-CreER^T2^ mouse model compromised the integrity of lymphatic endothelial junctions and increased leakiness^55^. This is consistent with previous reports where inhibition of ephrinB2 signaling using a single chain Fv antibody fragment has been reported to reduce the number of lymphatic vessels and permeability^56^. Genetic deletion of PDZ binding domain of ephrinB2 was also shown to remodel lymphatic vasculature during development^57^, a process that affects likely forward and reverse signaling^58^. The question pertaining to the presence of ephrinB2 on lymphatics and how its knockdown affects HSNCC tumor growth and microenvironment is a subject of an ongoing investigation.

Our findings underscore the importance of designing therapeutics that manipulate and block ephrinB2 signaling while avoiding any interference with EphB4 receptor signaling. Our study demonstrates how the compensatory mechanisms by upregulated EphA4 come into the concert when the preferred binding partner of ephrinB2, EphB4, is lost and by inhibiting EphA4 signaling, tumor growth inhibition can be effectively restored in HNSCC. In this scenario, exploiting available candidates that target compensatory signals, such as EphA4, when EphB4 signaling is impaired might provide therapeutic benefit, particularly if administered sequentially with a drug that inhibits bi-directional signaling. A separate study in the laboratory is currently underway to determine the role of EphA4 in the absence of EphB4 on the HNSCC tumors. As EphB4 signaling on the cancer cells acts as a tumor suppressor and represents a major target, combining available drug candidates that block bi-directional signaling with potent cytotoxic therapies, such as radiation therapy, would be potentially beneficial. This would likely render EphB4 signaling on the cancer cell irrelevant while the drug is left to target ephrinB2 tumor-promoting effect on the tumor cells and within the vascular compartment of the TME. Given the dual challenge of delivering targeted antibodies to the vascular endothelial cells while targeting the cancer cell itself with conventional radiation therapy, such a combination stands to achieve direct targeting of the cancer cell and to overcome the difficulties of endothelial cell targeting.

EphB4 is shown to be upregulated in a variety of human cancers, including head and neck cancers^59^. A question that needs to be addressed is, why would it be evolutionarily beneficial to upregulate a tumor suppressor on the cancer cell during malignant transformation? EphB4 is a high-affinity binding receptor for ephrinB2; and if ephrinB2 is a master tumor promoter within the TME, then it is sensible to upregulate EphB4 so its extracellular domain can be used for reverse signaling by ephrinB2. Here we show that, in tumor models where there is sufficient expression of ephrinB2 on the cancer cell, targeting ephrinB2 on vessel alone or cancer cell alone results in a modest tumor growth delay. This is likely due to the contribution of tumor-promoting effects by ephrinB2 signaling on the cancer cell, as it is only when both cancer cell and vascular ephrinB2 are knocked out that significant anti-angiogenic effect and meaningful growth delay are observed in vivo. In contrast, knockdown of EphB4, particularly its intracellular domain, increases cancer cell viability, enhances VEGF secretion, and angiogenesis. Designing novel targeted therapeutics against ephrinB2 that can selectively access both tumor cells and the vasculature and performs its function without inhibiting its receptor EphB4 signaling will be critical to establish a meaningful effect on tumor growth. Currently, no such therapeutic agent exists and therefore efforts need to be directed to design such rational therapeutic agents to allow maximal clinical benefit.

Another finding of our study is the invigoration of immune response with the double knockout of ephrinB2 on the vasculature and the cancer cell. We had previously established that systemic Treg depletion alone does not cause tumor growth inhibition^60^ and that modulation of dendritic cell-Treg cross-talk into a less tolerogenic and more inflammatory status is required in these cold tumors. In our study, we demonstrate that double ephrinB2 knockout achieves this goal efficiently. Cancer cell knockdown of EphB4, on the other hand, yields an influx of immunosuppressive Tregs. While it is unclear whether the increase in Tregs is related to enhanced vascularity or trans-endothelial migration, we show that genetic depletion of Tregs reverses the accelerated tumor growth observed with EphB4 knockdown on the cancer cell. These data add to the growing body of literature showing anti-angiogenic agents acting as potentiators of immunotherapeutic targets and favoring tumor rejection in different models^61^. The exact mechanisms by which these are mediated remain to be determined and elucidating the contribution of the identified key targets that are hyperactivated or downregulated as a result of EphB4 or ephrinB2 knockdown should open the doors for the discovery of immunotherapies.

Taken together, our study highlights the complexity of context-dependent signaling emanated from the EphB4-ephrinB2 axis in different cellular compartments and its impact on vascular and immune cell modeling. These findings will help in differentiating between the pro- and the anti-tumor drivers on the cancer cells and within the TME and present opportunities for drug interventions aimed at blocking ephrinB2 signaling for maximal therapeutic benefit.

## Methods

### Cell culture and reagents

The HNSCC murine cell lines were obtained as follows: Moc2 cell line from Dr. Ravindra Uppaluri (Dana-Farber Cancer Institute, Boston, MA), MEER cell line from Dr. John Lee (Sanford Health, Sioux Falls, SD) and Ly2 cell line from Dr. Nadarajah Vigneswaran (University of Texas Health Science Center, Houston, TX). CUHN013 cells were obtained from Dr. Antonio Jimeno (Anschutz Medical Campus, Aurora, CO). The cells were cultured as described earlier^8,59,62^. bEND.3 cells were obtained from the lab of Dr. Jordan Jacobelli (Anschutz Medical Campus, Aurora, CO). STR analysis was conducted on the cell lines wherever applicable to ensure authentication. All cell lines in this study were within 12 passages and tested for mycoplasma contamination prior to their use in the experiments.

### Generation of stable cell lines

Lentivirus encapsulated shRNA vectors (pLKO.5, Sigma) were purchased from the University of Colorado Cancer Center Functional Genomics Facility (Anschutz Medical Campus, Aurora, CO). EphrinB2 knockdown was achieved by transducing HNSCC cells with shRNA against murine ephrinB2 (clone# TRCN0000336422) or EphB4 (clone# TRCN0000274508). Cells were transduced with control shRNA (clone # SHC216) in parallel. Transduced Moc2 cells were selected with a 1 μg/ml dose of puromycin for ephrinB2 plasmid and increased to a 10 μg/ml dose for EphB4 plasmid. Ly2 EphB4 and ephrinB2 knockdown clones were selected using a 2 μg/ml dose of puromycin. For CRISPR knockout clones, HNSCC cells were transfected with PX458 control plasmid or PX458 containing gRNA targeting ephrinB2 (Efnb2 gRNA GGTCTGGCACAGTTGAGCAG) or EphB4 using Fugene reagent according to the manufacturer’s instructions. Screening of positive clones was performed by western blotting. The control dominant-negative (EGFP-F), EphB4 dominant-negative (EphB4ΔC-EGFP), and ephrinB2 dominant-negative (ephrinB2ΔC-EGFP) constructs were obtained from the lab of Dr. Elena Pasquale (Sanford Burnham Prebys Medical Discovery Institute, San Diego, CA, USA). The human CUHN013 cells were transfected with dominant-negative constructs using Fugene reagent, clones were selected using GFP reporter on a flow sorter, followed by expansion for in vivo implantation. Data supporting phosphorylation of EphB4 and ephrinB2 in the CUHN013 dominant-negative tumors are shown in Supplementary Fig. 24.

### In vivo models

All mice were handled and euthanized consistent with the ethics guidelines and conditions set and overseen by the University of Colorado, Anschutz Medical Campus Animal Care and Use Committee. The study has been approved by the Institutional Animal Care and Use Committee (IACUC). For breeding mouse colony, light cycle, temperature and humidity were controlled in the mouse housing area and dark cycle interruptions were avoided. A 14 h light/10 h dark cycle or 12 light/12 dark cycle is routinely used. Temperatures of 65–75 °F (~18–23 °C) with 40–60% humidity are maintained. Breeder chow diet is provided to help nursing female mice. Based on our approved animal protocol (Protocol# 00250), if the implanted tumor measurement exceeded 2000 mm^3^ in a single plane, or if the tumors become ulcerated and mice have longer than a week remaining in the study, they were euthanized. In situations where mice had to be kept in the study if the tumor measurement exceeded the 2000 mm^3^ limit, mice were monitored daily, applied with topical antibiotic (if there was an ulcerated tumor) and cared for by the lab personnel and the veterinary staff at the Anschutz Medical Campus. There was one mouse in the EphB4 dominant-negative group (included in Fig. 4a) that exceeded the 2000 mm^3^ limit and was monitored for longer than a week. This was an aggressively growing tumor and validation of the growth increase (versus inflammation) was essential to address the scientific question. Therefore, we had to keep this mouse under daily supervision for longer than 7 day time-period. No adverse health report was documented for this mouse during the time of extended monitoring. Euthanasia was performed as per AVMA Guidelines for the Euthanasia of Animals: 2020 Edition using the compressed Co2 gas inhalation method in the induction chamber followed by cervical dislocation.

#### EphrinB2 and EphB4 conditional knockout mouse models

Breeding pairs of EFNB2^fl/fl^Tie2-Cre-ERT, EphB4^fl/fl^Tie2-Cre-ERT, and EphB4^fl/fl^Col1A2-Cre-ERT mice were obtained from Dr. Mohit Kapoor’s lab (University Health Network, University of Toronto, Canada) and maintained at the Anschutz Medical Campus, Aurora mouse facility. Briefly, to generate mice in which ephrinB2/EphB4 is conditionally deleted specifically in Tie2-expressing cells on vasculature, C57BL/6 N mice carrying a tamoxifen-inducible Cre-recombinase [*Tie2-Cre-ERT2*] were crossed with *Efnb2*^*loxP/loxP*^ mice or *Ephb4*^*loxP/loxP*^ mice. To delete ephrinB2/EphB4 from vasculature, five consecutive injections of 4-hydoxytamoxifen suspension (70% Z-isomer; Sigma; 0.1 ml volume, 10 mg/ml) were administered intraperitoneally to 4–5 weeks old mice, with corn oil administered littermates as controls. Specific deletion of *Efnb2* was confirmed using the following: Primer pair 1 (forward, 5′-TAGCC ATCCC TTGGA ATACG-3′, and reverse, 5′-TTGGC GCGCC CCTTT CGAAG-3′) was used to detect a 456-bp fragment derived from an allele(s) with undeleted Exon 1. Primer pair 2 (forward, 5′-CTAAG GCTCT CAGCC TCGTG-3′, and reverse, 5′-TTGGC GCGCC CCTTT CGAAG-3′) was used to detect a 291-bp fragment derived from an allele(s) with deleted Exon 1. Primer pair 3 (forward, 5′-CGAGT GATGA GGTTC GCAAG-3′, and reverse, 5′- TGAGT GAACG AACCT GGTCG-3′) was used to detect the 450-bp Cre recombinase gene. Primer pair 4 (forward, 5′-GCCCT TAAAG GACCG ACTTC-3′, and reverse, 5′-GCCTA ACGCT GGAGA AAGTG-3′) was used to amplify a 271-bp fragment from the floxed EphB4 allele and a 133-bp fragment from the WT allele. PCR genotyping results are shown in Supplementary Fig. 25. Experiments involving genetically engineered mouse models were performed using both male and female mice (*n* = 7–8) in the age group between 7–8 weeks. For Moc2 EphB4 or ephrinB2 modified or control tumors (100,000–125,000 cells/mouse) were implanted in the conditional knockout mouse models and tumor growth was monitored longitudinally using a digital caliper. Tumor volume was calculated using the formula: [(smaller diameter)^2^x (longer diameter)]/2.

#### Immunocompetent mouse model

For immunocompetent mouse model studies, 5- to 6-week-old female BALB/c mice (Charles River Laboratories) or C57BL/6 mice (Jackson Laboratories) were used. Control clones or stable clones of Moc2 (100,000–125,000 cells/mouse), MEER (20,000 cells/mouse), and Ly2 (1,000,000 cells/mouse) cells with either knockdown or complete knockout of EphB4 or ephrinB2 were implanted orthotopically in the buccal cavity as described^8^. Tumor growth was monitored using a digital caliper as described above. The experiment was replicated two times.

For the in vivo experiment in which either ephrinB2-Fc or control-Fc was administered, mice were randomized into two groups once the tumors reached ~100–150 mm^3^ in tumor volume. EphrinB2-Fc (R&D Systems, Minneapolis, MN, USA) is a recombinant protein comprising of an extracellular domain of ephrinB2 dimerized by tagging with the Fc protein of IgG_1_. A dose of 30 μg of ephrinB2-Fc or control-Fc was administered intraperitoneally to each mouse bi-weekly throughout the experiment^51^. The experiment was replicated three times. For nilotinib-treated tumors, 40 mg/kg dose (5x/week; 8 doses) of Nilotinib dissolved in the diluent: 4%DMSO + 30% PEG300, %Tween80 + dH_2_O was administered as oral gavage in the experimental mice. Control mice were treated with diluent alone. Dasatinib was administered by oral gavage at a dose of 20 mg/kg (5x/week) in 80 mM citric acid buffer (pH 3.0). Tumor tissue was harvested at the time of sacrifice and either fixed in 10% neutral buffered formalin or flash-frozen for further analysis.

#### Immunocompromised mouse model

For immunocompromised mouse model studies, 5–6 weeks old female athymic nude mice were used. The HNSCC PDX tumor-derived CUHN013 cell line was obtained from Dr. Antonio Jimeno’s lab (University of Colorado, Anschutz Medical Campus, Aurora, CO). The stable cell lines generated following the transfection of CUHN013 cells with either non-specific control plasmids, or EphB4 shRNA, ephrinB2 shRNA, or the dominant-negative constructs were implanted in the flank region and tumor growth was monitored as described^8^. The experiment was replicated two times.

#### DEREG mouse model

Mice with a diphtheria toxin receptor attached to the forkhead boxp3 gene, or DEREG mice, were obtained from the laboratory of Dr. Edward Chan (National Jewish Health, Denver, CO). Both male and female mice (5–6 weeks old) were used in this experiment. Moc2 EphB4 KO tumors were implanted orthotopically in the buccal cavity of these mice or their respective controls and growth was monitored over time using digital calipers. To systemically deplete Tregulatory cells, high dose (1 μg in 100 μl volume) of diphtheria toxin (DT) was injected on day 2, and day 1 prior to tumor implantation and on d5 post-tumor implantation. The dose of DT was reduced to 0.5 μg in 100 μl volume for maintenance and was injected twice a week throughout the course of expt. Confirmation was done on the blood samples collected 4 days after the DT injection at d19 post-implant by flow cytometry.

Data from in vivo studies are presented either in the form of spaghetti plots showing a temporal change in tumor volumes or dot plots or bar plots. The dot plot or bar plot includes data from the mice that were alive at that particular time-point of analysis as indicated in the Figure legend.

### Immunofluorescence staining

Immunofluorescence staining was performed on paraffin-embedded sections fixed in 4% buffered formalin. Tumor tissue sectioned at 4 µm was deparaffinized and hydrated, and antigen epitope retrieval was performed by incubating the slides in antigen retrieval buffer (Vector Laboratories) for 10–15 min. After washing with TBS, sections were incubated with primary antibodies overnight at 4 °C. Primary antibodies against CD31 (1:100) were obtained from Cell Signaling. Anti-NG2 antibody (1:100) was obtained from Invitrogen, and Anti-alpha SMA (1:200) and Anti-Col1A2 (1:100) were purchased from Abcam. Anti-PCNA (1:200) antibody was purchased from BD Transduction Laboratories. Anti-VE-Cadherin (1:100) was obtained from Biolegend. Antibodies against EphB4 (1 μg/ml) and ephrinB2 (1:100) were provided by Vasgene Therapeutics Inc. Anti-EphB4 antibody was also purchased from Invitrogen or Sigma (1:100) and anti-Tie2 antibody (1 μg/ml) was obtained from R&D Systems. The primary antibody incubation step was followed by washing and further incubation with fluorescent-tagged IgG secondary antibody (1:400 dilution, Life Technologies). Nuclei were counterstained with 6-diamidino-2-phenylindole dihydrochloride hydrate (DAPI). Images were captured using an x20 objective using a Nikon fluorescence or Olympus confocal microscope. For each experimental and control group, images from 6–8 random fields were captured, two sets were analyzed, and Image J software (NIH) was used for quantitative analysis. Each experiment was replicated in two sets, and statistical analysis was done using a two-sided Student’s *t*-test.

### Multispectral VECTRA staining

Multiplex imaging of Moc2 tumor tissue was performed at the Human Immune Monitoring Shared Resource at the University of Colorado School of Medicine using the Perkin Elmer Vectra Polaris instrument.

#### Mouse tumor tissue

Formalin-fixed, paraffin-embedded mouse tumor tissue were deparaffinized, heat-treated in antigen retrieval buffer, blocked, and incubated with primary antibody (EphB4, ephrinB2, CD31, and EpCAM), followed by horseradish peroxidase (HRP)-conjugated secondary antibody polymer, and HRP-reactive OPAL fluorescent reagents. The following OPAL fluorescent reagents were used on mouse tumor tissue: CD31: Opal 540-Yellow; EpCAM: Opal 570-Orange; EphB4: Opal 650 (magenta); EphrinB2: Opal 520 (green). To avoid further accumulation of fluorescent dyes in subsequent staining steps, slides were stripped in between each stain with heat treatment in antigen retrieval buffer. DAPI was used to stain nucleated cells.

#### HNSCC patient specimens

HNSCC patient specimens were obtained from the completed NCT01218048 (UPCI 08-013) phase II trial entitled “Erbitux Followed by Adjuvant Treatment with Chemoradiation and Erbitux for Locally Advanced Head and Neck Squamous Cell Carcinoma” from Dr. Robert Ferris’s lab (University of Pittsburgh, PA). All patients were seen in the Department of Otolaryngology at the University of Pittsburgh Medical Center, and specimens from patients were obtained by the written informed consent under the University of Pittsburgh IRB approved protocol. The study was conducted in accordance with the ethical standards of the Declaration of Helsinki. The study of preoperative, single-agent cetuximab treated patients had HNSCC tumor specimens collected before and after 4 weeks of cetuximab. Stage III/IV HNSCC patients (*n* = 22) were subsequently treated with definitive surgical resection and monitored for disease recurrence. Cetuximab was administered for a 3–4 week preoperative period and the clinical response was recorded. Clinical response was determined by comparing paired CT scans pre/post cetuximab, and quantifying tumor measurement by a dedicated head and neck radiologist blinded to patient status. Anatomic tumor measurements were recorded in two dimensions and the cohort was categorized into clinical “responders,” who demonstrated a reduction of 10–30% in tumor volume, or “non-responders,” whose tumors grew during this therapy^63^. We obtained sections of archival formalin-fixed, paraffin-embedded (FFPE) tissue on glass slide for immunofluorescence staining. Sections of primary HNSCC were stained using Opal multiplex according to the manufacturer’s protocol (PerkinElmer): EphB4: Opal 780 (magenta); EphrinB2: Opal 480 (green); Cytokeratin (CK): Opal 570 (yellow).

Slides were scanned and multispectral images of each region of interest were collected. Color images were analyzed with inForm software to unmix adjacent fluorochromes, subtract autofluorescence, segment the tissue, compare the location of cells, segment cellular membrane, cytoplasm, and nuclear regions, and phenotype cells according to morphology and cell marker expression.

### Western blotting and antibodies

Tumors were harvested and homogenized as described earlier^59^. Protein lysates (20–30 μg) were loaded onto 10–12% SDS-PAGE gels. Electrophoresis, blocking, antibody incubation, and detection were performed as described^59^. Blots were probed overnight at 4 °C with the respective antibodies. Anti-survivin (1:000), anti-Bcl-XL (1:1000), anti-p-c-Jun (1:1000), anti-Jun, anti-p-STAT3 (1:1000), anti-STAT3 (1:1000), anti-JAK2 (1:1000), anti-SHP2 (1:1000), anti-VEGF (1:500), anti-NRP1 (1:1000), anti-p-SHP1 (1:750), anti-SHP1 (1:750), and anti-β-actin (1:1000) HRP-conjugated antibodies were obtained from Cell Signaling Technology (Danvers, MA, USA). Anti-EphB4 (1:1000 or 0.5 μg/ml) antibody was purchased from Invitrogen (Carlsbad, CA, USA) or MilliporeSigma (Burlington, MA, USA), and anti-ephrin-B2 (1:750) was obtained from Abcam (Cambridge, MA, USA). Anti-EphA4 (1:750) antibody was obtained from Proteintech Group, Inc. (Rosemont, IL, USA). Anti-p-ephrinB2 (1:500) antibody was obtained from ThermoFisher Scientific. Goat anti-mouse, goat anti-rabbit, and donkey anti-goat horseradish peroxidase (HRP)-conjugated secondary antibodies were obtained from Sigma (St. Louis, MO, USA) and used at a dilution of 1:3000.

### Immunoprecipitation assay

Frozen tumors were homogenized in liquid nitrogen using a mortar and pestle followed by resuspension in 1x Cell Lysis Buffer containing protease and phosphatase inhibitors. Lysate was diluted to 1 μg/μL in lysis buffer and 250 μL of resulting lysate was incubated with 5 μg of anti-EphA4 antibody (Invitrogen) overnight at 4 °C with end-over-end rotation. Magnetic protein G beads (Bio-Rad) were washed three times with 1x PBS before addition to the lysate-antibody complexes. Beads were incubated with lysate-antibody complexes overnight at 4 °C with end-over-end rotation. Beads were washed three times with cold 1:1 lysis buffer:PBS, removing supernatant after each wash. Protein was eluted from beads by adding 1x sample loading buffer containing 1x sample reducing agent (Invitrogen) and boiling beads at 95 °C for 15 min. A total of 250 μg equivalent starting material was resolved on a 10% SDS-PAGE gel and transferred to Immuno-Blot PVDF membrane (Bio-Rad). Membranes were probed for total phosphotyrosine (Cell Signaling Technology).

### IncuCyte assay

HNSCC cells growing in a monolayer were harvested and plated in a 96-well plate at a density of 2000 cells/well either alone or in co-culture with bEND.3 cells. The cells were incubated at 37 °C in an incubator equipped with IncuCyte system to allow real-time monitoring of cell growth over a period of 4 days. The plate was scanned at different time intervals by using either 4x or 10x objective. Analysis was performed using the IncuCyte Zoom software.

### Flow cytometry

Tumors were harvested at day d23-d24 post-implantation and processed as described earlier^64^. Briefly, tumors were finely minced and placed in Hanks’ Balanced Salt Solution (HBSS) containing 200U of Collagenase III (Worthington, Lakewood, New Jersey, USA) for 30 min at 37 °C with gentle shaking every 10 min. After the incubation period, tumor pieces were passed through a 70 μm nylon mesh. The resulting cell suspension was centrifuged and resuspended in red blood cell lysis buffer for 3 min (Invitrogen, Carlsbad, California, USA). HBSS was added to inactivate RBC lysis buffer, cell suspensions were centrifuged, resuspended, and counted using an automated cell counter. For intracellular flow cytometric analysis, 2 × 10^6^ cells were plated in 6-well plates and cultured for 4 h in the presence of monensin to prevent the release of cytokines and PMA and ionomycin to stimulate cytokine production. After the incubation period, cells were incubated with a live/dead aqua viability stain kit (Invitrogen, Carlsbad, California, USA) for 30 min at 4 °C. Cells were centrifuged and then incubated with blocking agent FcgRIII/II (aCD16/CD32; eBioscience, San Diego, CA, USA). After blocking, cell labeling was performed by incubating cells with fluorescently conjugated antibodies listed in **Source file** “flow cytometry panel” tab. Flow cytometry was performed on a Yeti instrument (ZE5 Cell Analyzer; Bio-Rad). Data were analyzed using Kaluza 2.1 or Flow Jo v10.8 software. Gating strategies for flow analysis are represented in Supplementary Fig. 26.

### Multiplex immunoassay

The ProcartaPlex multiplex immunoassay kit was purchased from ThermoFisher Scientific and U-plex array was purchased from Meso Scale Diagnostics (MSD). Blood samples were collected at the end of the experiment from the control and experimental groups. Plasma or serum samples were collected at the time of sacrifice and separated from whole blood following the manufacturer’s instructions. Briefly, for the ProcartaPlex assay, antigen standards were prepared and incubated with magnetic beads. Plasma samples were incubated with the beads for 1–2 h. After washes with the wash buffer, samples were incubated with detection antibody mixture for 30 min at room temperature with shaking. Streptavidin labeled with PE fluorophore was later added to the samples followed by incubation for 30 min. The plate was analyzed further on a Luminex instrument. Samples were subjected to a U-plex array as described^8^.

### Ex Ovo Chick Chorioallantoic membrane (CAM) assay

CAM assay was performed on research-grade pre-incubated chicken eggs (1–17 days of age) obtained from Charles River Laboratories (Norwich, CT, USA). The protocol for assays involving chicken eggs was assessed and approved by the OLAR veterinary team at University of Colorado Denver, Anschutz Medical Campus. Upon arrival, eggs were incubated in an egg incubator with automatic rotation every 2 h for 3 days at 38 °C and 50–60% humidity. At day 3–4, the eggs were cracked open and their contents transferred to a sterile weigh boat. The yolk sac and the embryo were identified and assessed for viability for a beating heart. The weigh boats with the contents were then covered in a petri dish and incubated at 37 °C and 80–90% humidity for another 3–4 days. The cancer cells or the conditioned media were added to the ex ovo cultures and monitored over time to investigate the effect on the development of vasculature and embryo. Images were captured at different time-points and analyzed using angiogenesis analyzer tool (ImageJ software) in two sets.

### HUVEC tubule formation assay

Human umbilical vein endothelial cells (HUVECs) were plated at a density of 20,000 cells/well in a 96-well plate coated with growth-factor reduced matrigel. Before plating, cells were mixed in 50 μl conditioned media derived from HNSCC cells. At 6 h post-plating, cells were analyzed for the formation of tubular structures and images were captured using light microscopy using x10 objective. Tubes were counted, and the analysis was performed using ImageJ software.

### VEGF ELISA

Human and mouse VEGF ELISA kit was purchased from RayBiotech (Norcross, GA, USA) and R&D Systems (Minneapolis, MN, USA) respectively. VEGF concentration was detected in the conditioned media or serum samples by following a protocol according to the manufacturer’s instructions. The VEGF concentration was quantitated with comparison of the ELISA data using a standard curve obtained with known concentrations of cytokine.

### RNA-Sequencing analysis

Moc2 tumors were harvested and immediately frozen in liquid nitrogen. Tumor tissue was processed, and RNA was harvested using the RNA miniprep kit (Zymo Research, Irvine, CA, USA) as described earlier^60^. Sequencing and library prep was performed by The Genomics and Microarray Shared Resource at the University of Colorado Denver Cancer Center. PolyA selection was used for library prep and sequencing was performed on an Illumina NovaSEQ 6000 with 2 × 150 paired-end reads at a depth of 20 million reads per sample. The bioinformatics strategy outlined in the previous article was used^60^. Representative heatmaps are shown for the indicated samples. The volcano plot was created with R from the source (https://github.com/kevinblighe/EnhancedVolcano).

### Dynamic contrast-enhanced magnetic resonance imaging (DCE-MRI) analysis

Non-invasive measurements of tumor vascularity were performed on Moc2 control and Moc2 ephrinB2 double knockout mice to quantify the effects on vascular function. Briefly, the mice were anesthetized with an intraperitoneal injection of ketamine/xylazine (60/10 mg/kg), and a tail vein catheter filled with contrast agent was placed. The animal was positioned inside a warmed animal holder and inserted into the Bruker 9.4 T Tesla MRI scanner. All images were acquired using Bruker ParaVision 360.1.0 software as described^65^.

### Single-cell RNA Sequencing data interrogation from public databases

Publicly available single-cell RNA seq data at the University of California Santa Cruz (UCSC) Cancer Browser were interrogated to analyze ephrinB2 and EphB4 gene signature in 18 cases of oral cavity HNSCC patients at the time of surgical resection, either from the primary tumor or lymph node (LN) dissection^20^. Tissue was processed, and single-cell suspensions were sorted into different cell types followed by cDNA synthesis and library preparation^20^. Single-cell RNA seq and differential analysis were performed as described earlier^20^. For the Col1A single-cell analysis, Tumor immune single-cell hub (TISCH) database was used for the human HNSCC dataset^20^ (accession number: GSE103322) and murine dataset^66^ (accession number: GSE119352). The human HNSCC single-cell RNA seq publicly available data used in this study are available in the Gene Expression Omnibus database under accession code GSE103322. The murine single-cell RNA-seq data^66^ used in this study are available in the Gene Expression Omnibus database under accession code GSE119352.

### TCGA data analysis

The HNSCC TCGA data^67^ used in this study are available in the GDC legacy archive database [https://portal.gdc.cancer.gov/legacy-archive/search/f]. Survival analysis and disease-free survival for HNSCC were based on average mRNA expression. Overall survival (OS) and progression-free interval (PFI) were calculated by Kaplan–Meier method using log-rank tests for comparison. PFI was measured from the date of randomization of primary treatment to the time of disease recurrence. Death without documented recurrence was censored at the date of death. Univariate Cox proportional model and multivariate Cox proportional model was used to calculate the Hazard ratio (HR). Multivariate analysis was performed to control for age, gender, tumor site, alcohol consumption, smoking, tumor size (T), and lymph node (N) status. JMP 15 software was used for the multivariate analysis. Two-sided *P*-values were reported for all survival analyses. The HPV status was not included in our multivariate analysis due to the lack of the number of cases (fewer than 10 in each analyzed group). However, the distribution of both the HPV-positive and the HPV-negative cases within the high EFNB2-low EPHB4 and low EFNB2-high EPHB4 cohorts was not significantly different (Supplementary Fig. 27).

### Statistics and data reproducibility

The experiments were performed in the study using biological or technical replicates wherever applicable. Independent attempts were made to ensure data reproducibility. Both raw tumor volumes and fold changes are reported for the tumor growth experiments in vivo. Statistical analysis was performed using GraphPad Prism 9 software. Statistical analyses of differences were performed using two-sided Student's *t*-test or one-way ANOVA. The Dunnett post hoc test or Tukey’s multiple comparison test was used after ANOVA where multiple experimental groups were involved. A *P*-value of ≤0.05 was considered significant.

### Reporting summary

Further information on research design is available in the Nature Research Reporting Summary linked to this article.

## Supplementary information

Supplementary Information

Description of Additional Supplementary Files

Supplementary Data 1

Supplementary Data 2

Supplementary Data 3

Reporting Summary

## Data Availability

The human HNSCC single-cell RNA-seq publicly available data^20^ used in this study are available in the Gene Expression Omnibus database under the accession code GSE103322. The murine single-cell RNA-seq publicly available data^66^ used in this study are available in the Gene Expression Omnibus database under the accession code GSE119352. The HNSCC TCGA data^67^ used in this study are available in the GDC legacy archive database [https://portal.gdc.cancer.gov/legacy-archive/search/f]. The Moc2 RNA-seq tumor data generated in this study have been deposited in the Gene Expression Omnibus database under the accession code GSE201148. The remaining data are available within the Article, Supplementary Information, or Source Data file. Source data are provided in this paper.

## Source data

Source Data
