# Supplementary material for: EphB4 and ephrinB2 act in opposition in the head and neck tumor microenvironment
Source: Nat Commun. 2022 Jun 20;13:3535. doi: 10.1038/s41467-022-31124-7 (PMC9209511; doi:10.1038/s41467-022-31124-7)
Supplement: Supplementary file 1 — Supplementary Information [file 41467_2022_31124_MOESM1_ESM.pdf]

EphB4 and ephrinB2 act in opposition in the head and neck tumor microenvironment

Supplementary Figure 1

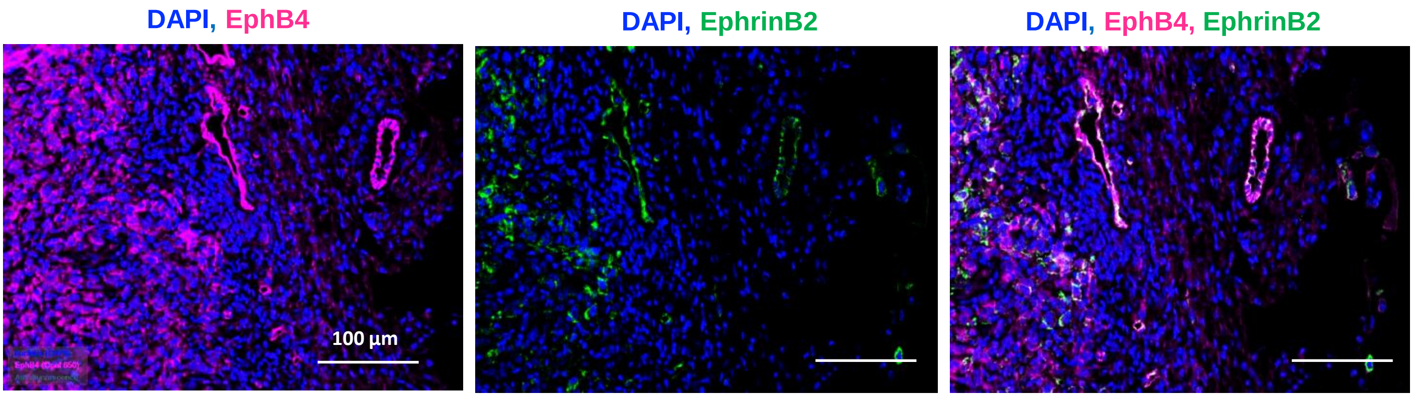

Supplementary Figure 1. Representative **i**mmunofluorescence staining in Moc2 tumor shows the expression of EphB4 and ephrinB2 as single stains and in a composite image. Total magnification: 200x. The analysis was replicated in 2 sets with regions of interest n=5 (EphB4), n=8 (EphrinB2).

Supplementary Figure 2

a

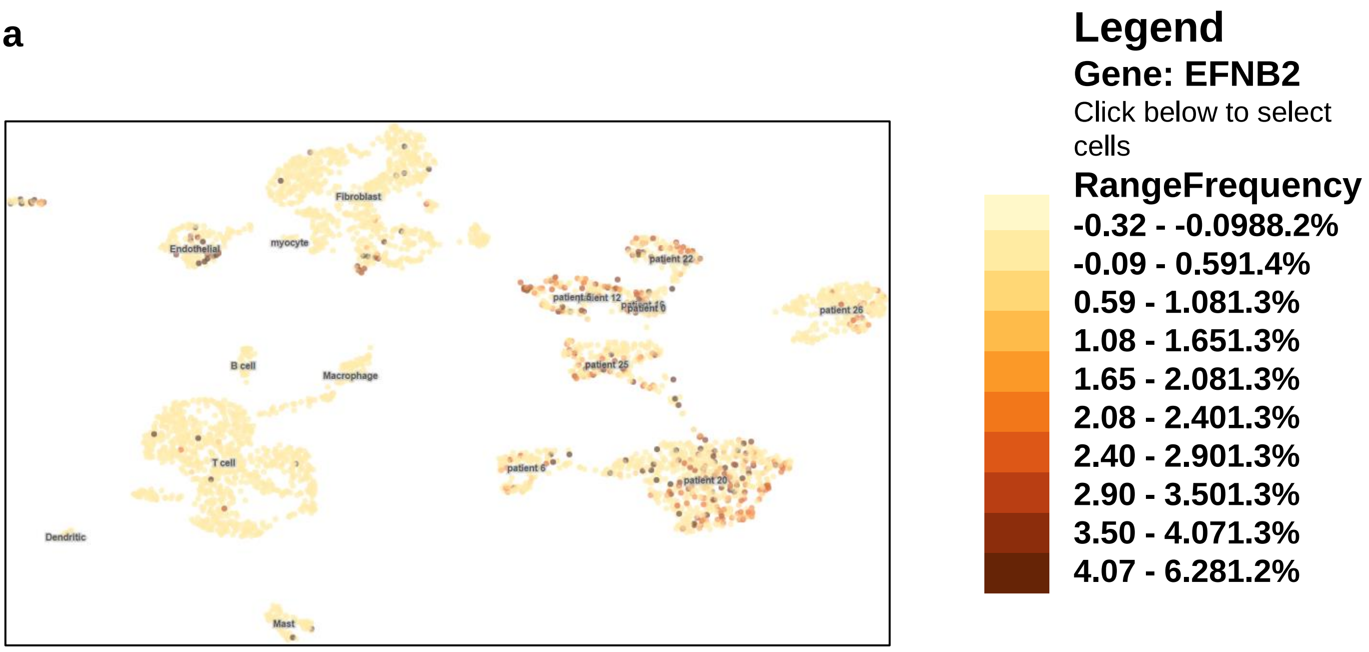

b

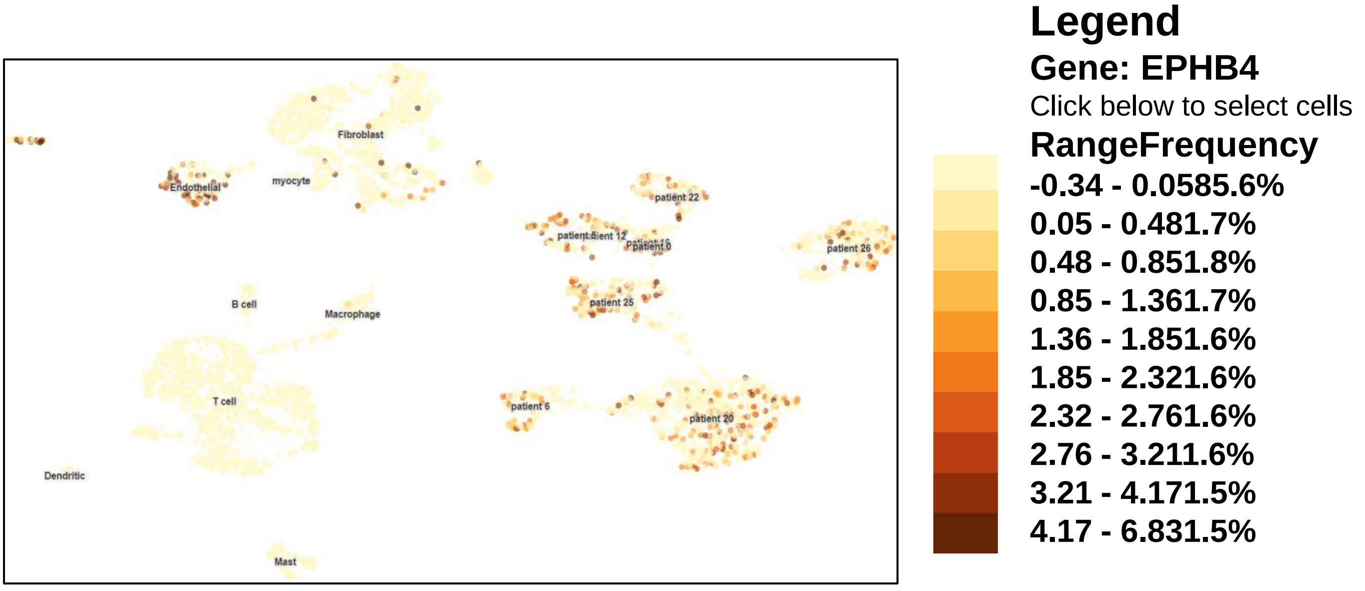

Supplementary Figure 2. Single cell RNA sequencing data analysis on 18 cases of oral cavity head and neck squamous cell carcinoma (HNSCC) patients at the time of surgical resection, either from the primary tumor or lymph node (LN) dissection validates the expression of EFNB2 (a) and EphB4 (b) in different cellular compartments.

Supplementary Figure 3

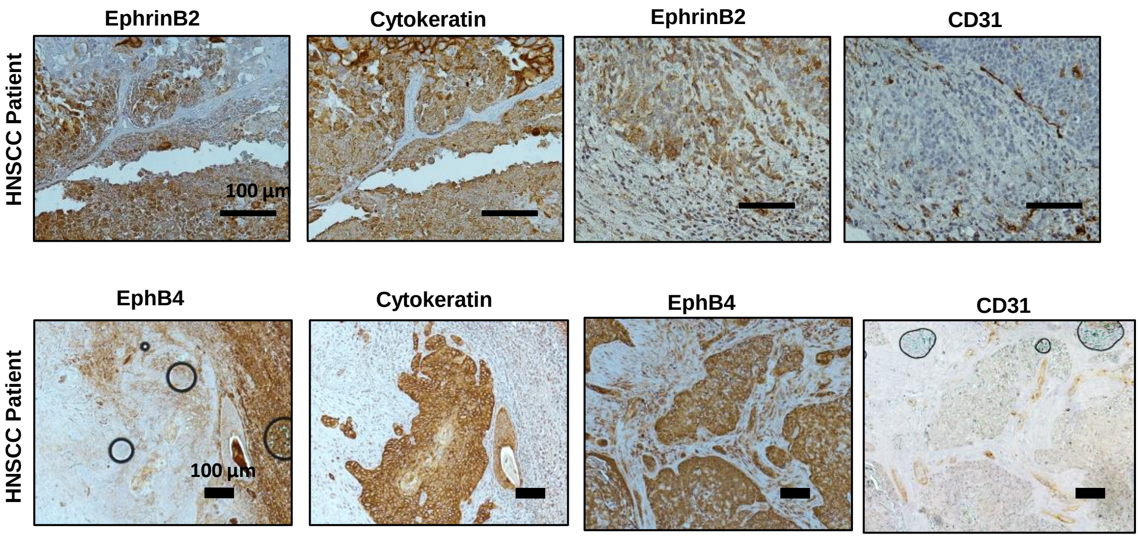

Supplementary Figure 3. Representative immunohistochemical analysis performed on HNSCC patient demonstrates the presence of ephrinB2 and EphB4 on cytokeratin positive tumor epithelial cells and CD31+ vessels. The analysis was done in 3 patients. Total magnification: 200x (top panel); 100x (bottom panel).

Supplementary Figure 4

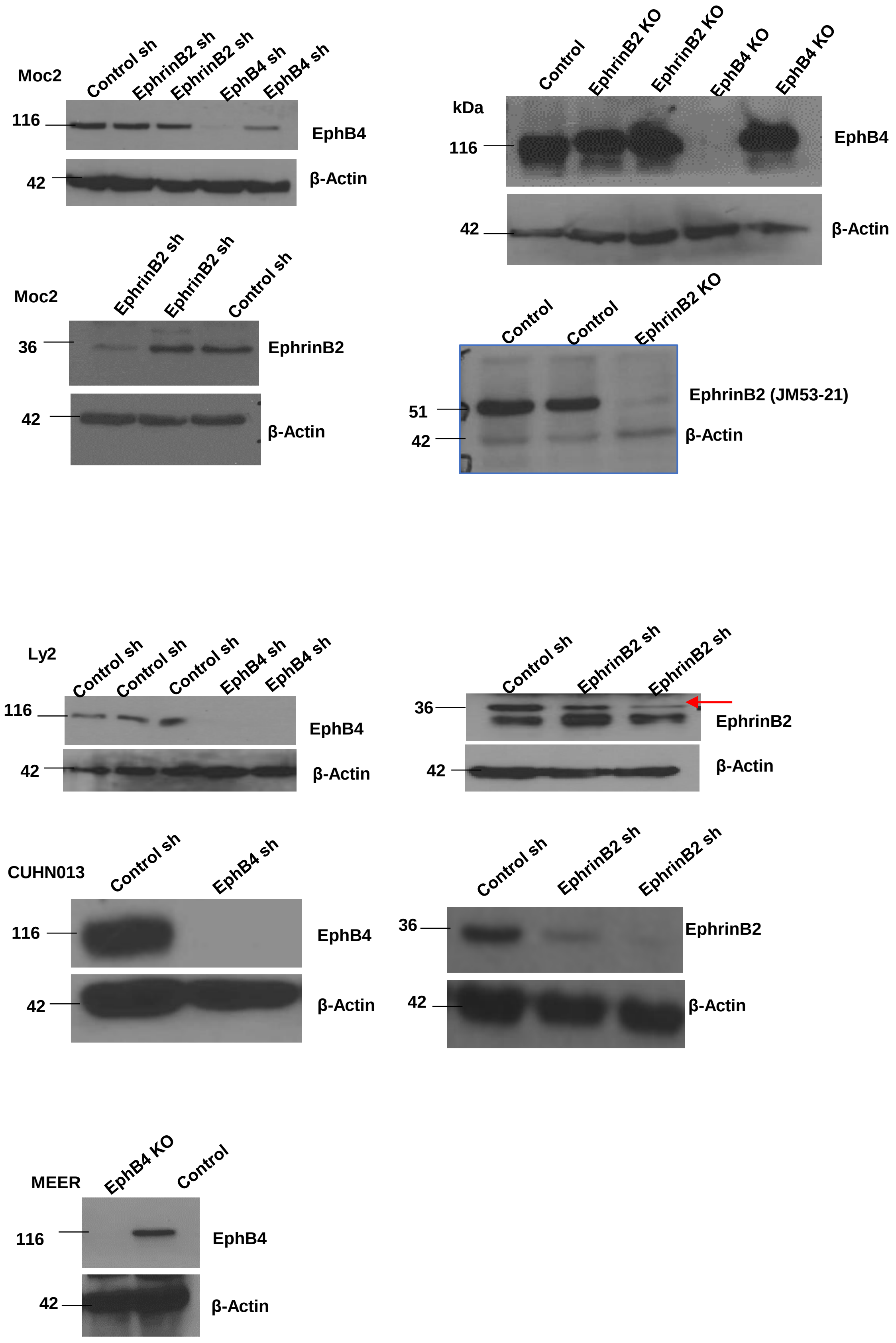

Supplementary Figure 4. Western blot analysis showing knockdown or complete knockout of EphB4 and ephrinB2 in different head and neck cancer cell lines. The experiment was replicated two times.

Supplementary Figure 5

a

Schematic of EphB4/ephrinB2 single gene knockdown or knockout on cancer cell

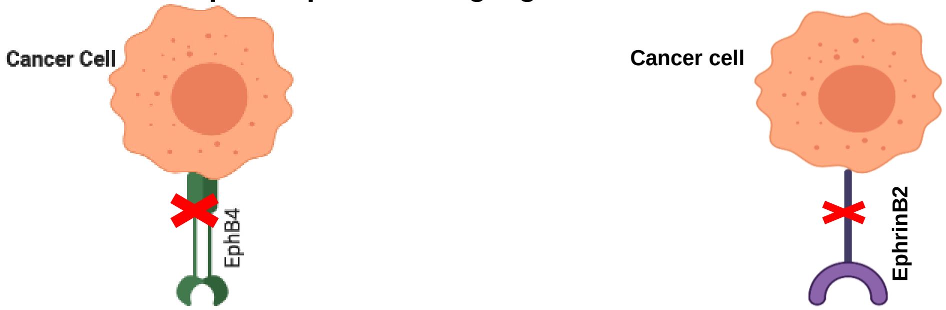

b

Schematic of mouse model with conditional depletion of ephrinB2 on vasculature

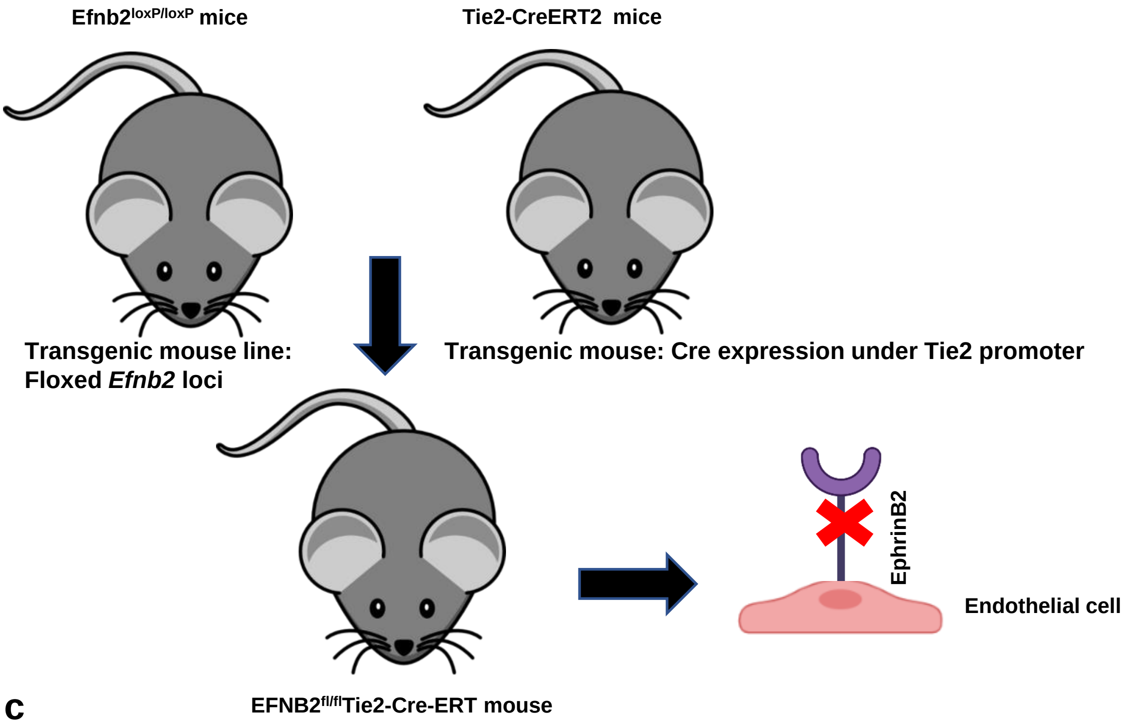

c

Schematic of EphrinB2 knockout on cancer cell and vasculature (referred as ephrinB2 double knockout group)

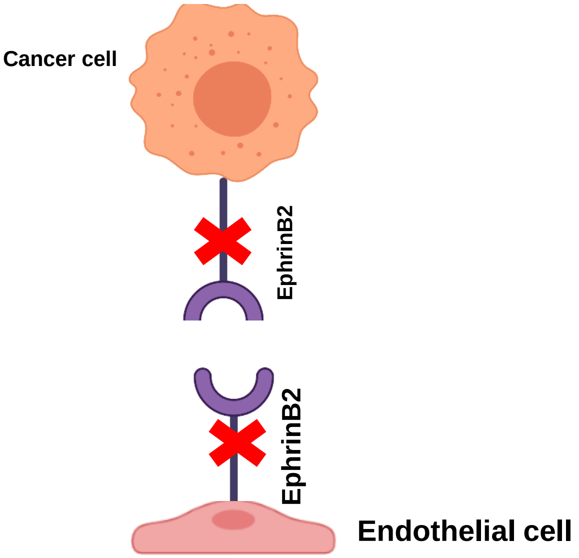

Supplementary Figure 5. Schematic representation of constructs showing (a) single gene knockdown/knockout of EphB4, ephrinB2 on a cancer cell, (b) mouse model showing conditional knockout of ephrinB2 on vasculature, and (c) ephrinB2 double knockout on both the cancer cell and endothelium.

Supplementary Figure 6

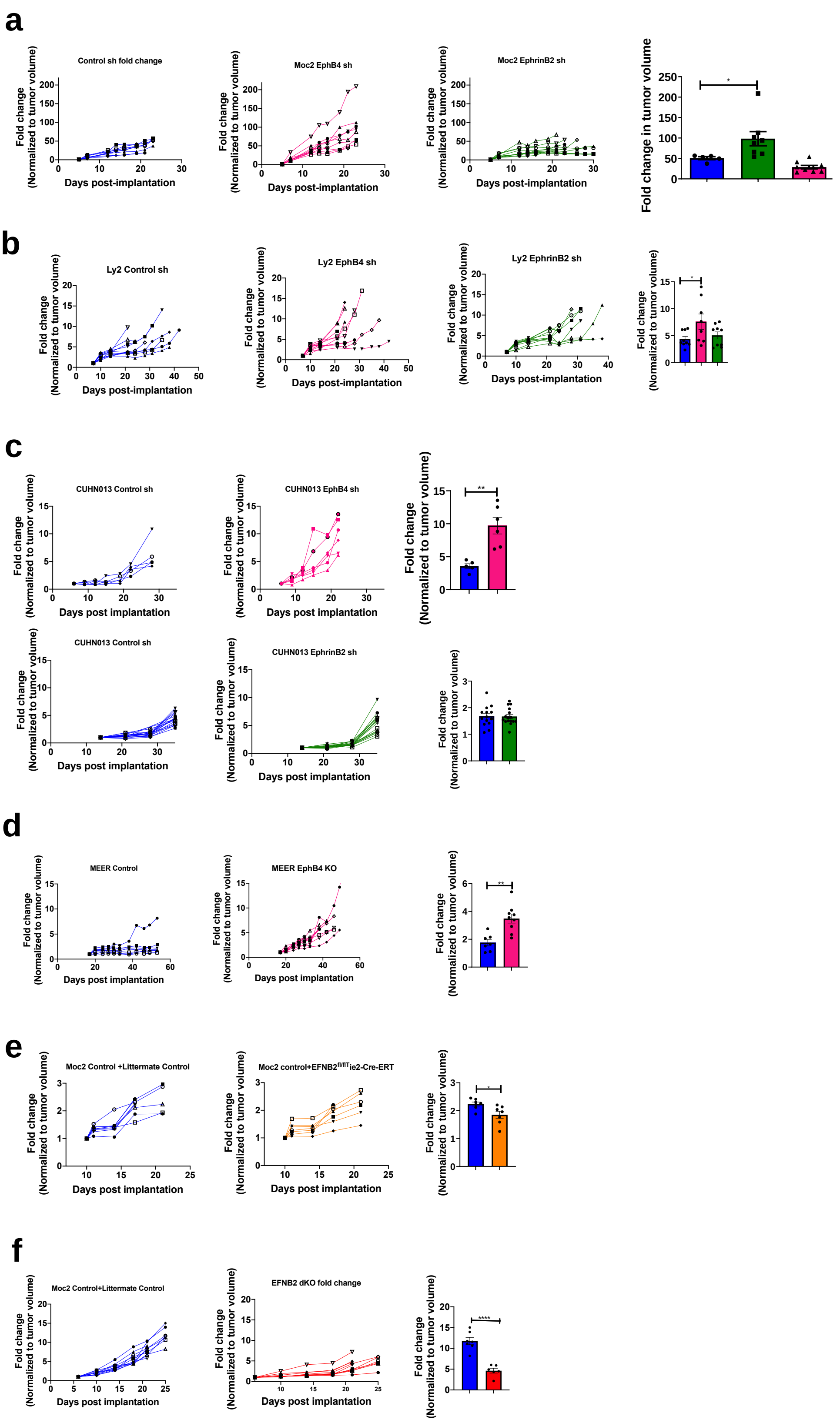

Supplementary Figure 6. Absence of ephrinB2 in both the cancer cells and the vasculature inhibits tumor growth while loss of EphB4 promotes tumor growth progression in orthotopic and xenograft models of HNSCC. Increased tumor growth is observed following knockdown of EphB4 on cancer cells in (a) Moc2 [n=9 (control sh); n=10 (EphrinB2 sh, EphB4 sh], (b) Ly2 (n=10/group), (c) CUHN013 [upper panel: n=6 (control sh); n=6 (EphB4 sh), lower panel: n=16/group] and (d) MEER [n=7 (control); n=10 (EphB4 KO)] models. Tumor volume data are shown in the form of spaghetti plots to present tumor growth of individual mice for the respective groups in a time-dependent manner. The groups in (a-d) are annotated based on the tumor cells implanted in the C57BL/6 mice. (e) Conditional deletion of ephrinB2 on the vasculature impacts Moc2 tumor growth in EFNB2$^{fl/fl}$Tie2-Cre-ERT mice (n=7) to a modest degree. The group annotation refers to the Moc2 control tumors implanted in either littermate controls (left) or EFNB2$^{fl/fl}$Tie2-Cre-ERT mice (right) (f) Loss of ephrinB2 in both the tumor cells and the vasculature [Moc2 ephrinB2 KO + EFNB2$^{fl/fl}$Tie2-Cre-ERT mice (n=10)] results in a maximal decline in tumor growth *in vivo* in a time-dependent manner. The groups correspond to the Moc2 control tumors implanted in littermate controls (left) or Moc2 ephrinB2 KO tumors implanted in EFNB2$^{fl/fl}$Tie2-Cre-ERT mice (right). The experiments were replicated two times. Data are shown as fold change normalized to the raw tumor volumes. Error bars represent mean±SEM. Color key for groups shown in histogram plots is same as depicted in the respective spaghetti plots. Statistical significance was analyzed by performing two-sided Student's *t*-test or ANOVA. The Dunnett *post hoc* test was used after ANOVA where multiple experimental groups were involved. p-values are indicated for figures (a) *p=0.029, (b) *p=0.039, (c) **p=0.002, (d) **p=0.001 (e) *p=0.022 (f) ****p≤0.0001.

Supplementary Figure 7

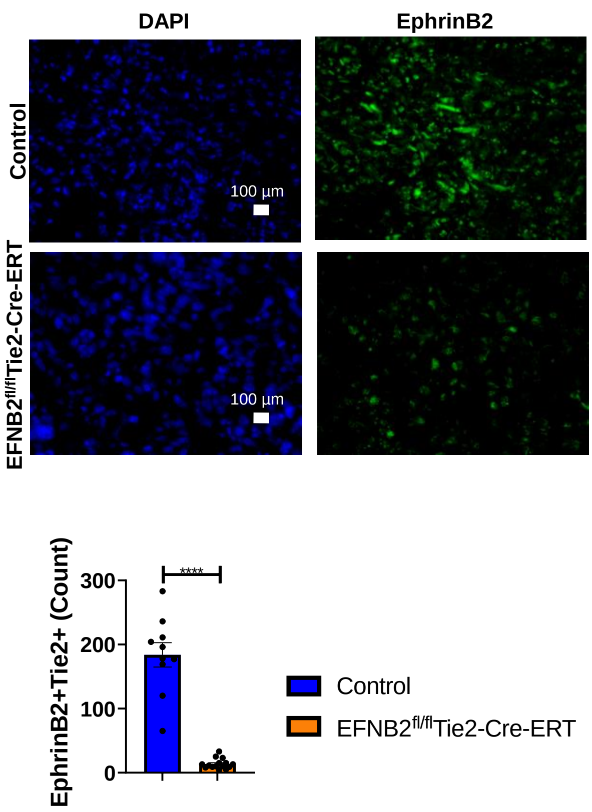

Supplementary Figure 7. Dual immunofluorescence staining confirms the knockout of ephrinB2 on Tie2 expressing cells in Moc2 tumors implanted in the EFNB2$^{fl/fl}$Tie2-Cre-ERT mice (n=15) compared to the controls (n=10). The experiment was performed in two sets. Total magnification: 400x. Data are shown as mean±SEM. Statistical significance was analyzed by performing two-sided Student's *t*-test. ****p≤0.0001.

Supplementary Figure 8

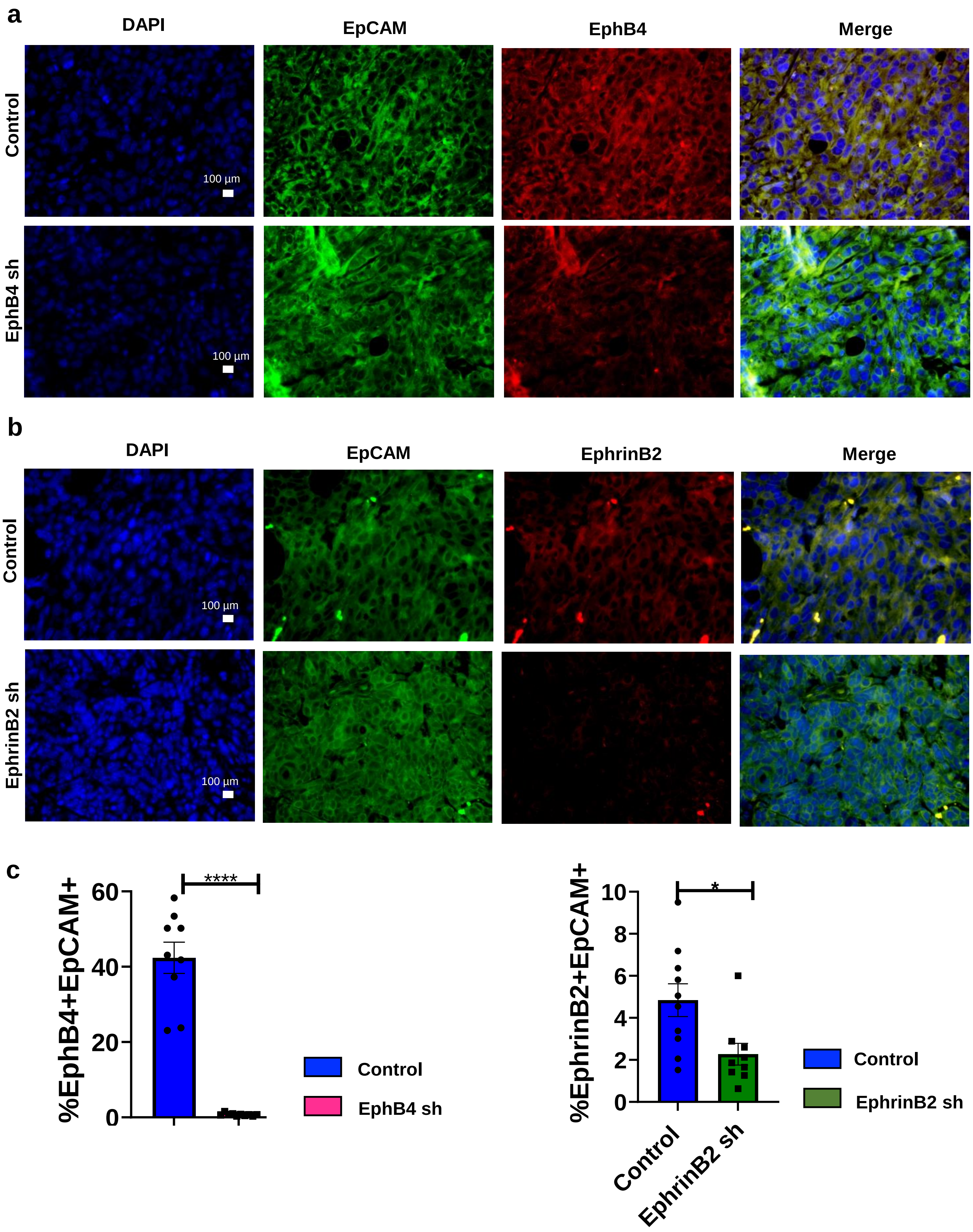

Supplementary Figure 8. Dual immunofluorescence staining confirms the knockdown of EphB4 and ephrinB2 on cancer cells in Moc2 tumors. EphB4 sh and EphrinB2 sh tumors along with control tumors were co-stained with EpCAM and EphB4 (a) or ephrinB2 (b) to confirm the loss of EphB4 or ephrinB2 on the cancer cell. N=2 sets. Total magnification: 400x. Quantitative analysis is shown as histogram plots in (c). Data are shown as mean±SEM. Comparison between the control and experimental groups was done by using two-sided Student's t-test. *p=0.0158, **** $p \leq 0.0001$.

Supplementary Figure 9

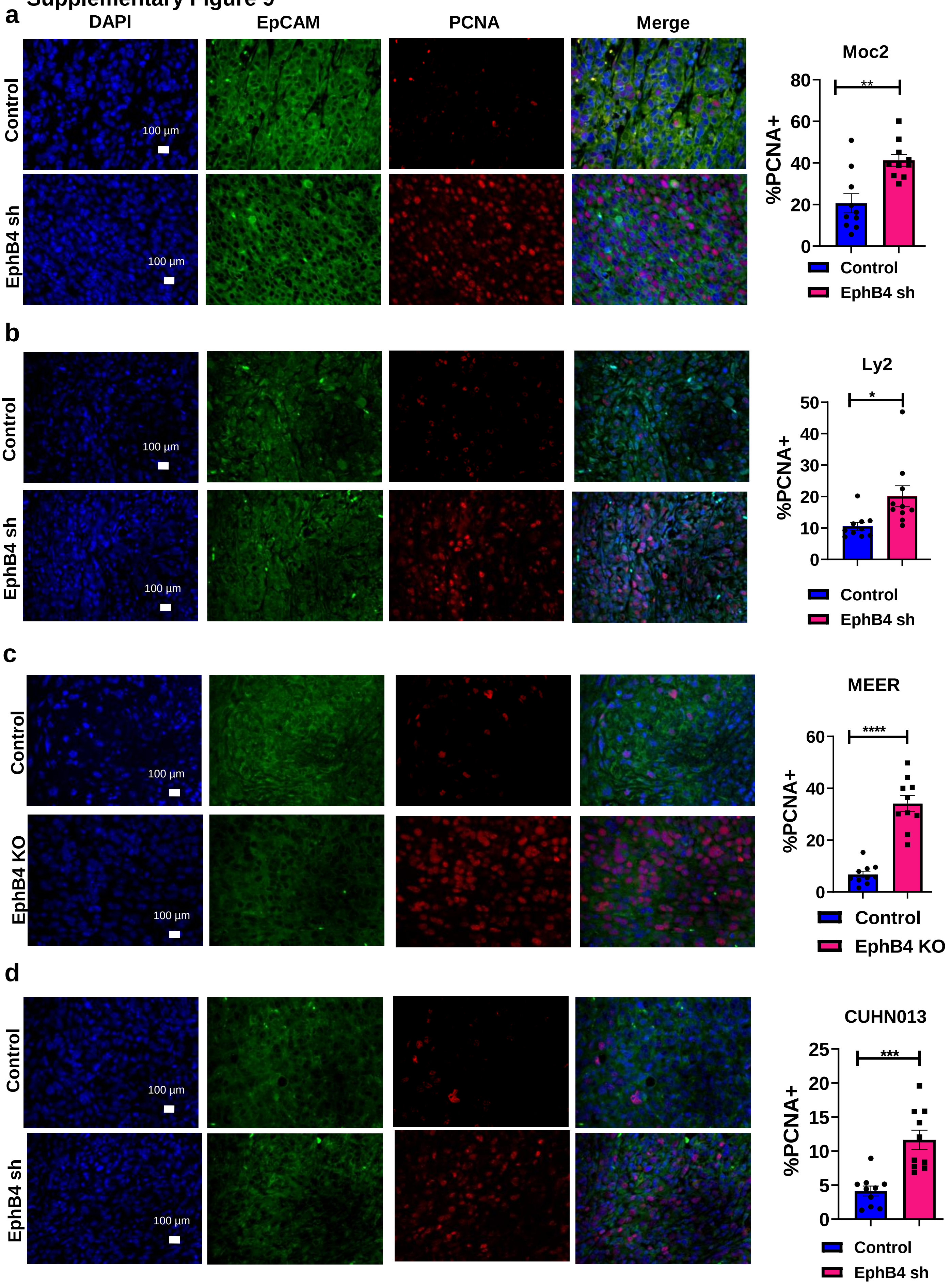

Supplementary Figure 9. Loss of EphB4 on cancer cells results in increased cell proliferation in HNSCC tumors. Control and cancer cell-EphB4 lacking tumors corresponding to Moc2 (a), Ly2 (b), MEER (c), and CUHN013 (d) models (n=2 sets) were co-stained with EpCAM and PCNA to assess epithelial cell proliferation. Quantitative analysis is shown as histogram plots. Total magnification: 400x. Data are shown as mean±SEM. Comparison between control and experimental groups was done by using two-sided Student's t-test. *p=0.015, **p=0.0012, ***p=0.0002, **** p≤0.0001.

Supplementary Figure 10

Schematic of dominant negative constructs

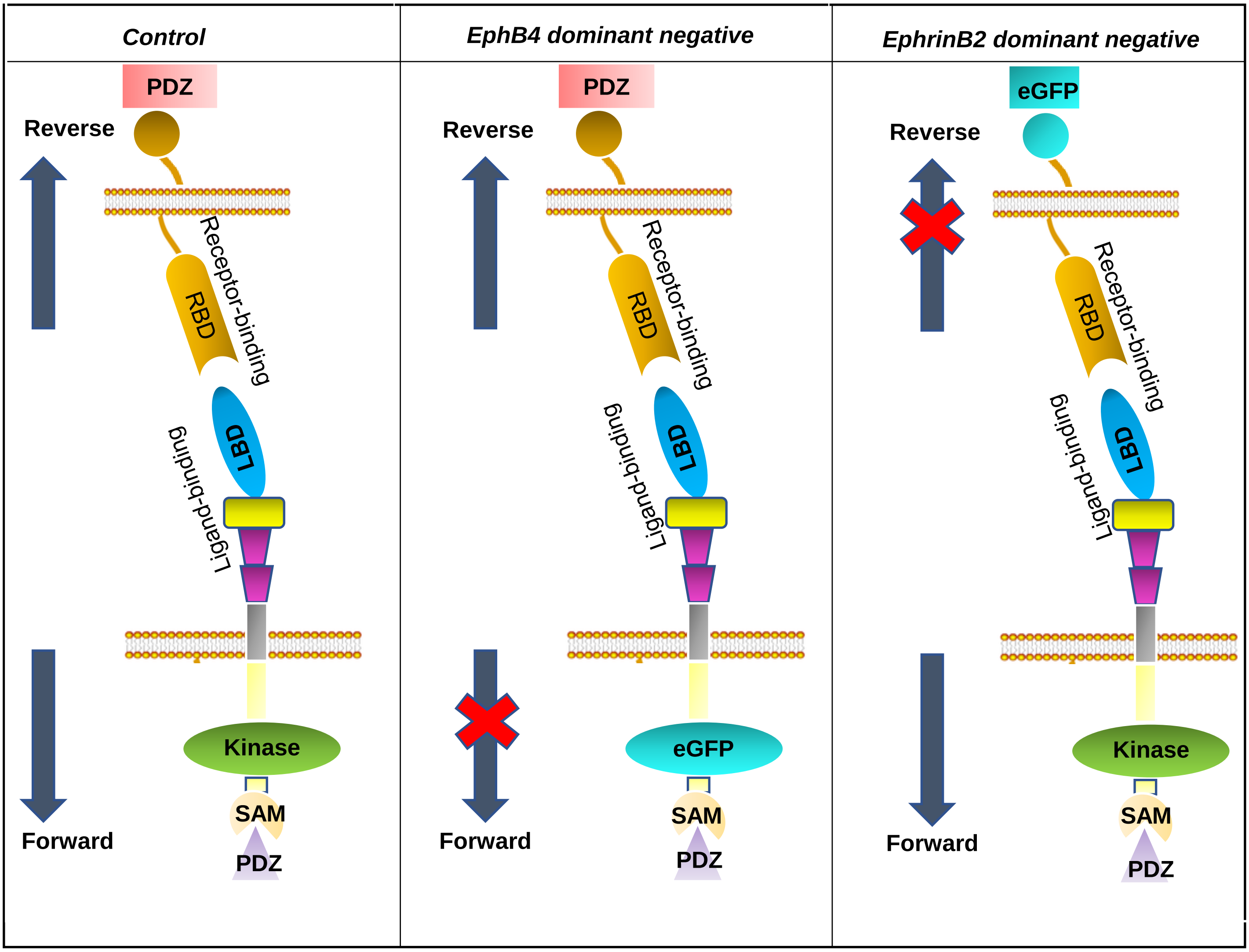

PDZ: post synaptic density protein (PSD95), Drosophila disc large tumor suppressor (Dlg1), and zonula occludens-1 protein (zo-1); RBD: receptor-binding domain; LBD: ligand-binding domain; SAM: sterile alpha motif ; EGFP: enhanced green fluorescent protein

Supplementary Figure 10. Schematic representation of control, EphB4 dominant negative, and ephrinB2 dominant negative constructs.

Supplementary Figure 11

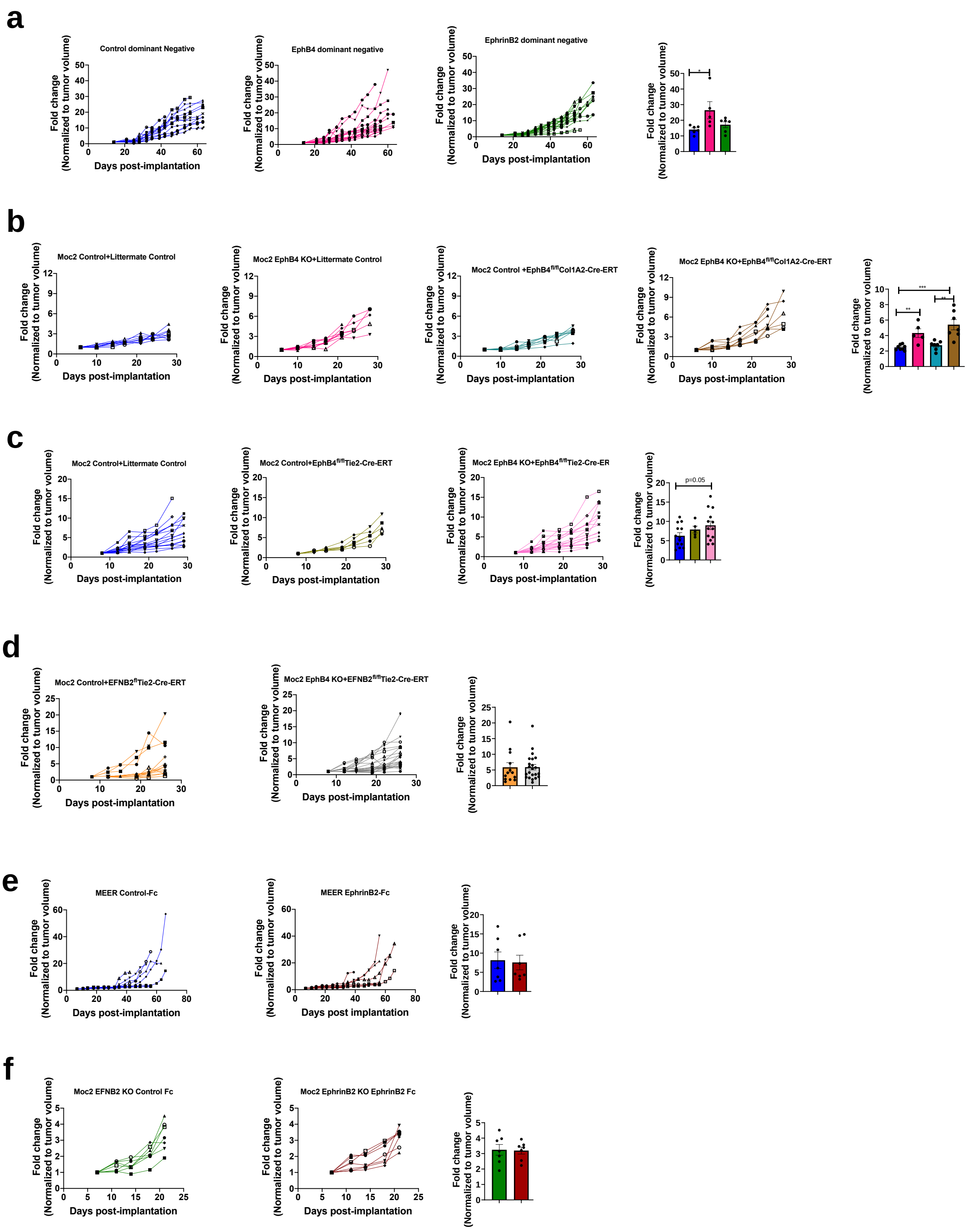

Supplementary Figure 11. Activation of EphB4 on cancer cells in the absence of vascular ephrinB2 fails to reduce tumor growth in different models of HNSCC. (a) Lack of EphB4 intracellular signaling in EphB4 dominant negative constructs enhances tumor growth in a patient-derived xenograft model. CUHN013 HNSCC cells transfected with either control or dominant negative plasmids of EphB4 and ephrinB2 were implanted in the flank region of nude mice [n=6 (control); n=9 (EphB4 dominant negative); n=10 (ephrinB2 dominant negative)], and tumor growth was observed in a time-dependent manner. The groups are annotated based on the tumor cells implanted in the respective mice. Absence of EphB4 in collagen I-expressing cells such as fibroblasts in EphB4$^{fl/fl}$Col1A2-Cre-ERT mice (n=6) (b) or in adult vasculature in EphB4$^{fl/fl}$Tie2-Cre-ERT mice (n=8) (c) fail to significantly impact the tumor growth as compared to the littermate controls (n=8). (d) Implantation of EphB4 KO tumor cells in EFNB2$^{fl/fl}$Tie2-Cre-ERT mice with conditional loss of ephrinB2 on the vascular endothelial cells did not achieve tumor growth suppression. The groups in figures b-d are annotated in the format: “tumor name+mouse strain”. Lastly, tumor growth data is shown in MEER control (n=8) (e) and Moc2 ephrinB2 KO (n=7) (f) tumor models where systemic administration of ephrinB2-Fc to activate EphB4 receptor failed to achieve tumor growth reduction in EFNB2$^{fl/fl}$Tie2-Cre-ERT mice. For figures e-f, groups are annotated based on the tumor cells implanted followed by Fc treatment. Except b-d, other experiments are replicated two times. Data are shown as fold change and represent mean±SEM. Color key for groups shown in histogram plots is same as depicted in the respective spaghetti plots. Statistical significance was analyzed by performing two-sided Student’s *t*-test or ANOVA. p-values are indicated for the figures (a) *p=0.03 (b) blue vs pink bar **p=0.0019; blue vs brown bar ***p=0.0004; teal vs brown bar **p=0.002.

Supplementary Figure 12

a

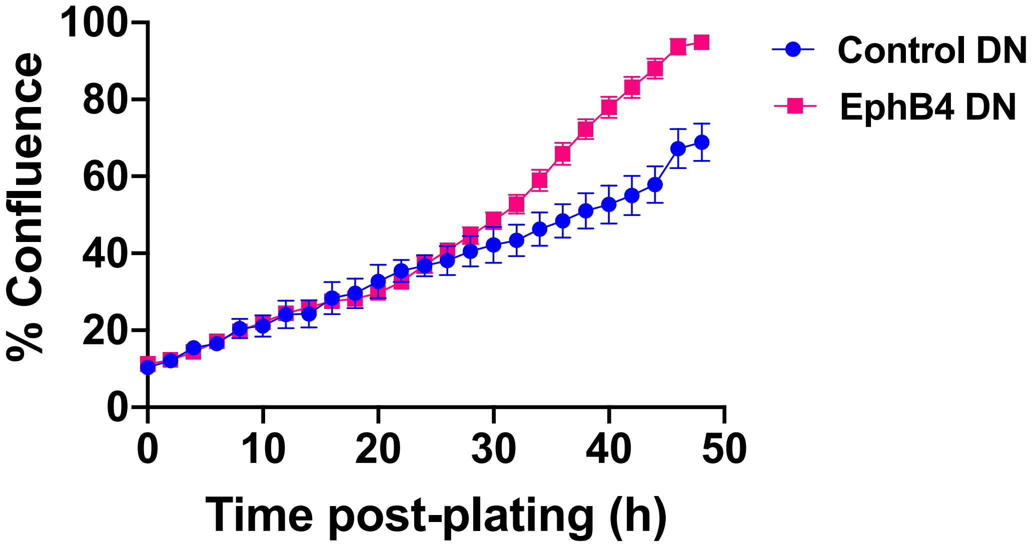

b

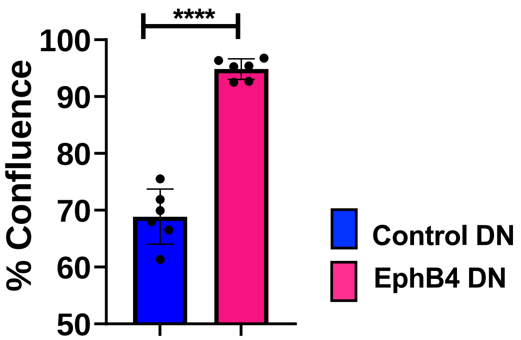

Supplementary Figure 12. (a) IncuCyte assay showing an increase in cell growth in CUHN013 EphB4 dominant negative (DN) cells (n=6) in real-time compared to the control group (n=6). (b) A significant difference in cell growth as depicted by percent confluence is observed at 48 h between the control (n=6) and the EphB4 dominant negative (DN) (n=6) groups. Data are shown as mean±SD. Statistical significance was analyzed by performing two-sided Student's *t*-test. ****p ≤0.0001. The experiment was replicated two times.

Supplementary Figure 13

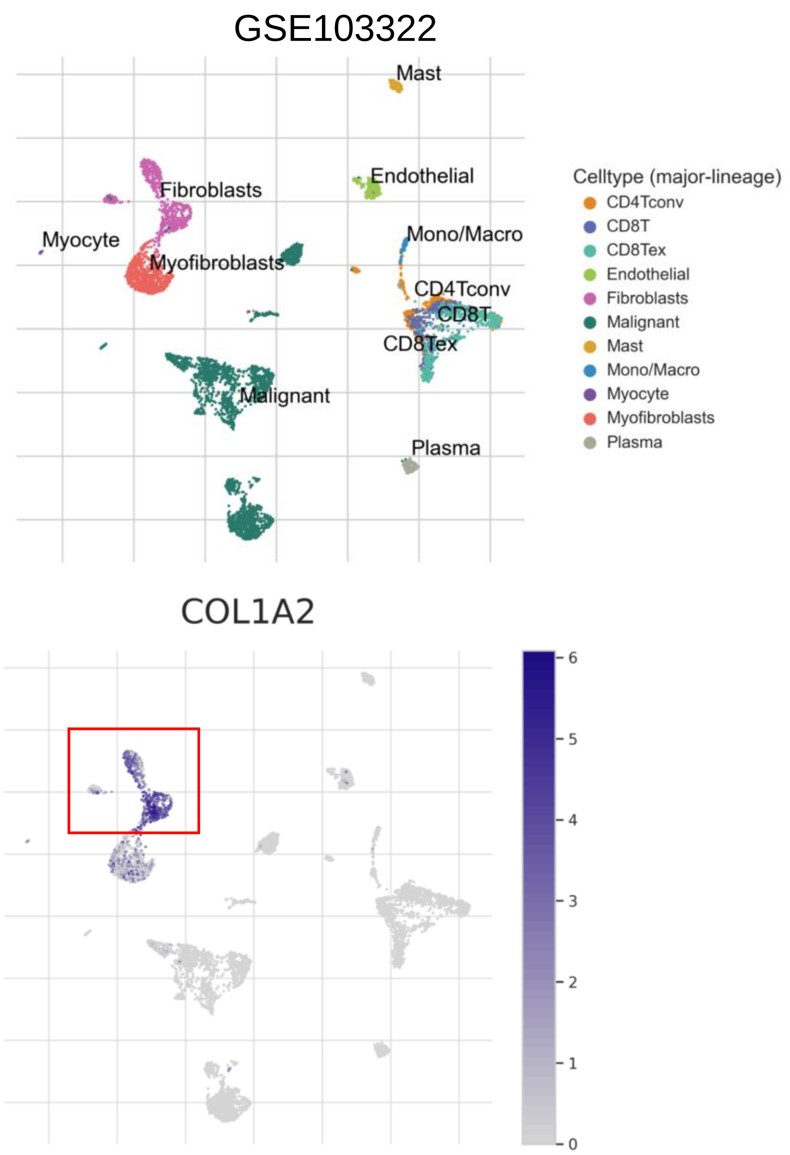

Supplementary Figure 13. Single cell RNA sequencing data analysis on head and neck squamous cell carcinoma (HNSCC) patients (Dataset: GSE103322) validates the expression of Col1A2 on fibroblasts. The data was interrogated using http://tisch.comp-genomics.org browser.

Supplementary Figure 14

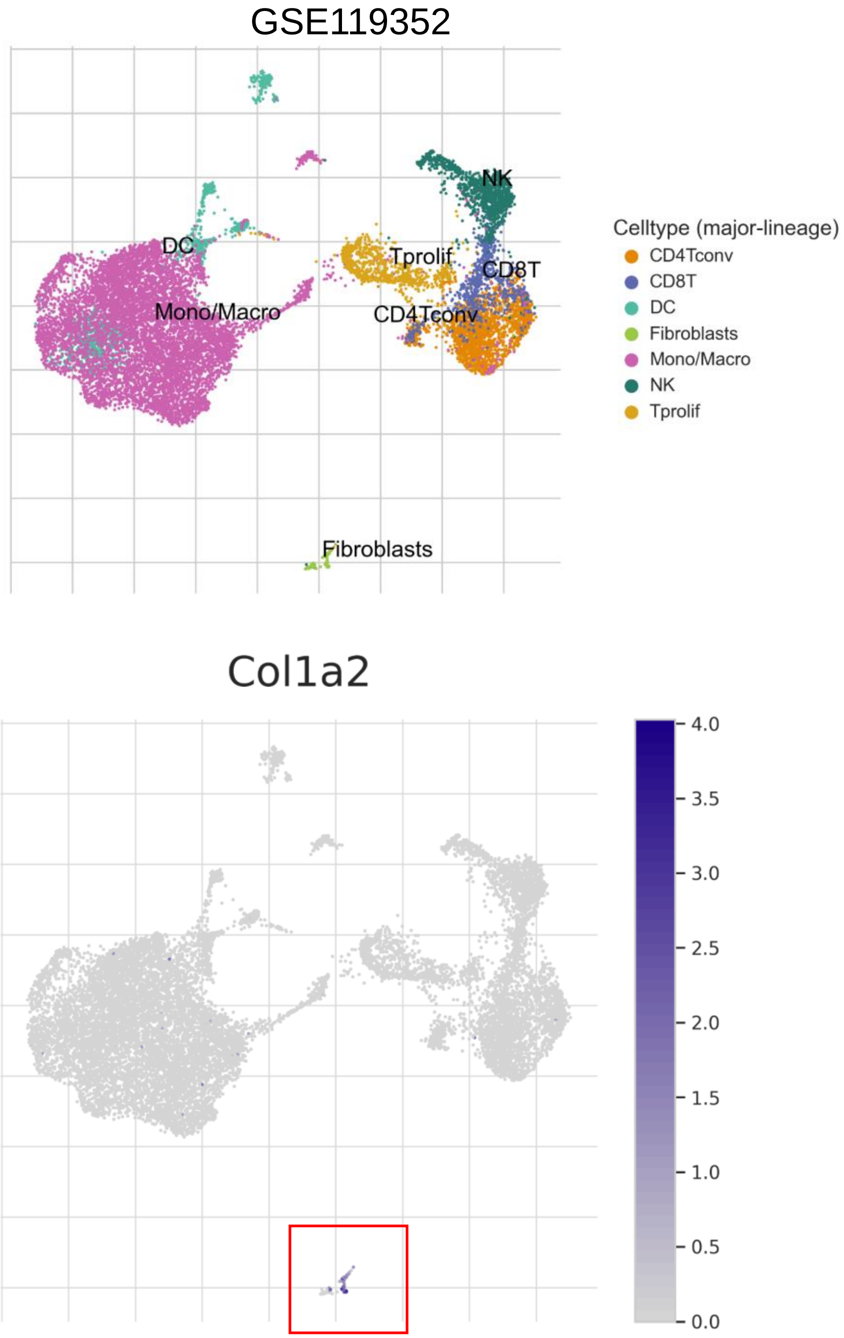

Supplementary Figure 14. Single cell RNA sequencing data analysis on murine dataset (Dataset: GSE119352) confirms the presence of Col1A2 on fibroblasts. The data was interrogated using http://tisch.comp-genomics.org browser.

Supplementary Figure 15

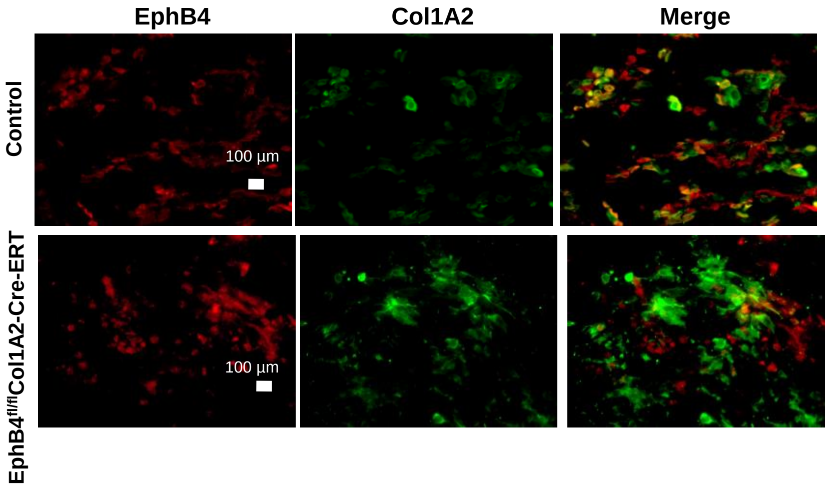

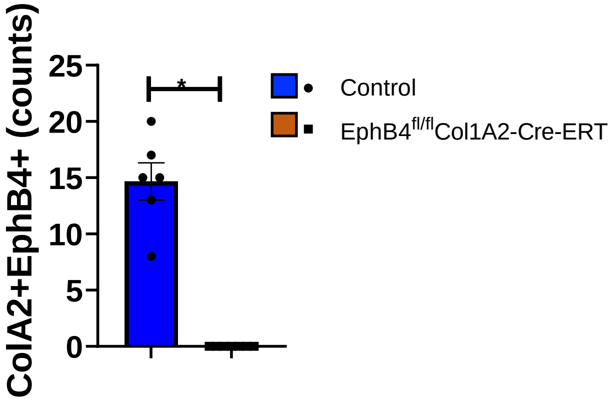

Supplementary Figure 15. Dual immunofluorescence staining confirms the absence of EphB4 on Col1A2 expressing cells in the EphB4$^{fl/fl}$Col1A2-Cre-ERT mice (n=6)**.** Total magnification: 400x. The experiment was performed in two sets. Data are shown as fold change and error bars mean±SEM. Statistical significance was analyzed by performing two-sided Student's *t*-test. *p=0.0114.

Supplementary Figure 16

Schematic showing activation of EphB4 receptor using recombinant ephrinB2-Fc protein

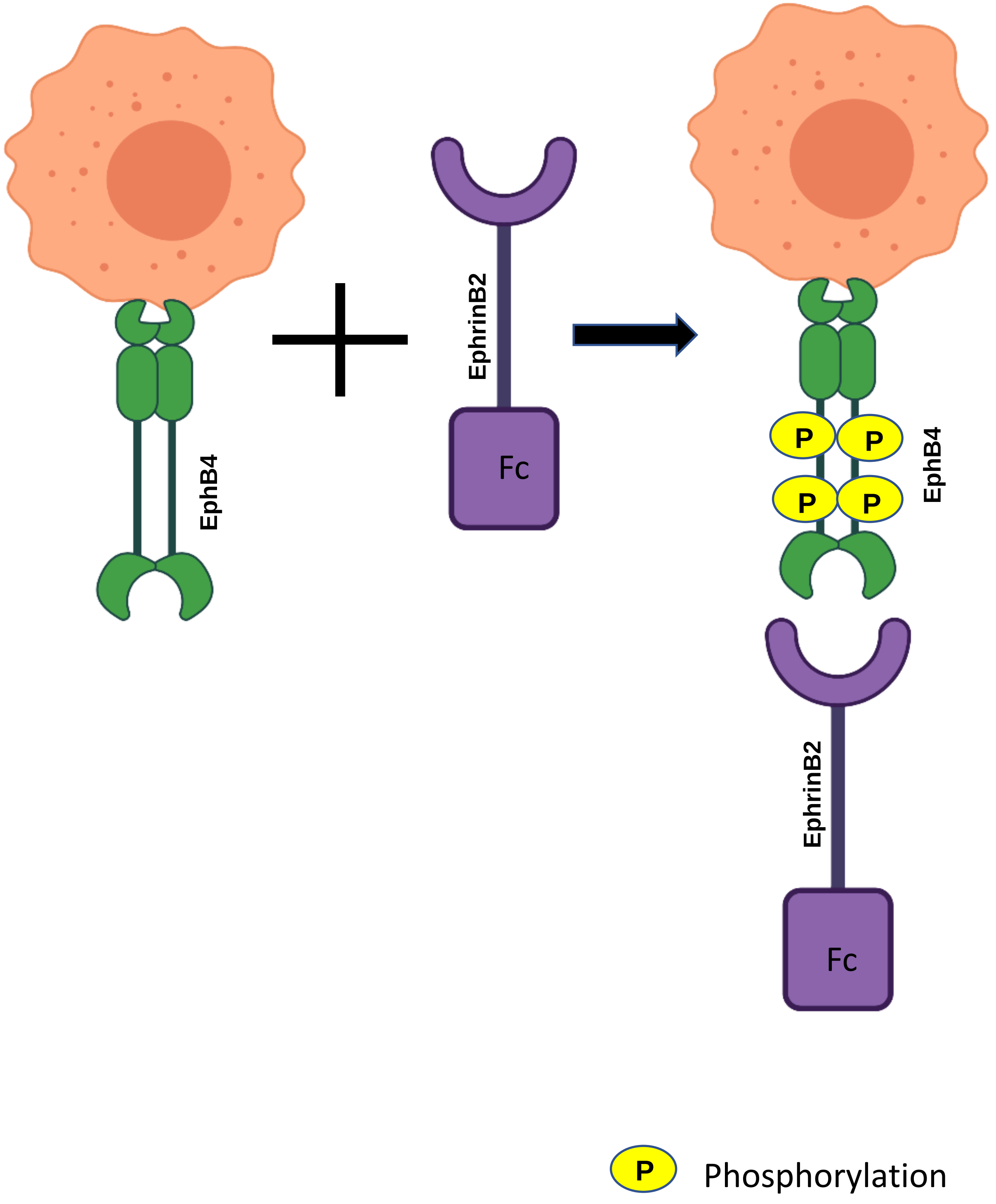

Supplementary Figure 16. Schematic showing the use of recombinant ephrinB2-Fc protein to activate the EphB4 receptor forward signaling.

Supplementary Figure 17

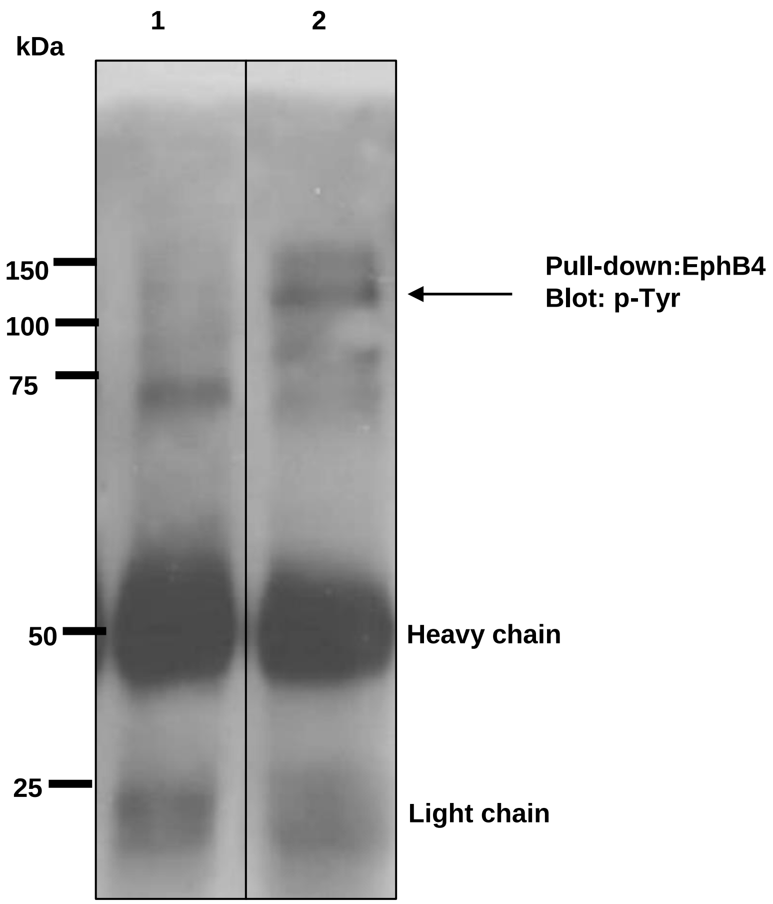

1: Moc2 EphrinB2 KO + Control Fc
2: Moc2 EphrinB2 KO + EphrinB2 Fc

Supplementary Figure 17. An immunoprecipitation assay showed an increase in p-EphB4 levels in Moc2 ephrinB2 KO tumors treated with recombinant ephrinB2 Fc protein. The experiment was performed two times.

Supplementary Figure 18

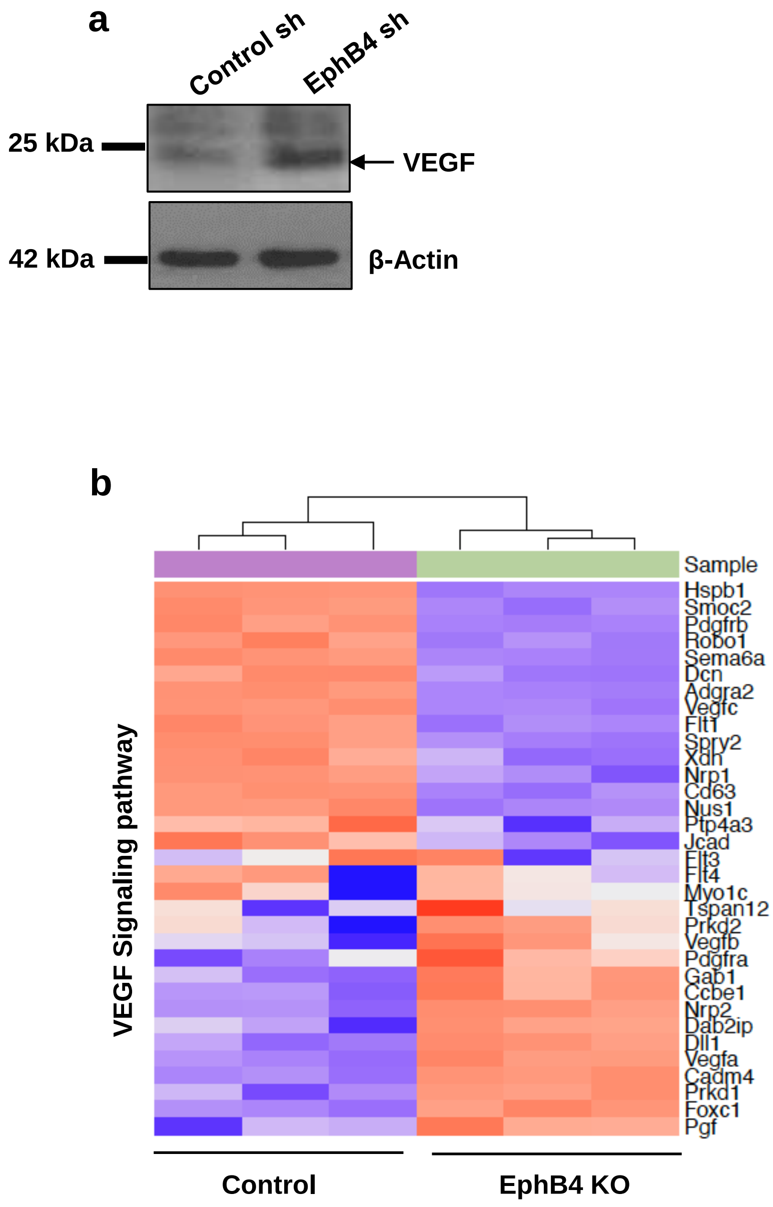

Supplementary Figure 18. Loss of EphB4 on cancer cells results in alteration of genes in the VEGF signaling pathway. Absence of EphB4 on cancer cells results in upregulation of VEGF as determined by western blot analysis (a). The experiment was performed two times. RNA-seq analysis performed on the Moc2 EphB4 KO cancer cell line vs control shows transcriptional changes in the VEGF signaling pathway (b).

Supplementary Figure 19

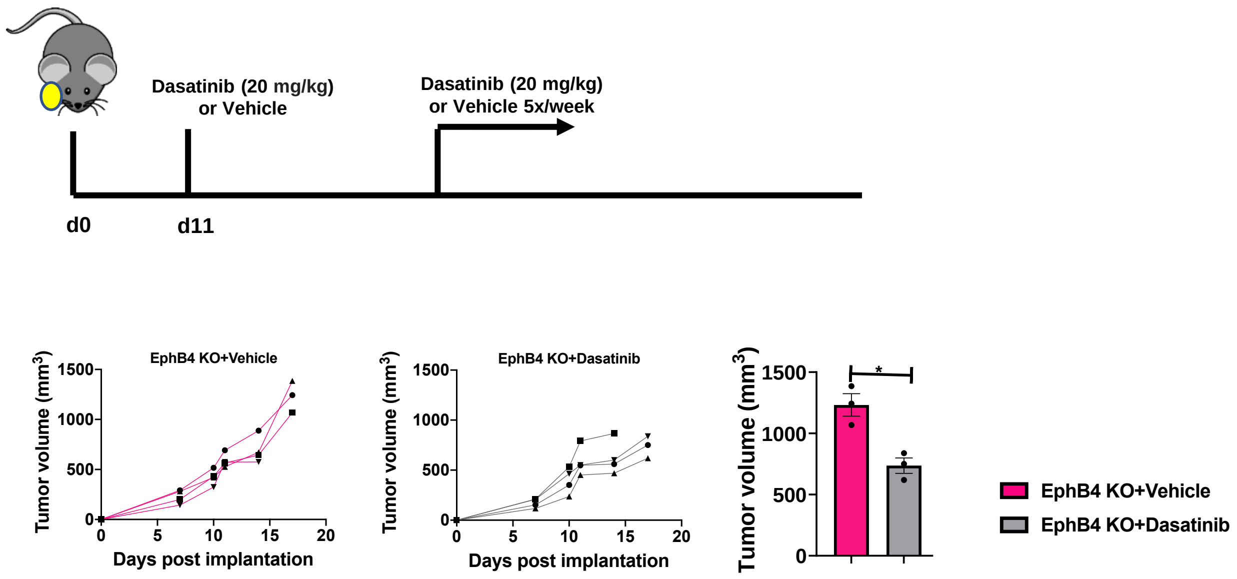

Supplementary Figure 19. Targeting EphA4 by broad-activity tyrosine kinase inhibitor reverses the accelerated tumor growth in EphB4 KO tumor-bearing mice. EphB4 knockout tumor cells were implanted in the buccal region of mice (n=4) followed by treatment with Dasatinib once the tumors reached a volume of ~150 mm^3. The groups are annotated in the format: “tumor name+treatment”. Histogram plot shows significant decrease in inhibitor treated versus control group at day 17 post-tumor implantation. The experiment was performed once with its own biological replicates. Data are shown as mean±SEM. Statistical significance was analyzed by performing two-sided Student’s *t*-test. *p=0.011.

Supplementary Figure 20

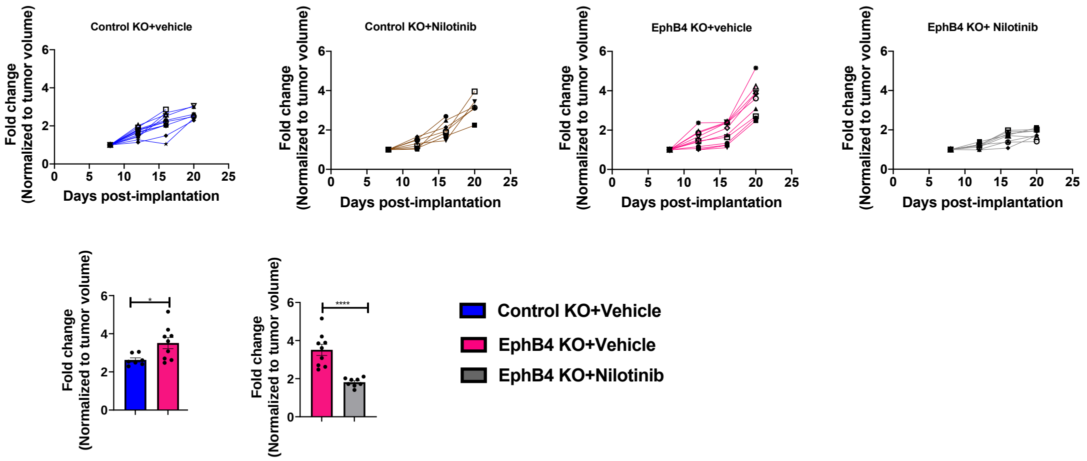

Supplementary Figure 20. Treatment of Moc2 EphB4 KO tumors with Nilotinib results in significant reduction in tumor growth. Histogram plot shows significant tumor growth reduction in inhibitor treated (n=8) versus control group (n=12). at day 20 post-tumor implantation. The groups are annotated in the format: "tumor name+treatment". The experiment was performed once with its own biological replicates. Data are shown as fold change in tumor volumes. Error bars represent mean±SEM. Statistical significance was analyzed by performing two-sided Student's *t*-test. *p=0.023, ****p ≤0.0001.

Supplementary Figure 21

a

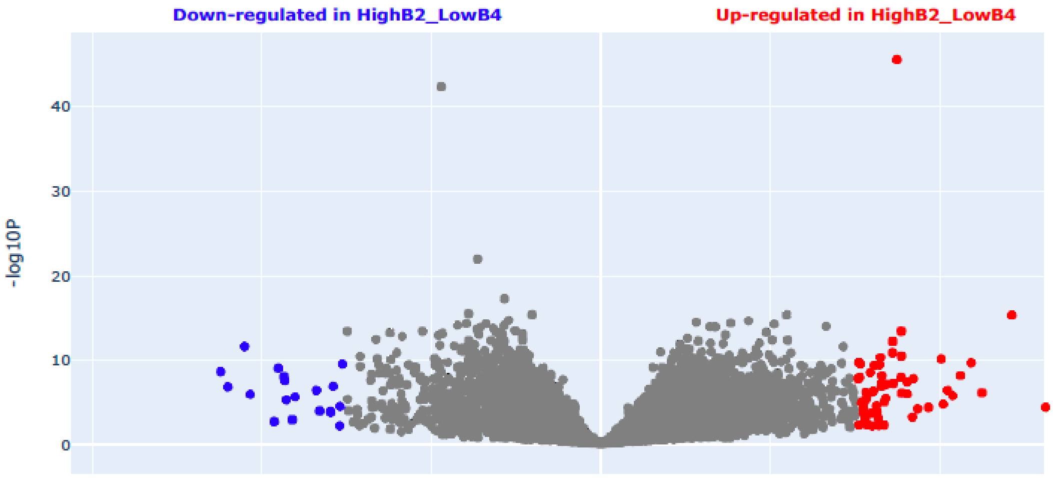

b

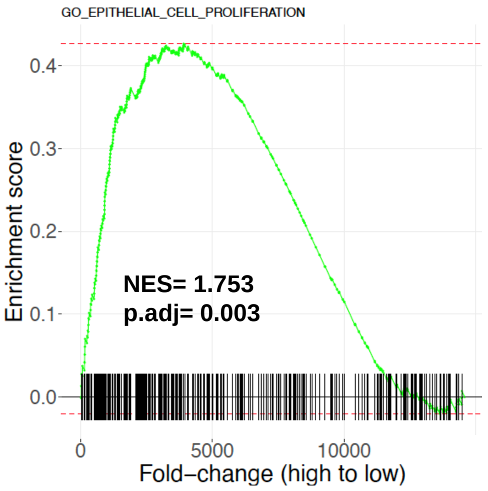

c

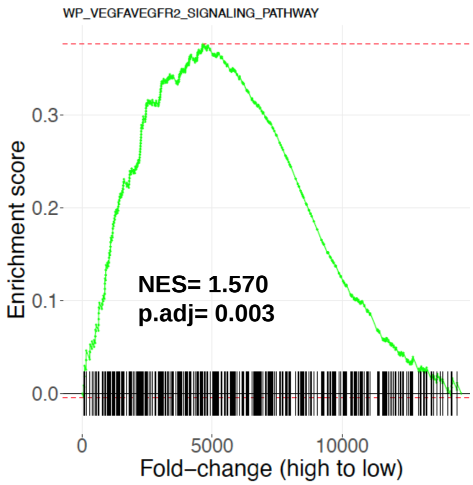

d

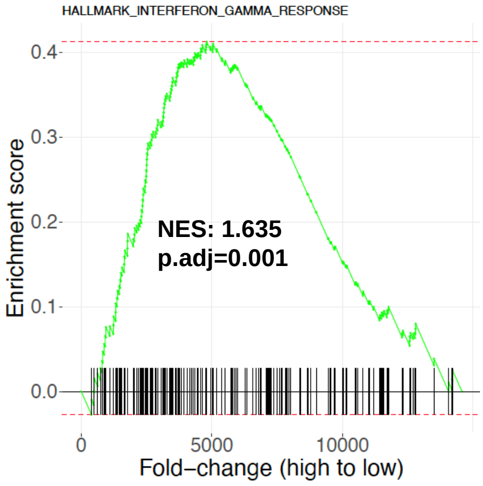

Supplementary Figure 21. (a) Volcano plot showing alteration in genes with high EFNB2-low EPHB4 (n=117) vs low EFNB2-high EPHB4 (n=116) levels in TCGA HNSCC patients. The figure displays a scatter plot showing the log2-fold changes determined by performing a differential gene expression analysis. Genes with logFC >1.5 and p-value < 0.05 are shown in red and genes with logFC < -1.5 and p-value < 0.05 are shown in blue. Enrichment curves for GO_Epithelial cell proliferation (b), WikiPathway_VEGFAVEGFR2 signaling (c), and Hallmark Interferon gamma response pathway (d) are shown for TCGA HNSCC patients with high EFNB2-low EPHB4 expression vs low EFNB2-high EPHB4 expression. The padj value is calculated by The Benjamini-Hochberg (BH) method.

Supplementary Figure 22

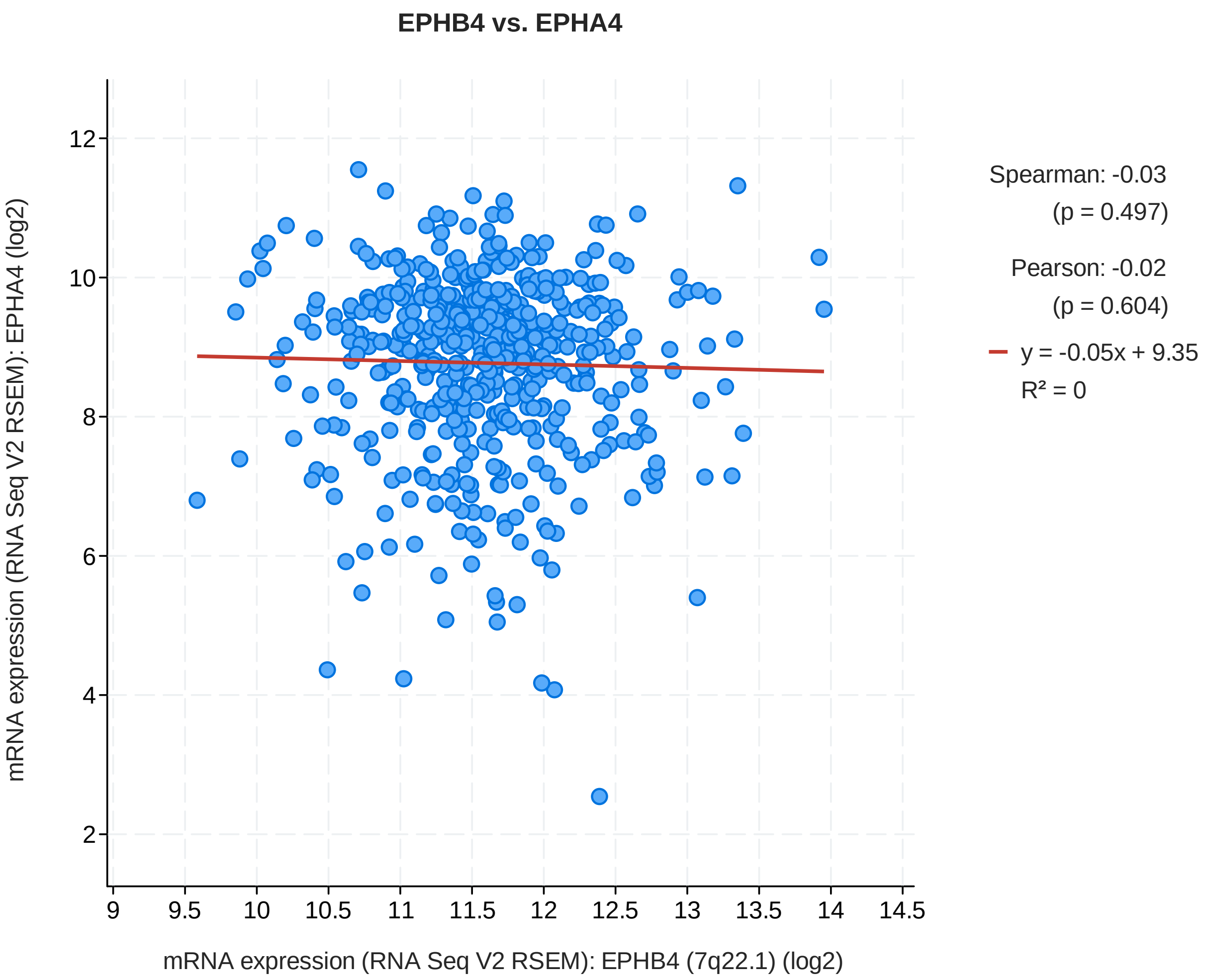

Supplementary Figure 22. TCGA HNSCC patient dataset (n=522 patients) was interrogated to find a correlation between EPHA4 and EPHB4 expression.

Supplementary Figure 23

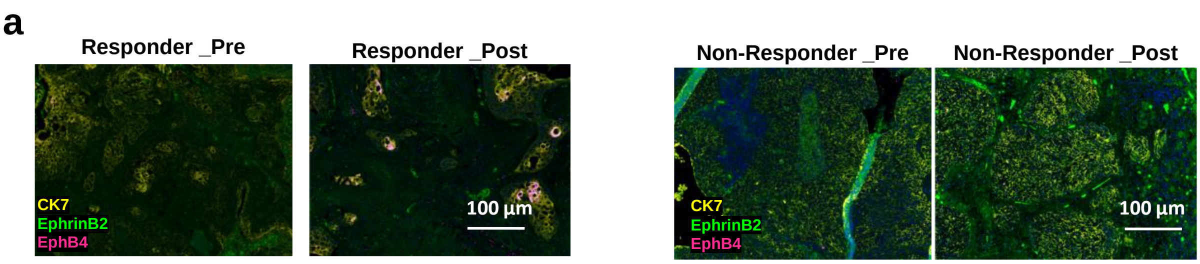

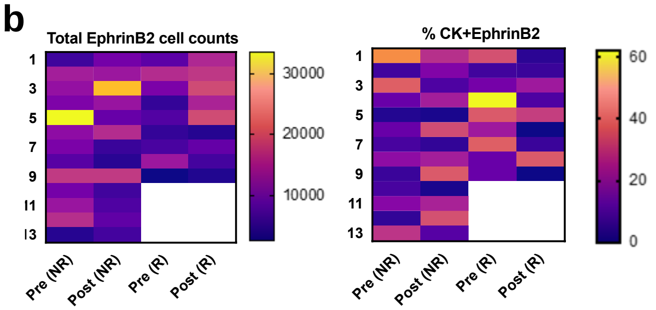

	High EphrinB2 (% patients) Non-Responders	Low EphrinB2 (% patients) Non-Responders	High EphrinB2 (% patients) Responders	Low EphrinB2 (% patients) Responders
Total EphrinB2 Cell counts	38.46	61.53	77.77	22.22
% CK7+ EphrinB2	53.84	46.15	22.22	77.77

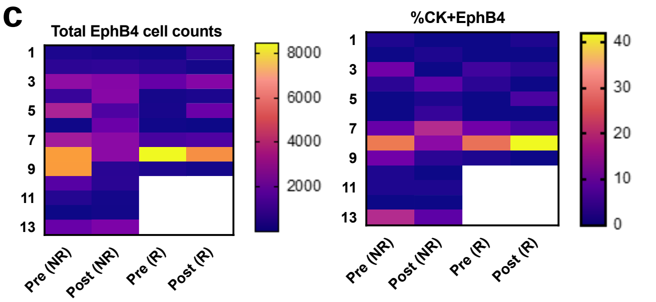

	High EphB4 (% patients) Non-Responders	Low EphB4 (% patients) Non-Responders	High EphB4 (% patients) Responders	Low EphB4 (% patients) Responders
Total EphB4 Cell counts	38.46	61.53	55.5	44.4
% CK7+ EphB4	53.84	30.76	44.44	33.33

Supplementary Figure 23. High EPHB4-low EFNB2 might be helpful to determine response to cetuximab therapy. (a) Representative VECTRA images are shown for the responder and non-responder HNSCC patients treated with cetuximab therapy. Total magnification: 200x. (b) Total ephrinB2 and %CK7+ ephrinB2 as well as (c) total EphB4 cell counts and %CK7+EphB4 was analyzed in all the responders (n=9) and non-responders (n=13) pre-and post-cetuximab treatment and data is represented in the form of heat maps (b and c) and in tabular format. These tables include information on percentage of patients that showed high/low levels of ephrinB2 (right panel b) and EphB4 (right panel c) post-treatment to cetuximab.

Supplementary Figure 24

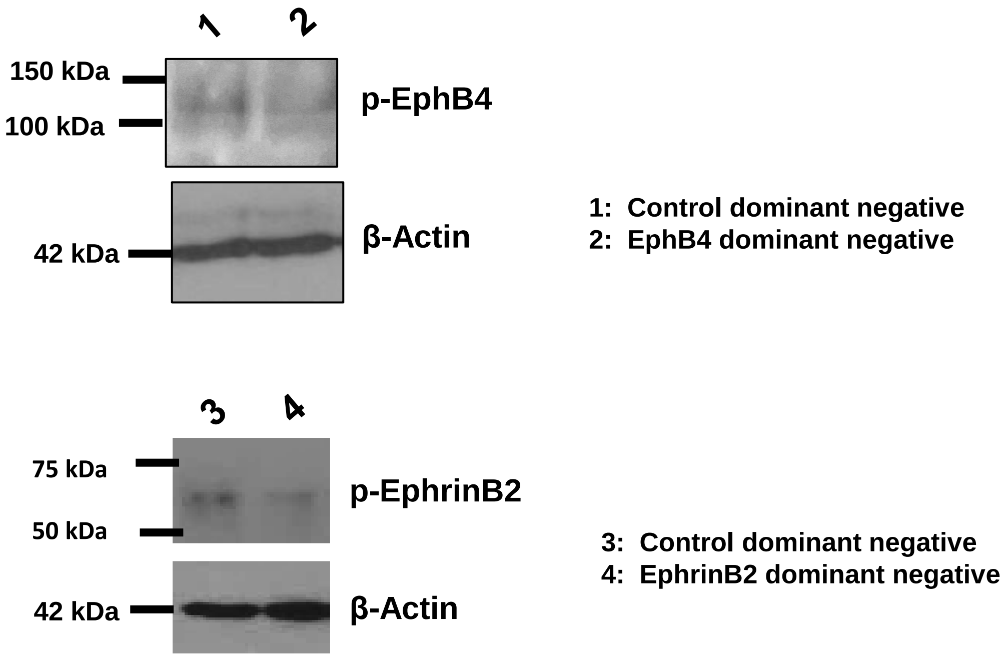

Supplementary Figure 24. Phosphorylated levels of p-EphB4 and p-ephrinB2 are shown in the representative CUHN013 dominant negative tumors by immunoprecipitation assay or western blotting. The experiment was performed two times.

Supplementary Figure 25

a EFNB2$^{fl/fl}$Tie2-Cre-ERT

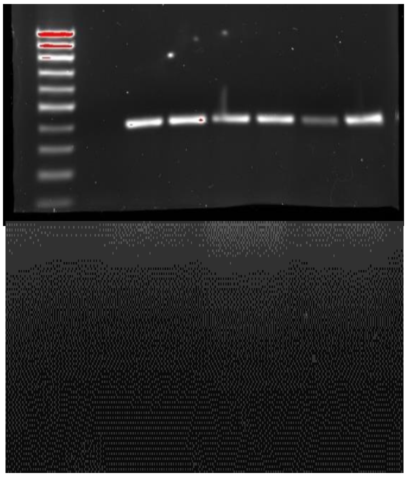

Tie2-Cre (450 bp)

Lane 1: Negative Control
Lanes 2-7: Genotyped samples

EFNB2 f/f (552 bp)

Lane 1: Negative Control
Lanes 2-7: Genotyped samples

b EphB4$^{fl/fl}$Tie2-Cre-ERT

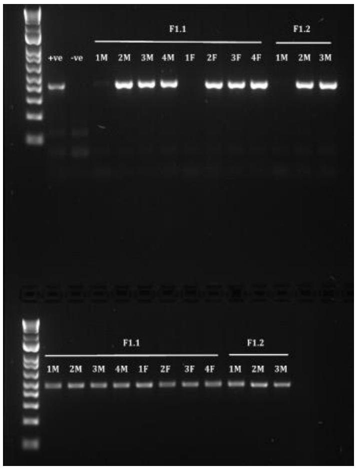

Tie2-Cre (450 bp)

Lane 1: Positive Control
Lane 2: Negative Control
Lanes 3-13: Genotyped samples

EphB4 f/f (500 bp)

Lanes 1-11: Genotyped samples

c EphB4$^{fl/fl}$Col1A2-Cre-ERT

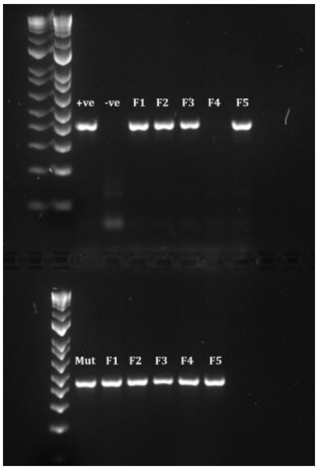

Col1a1-Cre (450 bp)

Lane 1: Positive Control
Lane 2: Negative Control
Lanes 2-7: Genotyped samples

EphB4 f/f (500 bp)

Lane 1: Positive Control
Lanes 2-6: Genotyped samples

Supplementary Figure 25. Data showing the results of PCR genotyping performed on different genetically engineered mouse models. The images include samples from (a) EFNB2$^{fl/fl}$Tie2-Cre-ERT; (b) EphB4$^{fl/fl}$Tie2-Cre-ERT; (c) EphB4$^{fl/fl}$Col1A2-Cre-ERT mice. The experiment was replicated following the generation of new pups.

Supplementary Figure 26

a

Gating strategy for intratumoral dendritic cell populations

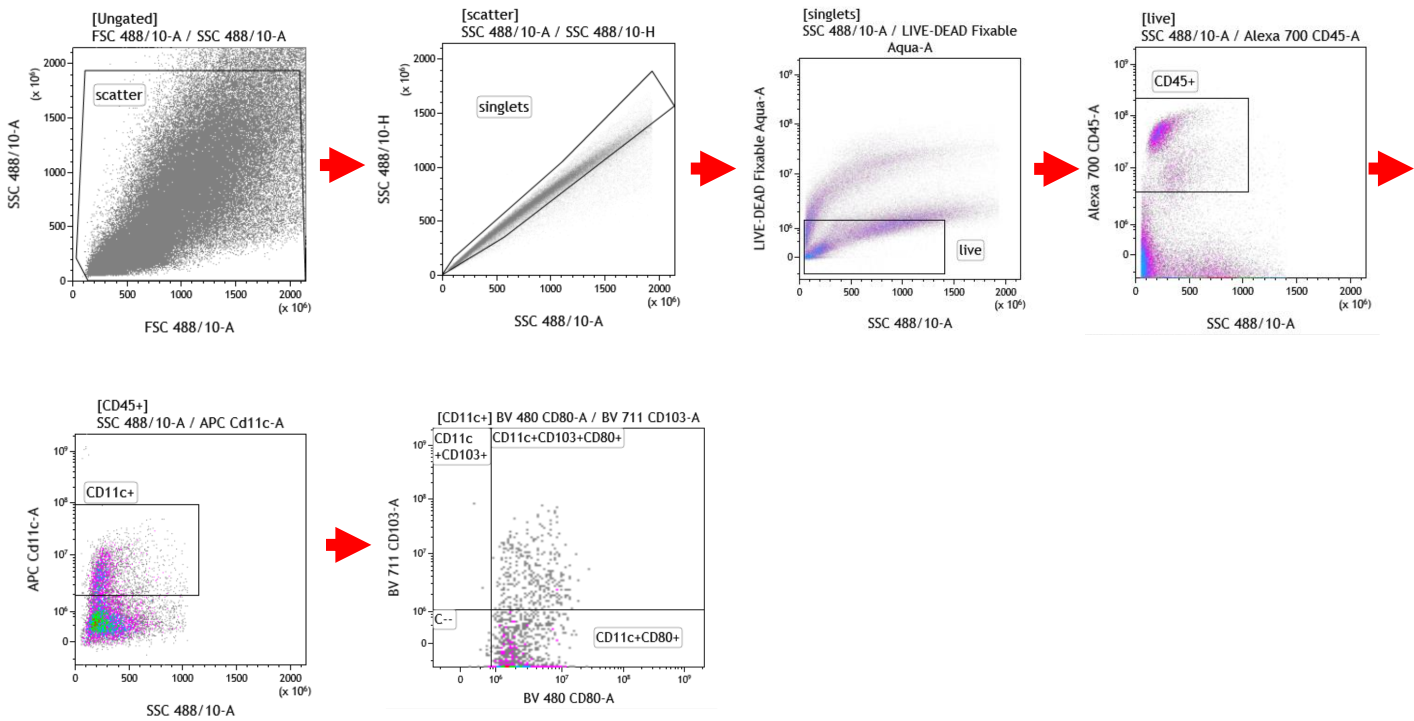

b

Gating strategy for intratumoral T cell populations

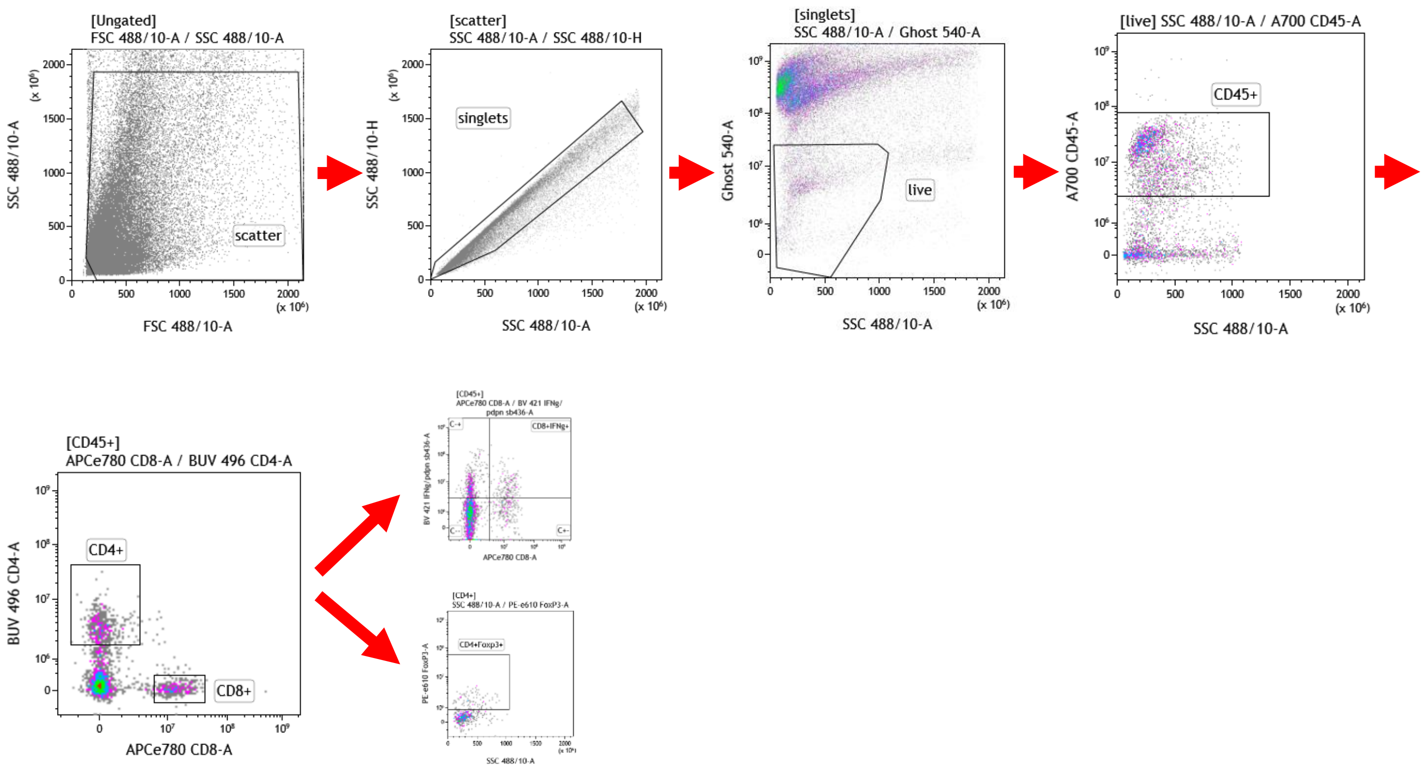

Supplementary Figure 26. Representative gating strategies for flow analysis on Moc2 tumors. The gating strategies used for intratumoral dendritic cell populations (a) and for intratumor T cell populations (b) are shown. These gating strategies have been followed for flow experiments presented in Figs 7a, 7h, and 8a.

Supplementary Figure 27

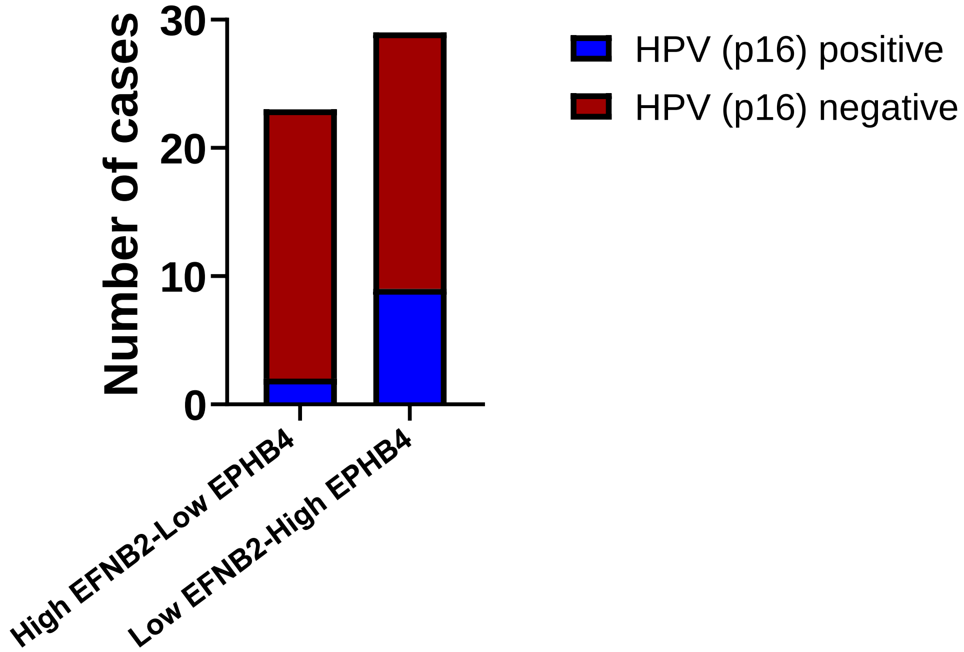

	High EFNB2 - Low EPHB4			Low EFNB2 - High EPHB4	
	n	frequency		n	frequency
HPV (p16) positive	2	9%		9	31%
HPV (p16) negative	21	91%		20	69%
Two-tailed Fisher's exact test p = 0.0859					

Supplementary Figure 27. Histogram plot representing the frequency of HPV-positive and HPV-negative head and neck cancer patients in both the high EFNB2-low EPHB4 and low EFNB2-high EPHB4 cohorts in the TCGA dataset. The exact number of cases in the high EFNB2-low EPHB4 and low EFNB2-high EPHB4 cohorts along with frequencies are mentioned in tabular format in the bottom panel.
