# Supplementary material for: EphB4 and ephrinB2 act in opposition in the head and neck tumor microenvironment
Source: Nat Commun. 2022 Jun 20;13:3535. doi: 10.1038/s41467-022-31124-7 (PMC9209511; doi:10.1038/s41467-022-31124-7)
Supplement: Supplementary file 2 — Description of Additional Supplementary Files [file 41467_2022_31124_MOESM2_ESM.pdf]

<u>Description of Additional Supplementary Files</u>

File name: Supplementary Data 1

Description: Supplementary Data 1 include z-score values for the genes involved in the angiogenesis pathway demonstrated in the main Figure 5a.

File name: Supplementary Data 2

Description: Supplementary Data 2 include z-score values for the genes involved in the cell survival pathway demonstrated in the main Figure 5b.

File name: Supplementary Data 3

Description: Supplementary Data 3 include z-score values for the IFN-gamma related Ayer's inflammatory gene signature as shown in the main Figure 5c.
