# Supplementary material for: EphB4 and ephrinB2 act in opposition in the head and neck tumor microenvironment
Source: Nat Commun. 2022 Jun 20;13:3535. doi: 10.1038/s41467-022-31124-7 (PMC9209511; doi:10.1038/s41467-022-31124-7)
Supplement: Supplementary file 6 — Reporting Summary [file 41467_2022_31124_MOESM6_ESM.pdf]

nature portfolio

Corresponding author(s): Sana D. Karam

Last updated by author(s): May 31, 2022

Reporting Summary

Nature Portfolio wishes to improve the reproducibility of the work that we publish. This form provides structure for consistency and transparency in reporting. For further information on Nature Portfolio policies, see our Editorial Policies and the Editorial Policy Checklist.

Statistics

For all statistical analyses, confirm that the following items are present in the figure legend, table legend, main text, or Methods section.

n/a	Confirmed	
☐	☒	The exact sample size (*n*) for each experimental group/condition, given as a discrete number and unit of measurement
☐	☒	A statement on whether measurements were taken from distinct samples or whether the same sample was measured repeatedly
☐	☒	The statistical test(s) used AND whether they are one- or two-sided<br>*Only common tests should be described solely by name; describe more complex techniques in the Methods section.*
☐	☒	A description of all covariates tested
☐	☒	A description of any assumptions or corrections, such as tests of normality and adjustment for multiple comparisons
☐	☒	A full description of the statistical parameters including central tendency (e.g. means) or other basic estimates (e.g. regression coefficient) AND variation (e.g. standard deviation) or associated estimates of uncertainty (e.g. confidence intervals)
☐	☒	For null hypothesis testing, the test statistic (e.g. *F*, *t*, *r*) with confidence intervals, effect sizes, degrees of freedom and *P* value noted<br>*Give P values as exact values whenever suitable.*
☒	☐	For Bayesian analysis, information on the choice of priors and Markov chain Monte Carlo settings
☒	☐	For hierarchical and complex designs, identification of the appropriate level for tests and full reporting of outcomes
☐	☒	Estimates of effect sizes (e.g. Cohen's *d*, Pearson's *r*), indicating how they were calculated

Our web collection on statistics for biologists contains articles on many of the points above.

Software and code

Policy information about availability of computer code

Data collection	Everest TM software was used to acquire flow cytometry samples on YETI equipment (Bio-Rad ZE5 cell Analyzer)
Data analysis	GraphPad Prism 9 for statistical analysis, Kaluza 2.1/ FlowJo v10.8 for flow cytometry analysis, ImageJ (NIH) software for IHC/IF analysis, InForm software for multispectral VECTRA analysis, IncuCyte Zoom software for IncuCyte experiment analysis

For manuscripts utilizing custom algorithms or software that are central to the research but not yet described in published literature, software must be made available to editors and reviewers. We strongly encourage code deposition in a community repository (e.g. GitHub). See the Nature Portfolio guidelines for submitting code & software for further information.

Data

Policy information about availability of data

All manuscripts must include a data availability statement. This statement should provide the following information, where applicable:

- Accession codes, unique identifiers, or web links for publicly available datasets
- A description of any restrictions on data availability
- For clinical datasets or third party data, please ensure that the statement adheres to our policy

The human HNSCC single cell RNA seq publicly available data used in this study are available in the Gene Expression Omnibus database under accession code GSE103322 [https://www.ncbi.nlm.nih.gov/geo/]. The murine single cell RNA seq data used in this study are available in the Gene Expression Omnibus database under accession code GSE119352 [https://www.ncbi.nlm.nih.gov/geo/query/acc.cgi?acc=GSE119352]. The HNSCC TCGA data used in this study are available in the GDC legacy archive database [https://portal.gdc.cancer.gov/legacy-archive/search/f]. The Moc2 RNA-seq tumor data generated in this study have been deposited in the Gene Expression Omnibus database under accession code GSE201148 [https://www.ncbi.nlm.nih.gov/geo/query/acc.cgi?acc=GSE201148]. The remaining data are available within the Article, Supplementary Information or Source Data file.

Field-specific reporting

Please select the one below that is the best fit for your research. If you are not sure, read the appropriate sections before making your selection.

☒ Life sciences ☐ Behavioural & social sciences ☐ Ecological, evolutionary & environmental sciences

For a reference copy of the document with all sections, see nature.com/documents/nr-reporting-summary-flat.pdf

Life sciences study design

All studies must disclose on these points even when the disclosure is negative.

Sample size	In Vivo experiments were performed using a sample size of n=6-10 mice per arm unless noted otherwise. Power analysis was used to decide on the number of mice per group. For immunofluorescence analysis, multiple fields of view per slide were quantified, and exact numbers are specified in the methods section of the paper. For the clinical data, samples were included based on tissue availability.
Data exclusions	The data represented as outliers in 4e (due to ulceration) were excluded.
Replication	The experiments were replicated using biological or technical replicates wherever applicable. Independent attempts were made to ensure data reproducibility. Number of replicates are indicated in the figure legends. Both raw tumor volumes and fold changes are reported for the tumor growth experiments.
Randomization	All mice/samples were randomized into different groups based on mice/sample availability.
Blinding	For the multispectral VECTRA staining experiments, staining and expression of markers was blinded to clinical outcome. The staining and data analysis was performed in a blinded manner. Immunofluorescence images were analyzed in a blinded manner - as experimental groups were unknown while capturing microscope images and data was analyzed using an automated script in the InForm software. Investigators were blinded during the group allocation and analysis for the experiments included in the study.

Reporting for specific materials, systems and methods

We require information from authors about some types of materials, experimental systems and methods used in many studies. Here, indicate whether each material, system or method listed is relevant to your study. If you are not sure if a list item applies to your research, read the appropriate section before selecting a response.

Materials & experimental systems

n/a	Involved in the study	
☐	☒	Antibodies
☐	☒	Eukaryotic cell lines
☒	☐	Palaeontology and archaeology
☐	☒	Animals and other organisms
☐	☒	Human research participants
☐	☒	Clinical data
☒	☐	Dual use research of concern

Methods

n/a	Involved in the study	
☒	☐	ChIP-seq
☐	☒	Flow cytometry
☒	☐	MRI-based neuroimaging

Antibodies

Antibodies used	Anti-survivin (Clone: 71G4B7; Cat#2808S), anti-Bcl-XL (Clone: 54H6; Cat#2764S), anti-p-c-Jun-Ser63 (9261S), anti-Jun (Clone: 60A8; Cat#9165P), anti-p-STAT3 (Clone: M9C6; Cat#4113S), anti-STAT3 (Clone: 124H6; Cat#9139S), Anti-EphA2 (Clone: D4A2; Cat#6997T), anti-JAK2 (Clone: D2E12; Cat#3230S), anti-CD31 (Clone: D8V9E; Cat#77699S), anti-SHP2 (Clone: D50F2; Cat#3397S), cleaved caspase 3 (Clone: Asp175; Cat#9661S), anti-NRP1 (Clone: D62C6; Cat#3725S), anti-p-SHP1 (Clone: D11G5; Cat#8849S), anti-SHP1 (Clone:C14H6; Cat#3759S), anti-beta-actin HRP conjugated (Clone: 8H10D10; Cat#12262S), and anti-PARP (Clone: 46D11; Cat#9532) antibodies were obtained from Cell Signaling Technology. Anti-EphB4 (Clone: 3D7G8; Cat#37-1800), anti-EpCAM (Clone: 323/A3; Cat#MA5-12436), anti-VEGF (Clone: VG1; Cat#MA5-12184), and anti-NG2 (PA5-100235) antibodies were purchased from Invitrogen (Carlsbad, CA, USA), and anti-ephrin-B2 (ab131536), anti-Col1A2 (ab96723), anti-alpha-SMA antibodies was obtained from Abcam (Cambridge, MA, USA). Anti-EphB4 (ABC257) antibody was purchased from MilliporeSigma and EphrinB2 (Clone: JM53-21; Cat#NBP2-66821) antibody was obtained from Novus Biologicals. Anti-Tie2 antibody (AF762) was purchased from R&D Systems; Anti-PCNA (Clone: 24/PCNA; Cat#610664) antibody was obtained from BD biosciences. Anti-VE-Cadherin (Clone: VECD1; Cat#138101) was obtained from Biolegend. Anti-EphA4 (21875-1-AP) was purchased from ProteinTech Group, Inc. Anti-p-ephrinB2 antibody (Tyr316; Cat#PA5-37552) was obtained from ThermoFisher Scientific. Goat anti-mouse (Cat#)12-349), goat anti-rabbit (Cat#RABHRP1), and donkey anti-goat (Cat#AP180P) horseradish peroxidase (HRP)-conjugated secondary antibodies were obtained from Millipore Sigma. For flow cytometry analysis, following antibodies were used: Alexa700 CD45 (Clone: 30-F11; Cat#56-0451-82), eFluor450 CD3 (Clone: 17A2; Cat#48-0032-82), APCefluor780 CD8 (Clone: 53-6.7; Cat#47-0081-82), PEe610 Foxp3 (Clone: FJK-16s; Cat#61-5773-82) from Invitrogen; BV605 CD25 (Clone: PC61; Cat#102036), BV785 CD69 (Clone: H1.2F3; Cat#104543), PerCP MHCII(clone M5/114.15.2;

	Cat#107624),PE-Cy7 NRP1 (Clone:3E12; Cat#145212), BV421 IFNg (clone XMG1.2; Cat#505830), FITC granzymeB (clone QA16A02; Cat#372206), APC Ki67 (clone 16A8; Cat#652406), APCCy7 CD11c ((clone N418; Cat#117324), BV711 CD103 (clone 2E7; Cat#121435) from Biolegend; BUV496 CD4 (612952), BV480 CD80 (746775), BUV737 CD11b (Clone: M1/70; Cat#612801) from BD Biosciences; Alexa 350 cleaved caspase 3 (Clone: 269518; Cat#IC835U) from R&D Systems; Live/dead fixable aqua dead cell stain kit (L34966) from Invitrogen or Ghost dye violet 540 for live/dead (13-0879-T100) from TONBO Biosciences.
Validation	All flow cytometry antibodies used in this study, including Viability Dye were used based on the dilutions recommended by the manufacturer or based on the data published in the literature. These antibodies have also been used in previous studies from our laboratory. For other antibody dilutions and validations, we followed recommendations available on the manufacturer's website or as optimized in the previous studies. For published reports, please see PMID: 33883256, PMID: 34518311, PMID: 30944125, PMID: 30894369, PMID: 30863843, PMID: 30042205, PMID: 27941840

Eukaryotic cell lines

Policy information about cell lines

Cell line source(s)	The cell lines were obtained as follows: Moc2 cell line from Dr. Ravindra Uppaluri (Dana-Farber Cancer Institute, Boston, MA), MEER cell line from Dr. John Lee (Sanford Health, Sioux Falls, SD) and Ly2 cell line from Dr. Nadarajah Vigneswaran (University of Texas Health Science Center, Houston, TX). CUHN013 cells were obtained from Antonio Jimeno (Anschutz Medical Campus, Aurora, CO). bEND.3 cells were obtained from the lab of Jordan Jacobelli (Anschutz Medical Campus, Aurora, CO). ATCC is the commercial source for bEND.3 cells. Ly2 cells are not available through a commercial vendor to our knowledge.
Authentication	All cell lines in this study were within 12 passages. STR analysis was conducted on the cell lines wherever applicable to ensure authentication. The murine cell lines were maintained as frozen stocks and were not cultured more than 3 weeks prior to conducting experiments to ensure phenotype stability.
Mycoplasma contamination	Cell lines were tested negative for mycoplasma contamination.
Commonly misidentified lines (See ICLAC register)	No commonly misidentified lines are included in this study.

Animals and other organisms

Policy information about studies involving animals; ARRIVE guidelines recommended for reporting animal research

Laboratory animals	Conditional knockout mouse models: Breeding pairs of EFNB2fl/flTie2-Cre-ERT, EphB4fl/flTie2-Cre-ERT, and EphB4fl/flCol1A2-Cre-ERT mice were obtained from Dr. Mohit Kapoor's lab (University Health Network, University of Toronto, Canada) and maintained at the Anschutz Medical Campus, Aurora mouse facility. Both male and female mice (7-8 weeks) were used for the genetically engineered models. For breeding mouse colony, light cycle, temperature and humidity were controlled in mouse housing area and dark cycle interruptions were avoided. A 14-hour light/10-hour dark cycle or 12 light/12 dark cycle is routinely used. Temperatures of 65-75°F (~18-23°C) with 40-60% humidity is maintained. Breeder chow diet is provided to help nursing female mice. For immunocompetent mouse model studies, 5- to 6-week-old female BALB/c mice (Charles River Laboratories) or C57BL/6 mice (Jackson Laboratories) were used. For immunocompromised mouse model studies, female athymic nude mice (5–6 weeks old, n=5–7 per group) were used. Based on our approved animal protocol (Protocol# 00250), if the implanted tumor measurement exceeded 2000 mm3 in a single plane, or if the tumors become ulcerated and mice have longer than a week remaining on the study, they were euthanized. In situations where mice had to be kept on the study if the tumor measurement exceeded 2000 mm3 limit, mice were monitored daily, applied with topical antibiotic (if there was an ulcerated tumor) and cared for by the lab personnel and the veterinary staff at the Anschutz Medical Campus. There was one mouse in the EphB4 dominant negative group (included in Figure 4a) that exceeded the 2000 mm3 limit and was monitored for longer than a week. This was an aggressively growing tumor and validation of the growth increase (versus inflammation) was essential to address the scientific question. Therefore, we had to keep this mouse under daily supervision for longer than 7 day time-period. No adverse health report was documented for this mouse during the time of extended monitoring. Euthanasia was done as per AVMA Guidelines for the Euthanasia of Animals: 2020 Edition using compressed Co2 gas inhalation method in the induction chamber followed by cervical dislocation. For ex ovo CAM assays, pre-incubated eggs (1-17 days of age) in the research-grade category were obtained from Charles River Laboratories (Norwich, CT, USA).
Wild animals	No wild animals were used in this study.
Field-collected samples	No field collected samples were used in this study.
Ethics oversight	All mice were handled and euthanized consistent with the ethics guidelines and conditions set and overseen by the University of Colorado, Anschutz Medical Campus Animal Care and Use Committee. The study has been approved by the Institutional Animal Care and Use Committee. The assays involving chicken eggs were done in ex ovo conditions and eggs were stored in optimal conditions and monitored to avoid hatching. The protocol and work flow was assessed and approved by the OLAR veterinary team at University of Colorado Denver, Anschutz Medical Campus

Note that full information on the approval of the study protocol must also be provided in the manuscript.

Human research participants

Policy information about studies involving human research participants

Population characteristics	https://clinicaltrials.gov/ct2/show/record/NCT01218048

Recruitment	https://clinicaltrials.gov/ct2/show/record/NCT01218048
Ethics oversight	Institutional Review Board of the University of Pittsburgh

Note that full information on the approval of the study protocol must also be provided in the manuscript.

Clinical data

Policy information about clinical studies

All manuscripts should comply with the ICMJEguidelines for publication of clinical research and a completedCONSORT checklist must be included with all submissions.

Clinical trial registration	NCT01218048 (UPCI 08-013)
Study protocol	https://clinicaltrials.gov/ct2/show/study/NCT01218048#studydesign. Only FFPE tissue section slides from patients enrolled in this completed clinical trial were used in the current study. Our study does not involve any direct enrollment of human patients. However, All patients were seen in the Department of Otolaryngology at the University of Pittsburgh Medical Center, and specimens from patients were obtained by informed consent under the University of Pittsburgh IRB approved protocol.
Data collection	https://clinicaltrials.gov/ct2/show/record/NCT01218048
Outcomes	https://clinicaltrials.gov/ct2/show/results/NCT01218048

Flow Cytometry

Plots

Confirm that:

- [x] The axis labels state the marker and fluorochrome used (e.g. CD4-FITC).
- [x] The axis scales are clearly visible. Include numbers along axes only for bottom left plot of group (a 'group' is an analysis of identical markers).
- [x] All plots are contour plots with outliers or pseudocolor plots.
- [x] A numerical value for number of cells or percentage (with statistics) is provided.

Methodology

Sample preparation	For flow cytometric analysis of tumor tissue, tumors were finely minced and placed in Hanks' Balanced Salt Solution (HBSS) containing 200U of Collagenase III (Worthington, Lakewood, New Jersey, USA) for 30 min at 37 degree C with gentle shaking every 10 min. After the incubation period, tumor pieces were passed through a 70 um nylon mesh. The resulting cell suspension was centrifuged and resuspended in red blood cell lysis buffer for 3 min (InVitrogen, Carlsbad, California, USA). HBSS was added to inactivate RBC lysis buffer, cell suspensions were centrifuged, resuspended and counted using an automated cell counter. For intracellular flow cytometric analysis, 2x106 cells were plated in 6-well plates and cultured for 4 hours in the presence of monensin to prevent the release of cytokines and PMA and ionomycin to stimulate cytokine production. After the incubation period, cells were incubated with a live/dead aqua viability stain kit (Invitrogen, Carlsbad, California, USA) for 30 min at 4oC. Cells were centrifuged then incubated with blocking agent FcgRIII/II (aCD16/CD32; eBioscience, San Diego, CA, USA). After blocking, cell labeling was performed by incubating cells with fluorescently conjugated antibodies.
Instrument	Flow cytometry was performed on a Yeti cell analyzer (BioRad ZE5 Cell Analyzer)
Software	Data were analyzed using Kaluza 2.1 or Flow Jo v10.8 software
Cell population abundance	No cell sorting was performed.
Gating strategy	Gating strategy is reported in the Supplementary Fig 26. The scatter plot was generated by using SSC vs FSC parameters and SSC (height) versus SSC (area) gating was applied to exclude doublets for analysis. The gate generated following the exclusion of doublets was labeled as "singlets". Singlets were then plotted using SSC vs viability dye to exclude the debris , the viable cell population is then referred as "live". This is followed by analysis using markers of interest. Viability of these populations was assessed by Live/dead fixable aqua dead cell stain kit (L34966) from Invitrogen or Ghost dye violet 540 for live/dead (13-0879-T100) from TONBO Biosciences. These gating strategies have been followed for flow experiments presented in Figs 7a, 7h, and 8a.

- [x] Tick this box to confirm that a figure exemplifying the gating strategy is provided in the Supplementary Information.
